# A macromolecular approach to eradicate multidrug resistant bacterial infections while mitigating drug resistance onset

**DOI:** 10.1038/s41467-018-03325-6

**Published:** 2018-03-02

**Authors:** Willy Chin, Guansheng Zhong, Qinqin Pu, Chuan Yang, Weiyang Lou, Paola Florez De Sessions, Balamurugan Periaswamy, Ashlynn Lee, Zhen Chang Liang, Xin Ding, Shujun Gao, Collins Wenhan Chu, Simone Bianco, Chang Bao, Yen Wah Tong, Weimin Fan, Min Wu, James L. Hedrick, Yi Yan Yang

**Affiliations:** 1 Institute of Bioengineering and Nanotechnology, 31 Biopolis Way, Singapore, 138669 Singapore; 2 NUS Graduate School for Integrative Sciences and Engineering (NGS), 28 Medical Drive, Singapore, 117456 Singapore; 3 State Key Laboratory for Diagnosis and Treatment of Infectious Diseases, First Affiliated Hospital, College of Medicine, Zhejiang University, 79 Qingchun Road, Hangzhou, 310003 China; 4 Department of Biomedical Sciences, School of Medicine and Health Sciences, University of North Dakota, Grand Forks, ND 58203 USA; 5 Genome Institute of Singapore, 60 Biopolis Street, Genome, Singapore, 138672 Singapore; 6 IBM Almaden Research Center, 650 Harry Road, San Jose, CA 95120 USA; 7 Department of Chemical and Biomolecular Engineering, National University of Singapore, 4 Engineering Drive 4, Singapore, 117576 Singapore

## Abstract

Polymyxins remain the last line treatment for multidrug-resistant (MDR) infections. As polymyxins resistance emerges, there is an urgent need to develop effective antimicrobial agents capable of mitigating MDR. Here, we report biodegradable guanidinium-functionalized polycarbonates with a distinctive mechanism that does not induce drug resistance. Unlike conventional antibiotics, repeated use of the polymers does not lead to drug resistance. Transcriptomic analysis of bacteria further supports development of resistance to antibiotics but not to the macromolecules after 30 treatments. Importantly, high in vivo treatment efficacy of the macromolecules is achieved in MDR *A*. *baumannii*-, *E*. *coli*-, *K. pneumoniae*-, methicillin-resistant *S*. *aureus*-, cecal ligation and puncture-induced polymicrobial peritonitis, and *P*. *aeruginosa* lung infection mouse models while remaining non-toxic (e.g., therapeutic index—ED_50_/LD_50_: 1473 for *A*. *baumannii* infection). These biodegradable synthetic macromolecules have been demonstrated to have broad spectrum in vivo antimicrobial activity, and have excellent potential as systemic antimicrobials against MDR infections.

## Abstract (web summary)

Antibiotic resistance is a major threat across the whole healthcare spectrum. Here, the authors report on the development of biodegradable guanidinium functionalized polycarbonates and demonstrate antimicrobial activity against drug resistant infections.

## Introduction

“ESKAPE” pathogens, including *A*. *baumannii*, *K. pneumoniae*, and *P*. *aeruginosa*, are a coterie of bacteria that exhibit a high incidence of antibiotic resistance, and are common causes of hospital-acquired infections especially in immunocompromised and critically ill patients^1^. Over the past few decades, the emergence of polymyxins resistance in these pathogens has become increasingly prevalent^2^. Despite tremendous efforts, the panacea for these recalcitrant infections has not been found. With no effective and safe treatments available, multidrug-resistant (MDR) infections are quickly morphing into a global healthcare threat. Polymyxins remain the last line treatment for these MDR Gram-negative bacterial infections although they are associated with nephrotoxicity and neurotoxicity, and are not active against Gram-positive bacteria or fungi^3, 4^. There is thus a dire need to develop novel antimicrobial compounds that have a broad spectrum of activity against both Gram-positive and -negative bacteria, yet at the same time, are well tolerated with low propensity for resistance development.

To address this problem, an unconventional class of antimicrobial agents comprising of peptides^5–7^ and synthetic polymers have recently emerged^8^. These peptides/polymers contain cationic charges that target negatively charged microbial membrane (phosphate head groups) through electrostatic interaction and hydrophobic components that disrupt lipid domains of the cytoplasmic membrane, leading to cell death (membrane-disruption antimicrobial mechanism). The balance of cationic charge (hydrophilicity) and hydrophobicity renders selectivity toward microbes as mammalian cell surface is generally neutral^9–12^. While exhibiting broad-spectrum antimicrobial activities, antimicrobial peptides possess certain inherent limitations such as high manufacturing costs and in vivo toxicity, which limits their systemic applications in the clinic although they are currently used in topical treatments^8, 13^. Significant efforts have thus been directed toward the development of synthetic antimicrobial polymers as pioneered by DeGrado and colleagues^14^, Gellman and colleagues^15^, Tew and colleagues^9, 10^, and Kuroda and colleagues^16, 17^. Most antimicrobial polymers reported in the literature have non-degradable backbones, which may potentially lead to accumulation in the body and long-term toxicity. In addition, the related studies mainly focused on structure–activity relationships through evaluation of in vitro antimicrobial and hemolytic activities. Little research has been performed on in vivo activity of antimicrobial polymers, although one paper by Kuroda’s lab reported that treatment with cationic methacrylate polymers effectively reduced the number of bacteria in a rat nasal Gram-positive *S*. *aureus* colonization model^18^. We recently synthesized biodegradable quaternary ammonium-containing amphiphilic polycarbonates as antimicrobial agents^12, 19–22^ using metal-free organocatalytic living ring-opening polymerization (ROP) methodology developed by our group^23, 24^. This polymerization strategy allows for precise control of molecular length and functionality as well as modulation of their corresponding structure–activity relationships. The polymers with optimal structures were potent against bacteria through the membrane-disruption mechanism, and effective in treating Gram-positive methicillin-resistant *S*. *aureus* (MRSA)-caused systemic infection^21, 25^. However, these polycarbonates are not active against *P*. *aeruginosa*, *A*. *baumannii*, or *K. pneumoniae*^21^. Guanidinium-rich molecules (e.g., HIV-1 TAT peptide) exhibit a membrane-translocation property, which is attributed to the ability of guanidinium to form stable multidentate hydrogen bonds with phosphate or sulfate head groups on the cell membrane^26–30^. Guanidinium-functionalized non-degradable polynorbornenes^31^ and polymethacrylates^32^ were reported to be more potent against bacteria in vitro than their amine-functionalized counterparts, and had significantly less interaction with serum proteins^32^.

In this study, polycarbonates are functionalized with guanidinium groups to provide biodegradable broad-spectrum polyguanidiniums. From octanol–water partition, bacterial membrane integrity, SEM and TEM analyses, at the effective concentration, the antibacterial function of the polymer is mainly based on membrane translocation followed by precipitation of bacterial cytoplasmic contents (e.g., proteins and genes). These polymers are demonstrated to be effective in treating MDR *A*. *baumannii*, *E*. *coli*, *K. pneumoniae*, *P*. *aeruginosa*, and MRSA infections in vivo with negligible toxicity while mitigating drug resistance.

## Results

### Polymer synthesis

**Fig. 1:**
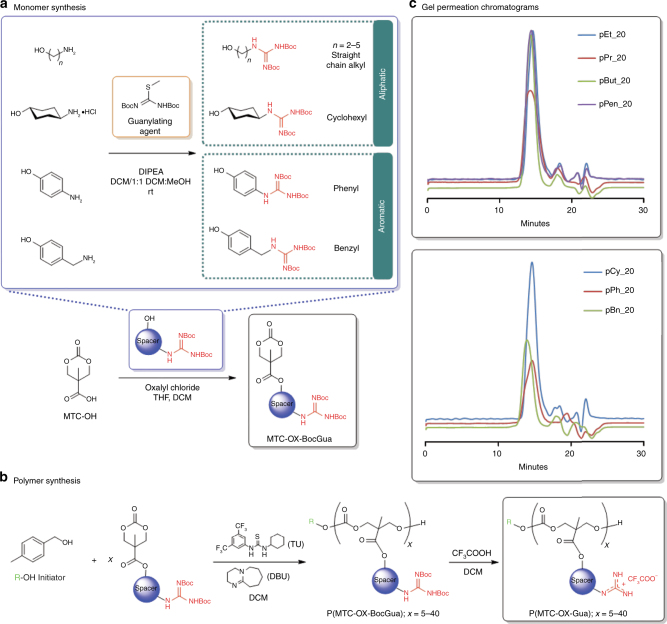
Synthesis of monomers and polymers. **a** General scheme for the modular synthesis of Boc-protected guanidine-functionalized cyclic carbonate monomers (MTC-OX-BocGua) bearing various hydrophobic spacer groups; **b** general scheme for synthesis of guanidinium-functionalized polycarbonates (P(MTC-OX-Gua)) by metal-free ROP followed by acid-mediated removal of Boc groups; **c** GPC chromatograms of representative polycarbonates (prior to deprotection)

To synthesize guanidinium-functionalized polycarbonates, guanidinium-functionalized alcohol precursors were first made by a facile, efficient, and modular route as depicted in Fig. 1a. Using 1,3-bis(*tert*-butoxycarbonyl)-2-methyl-2-thiopseudourea as the guanylating agent, the respective primary amine group belonging to a structurally diverse range (e.g., homologous straight chain alkyl, cyclohexyl, aromatic, etc.) of amino alcohols reacted readily under ambient conditions in desirable quantitative yields accompanied by the concomitant loss of a primary thiol by-product (i.e., methanethiol, MeSH) to afford the consequent Boc-protected guanylated alcohols (Supplementary Figure 1). In addition to its low cost, rapid reaction rates and high yields as compared to other conventional guanylating agents, e.g., pyrazoles^33^, the use of methylisothioureas in guanidine synthesis is advantageous in several ways^34^. Its use can be extended to the less reactive aromatic amines, such as *p-*aminophenol with good yields; there is also ease of isolation and purification upon reaction completion by virtue of the gaseous nature of the thiol by-product. The desired guanidinium-functionalized monomers were efficiently accessed through conversion of the cyclic carbonate carboxylic acid (i.e., MTC-OH) to an acid chloride intermediate in situ, followed by an esterification with the aforementioned guanylated alcohols^23, 24^ (Supplementary Figure 1). This illustrates the amenability and expeditiousness of our synthetic strategy.

**Fig. 2:**
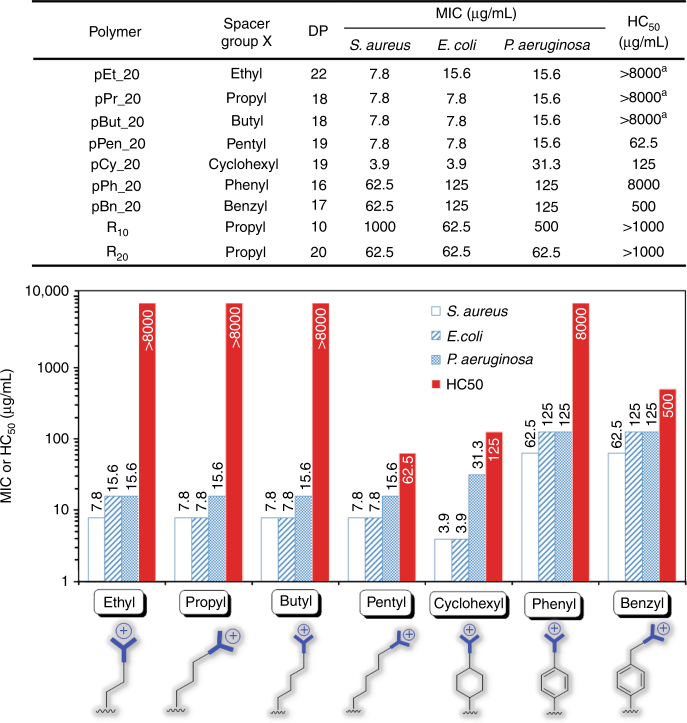
Antimicrobial (MIC) and hemolytic (HC_50_) activities. R_10_ and R_20_ represent arginine peptide with 10 and 20 amino acids, respectively. ^a^At the highest concentration (8000 µg/mL) tested, extent of hemolysis: <5%. The extent of hemolysis for pPh_20 at the threshold limit of 8000 µg/mL: ∼40%

Polymerization was subsequently carried out by metal-free organocatalytic ROP of Boc-protected guanidine-functionalized carbonate monomers containing the various requisite hydrophobic spacer moieties (Fig. 1b). In situ analysis by gel permeation chromatography (GPC) demonstrated more than 90% consumption of the respective monomer (MTC-OX-BocGua) within a remarkably short reaction time of 30 min at room temperature. By referencing the integrated intensities of relevant resonances attributed from the terminal initiator with respect to the polymer side chains, ^1^H NMR characterization of the resulting polymers elucidated that the average DP (degree of polymerization) obtained was in good agreement with that predicted from initial monomer/initiator feed ratio (Supplementary Figure 1 and Fig. 2). Furthermore, all polymers obtained exhibited narrow molecular weight distribution with a polydispersity index (Ɖ) ranging between 1.1 and 1.2, as ascertained by GPC prior to deprotection of the polymers’ Boc groups (Fig. 1c). Upon purification of the Boc-protected polycarbonates, a facile removal of the Boc groups with trifluoroacetic acid afforded the desired water-soluble guanidinium-functionalized polymers (i.e., P(MTC-OX-Gua)). Taken together, these results thus exemplify the expedient and highly controlled ability of the organocatalytic ROP for acquiring functional and well-defined polycarbonates with predictable molecular weights and narrow molecular weight distribution. Such properties are deemed particularly pivotal for an unambiguous and systematic elucidation of the role that the various structural determinants (molecular length and hydrophobic spacer) have on biological activity to optimize activity and selectivity. The polymers are labeled according to the structure of the hydrophobic spacer and the DP predicted from initial monomer/initiator feed ratio. As an example, pEt_20 denotes a guanidinium-functionalized polycarbonate containing 20 repeating units with an ethyl group as the hydrophobic spacer.

### Antimicrobial and hemolytic activities

The influence of the polymers’ molecular length was first studied on antimicrobial and hemolytic activities by varying DP from 5 to 40. The polymer pEt_20 exhibited the strongest antimicrobial activity against a broad panel of pathogenic microbes with minimum inhibitory concentrations (MICs, the lowest polymer concentrations that completely inhibited microbial growth) in the range of 7.8–15.6 µg/mL and the lowest geometric mean (*G*_m_) value of MICs (Supplementary Table 1). The MICs of pEt_20 were much lower than the previous antimicrobial polycarbonates bearing quaternary ammonium groups^12, 19–22^. Remarkably, pEt_20 possessed a pronounced lack of toxicity toward rat red blood cells (HC_50_, polymer concentration that induces 50% hemolysis: >8000 μg/mL) (Supplementary Table 1). This is the highest HC_50_ value that has ever been reported for biodegradable synthetic antimicrobial polymers^12, 19–22^. Serum content was less than 2% in the hemolysis assay. The MIC values of pEt_20 (and pEt_10) against the panel of bacteria were determined to be the same in the presence of 2% serum. Lack of interaction with proteins was possibly due to diffused charge in the guanidinium group. Its selectivity (HC_50_/*G*_m_ and HC_5_/*G*_m_, >584) is significantly higher than those of many host defense peptides, e.g., cecropin A (HC_5_/*G*_m_ = 0.034)^35^, porcine protegrin-1 (HC_50_/*G*_m_ = 2.3)^35^, magainins (HC_50_/MIC = 10)^36^ and defensins (rMicasin: HC_5_/*G*_m_ = 4)^37^, synthetic peptides^38, 39^, guanidinium-functionalized non-degradable polynorbornenes (HC_50_/G_m_, 75)^31^, and polymethacrylates (HC_5_/*G*_m_, <2)^32^, and recently reported star-shaped peptide PAMAM-[poly(lysine-*co*-valine)]_32_ (HC_5_/*G*_m_, 23)^40^. Moreover, pEt_20 and pEt_10 had significantly lower cytotoxicity against human embryonic kidney (HEK293T) cells after 18-h incubation than polymyxin B (Supplementary Figure 2) and PAMAM-[poly(lysine-*co*-valine)]_32_ (shorter incubation time: 90 min)^40^ (IC50, inhibitory concentration of compound that leads to 50% cell viability: >500, >500, 165, and 128 μg/mL, respectively).

Having established the optimal DP to be 20 for the highest potency and selectivity, the influence of the hydrophobic spacer was further investigated for a series of polymers bearing different spacer group structures encompassing of aliphatic and aromatic types (Fig. 2). The alkyl series (pEt_20, pPr_20, and pBut_20) had greater antimicrobial potency and higher HC_50_ than the aromatic ones (pPh_20 and pBn_20). Increasing the alkyl length from ethyl to pentyl or cyclohexyl led to a dramatic increase in hemolysis. This is largely attributed to the corresponding increase in polymer’s hydrophobicity that might enhance its hydrophobic interaction with cell membrane, facilitating membrane disruption. Although oligoarginine peptide R_20_ has the same number of guanidinium groups and the same hydrophobic spacer as pPr_20, its antimicrobial activity was significantly lower (Fig. 2). R_10_ was even less potent. This stark difference might be due to enhanced membrane translocation of polymer and/or stronger interaction between the polymer and cytoplasmic proteins/genes stemming from the more hydrophobic polycarbonate backbone as compared to peptide backbone. As pEt_10 and pEt_20 exhibited strong antimicrobial activity and high selectivity, they were chosen for further studies.

### Antimicrobial activity against clinically isolated MDR bacteria

**Fig. 3:**
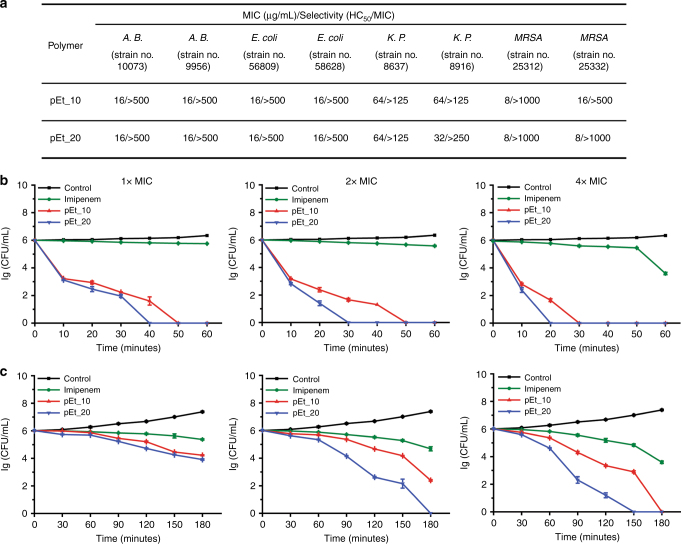
Antibacterial activity of guanidinium-functionalized polycarbonates. **a** MIC and selectivity (HC_50_/MIC) of polymers against clinically isolated multidrug-resistant bacteria (*A*. *B*.*: A*. *baumannii*; *K*. *P*.*: Klebsiella pneumoniae*; MIC of R_10_ and R_20_: >512 µg/mL against all the strains of bacteria tested); **b** killing kinetics of *A*. *baumannii* 10073 at different concentrations as specified; **c** killing kinetics of *E*. *coli* 56809 at different concentrations as specified. The antibiotic imipenem did not exert significant bactericidal activity against *A*. *baumannii* and *E*. *coli* after 1 and 3 h treatment, respectively, while the polymers eradicated the bacteria rapidly. An increased polymer concentration led to faster killing efficiency. Error bars represent s.d. for *n* = 3

To evaluate the potential of the polymers as effective antimicrobials for future clinical applications, pEt_10 and pEt_20 were assayed against four of the most opportunistic and MDR bacteria in both developing and developed healthcare systems, Gram-negative *A*. *baumannii*, *K. pneumoniae*, and *E*. *coli* as well as Gram-positive MRSA (Supplementary Table 2). These bacteria are part of “ESKAPE” pathogens^1^, and are resistant to multiple antibiotics including polymyxin B (Supplementary Table 2). The polymers demonstrated efficacious antimicrobial activity and favorable selectivity toward the clinically isolated MDR bacteria (Fig. 3a), in concordance with efficacy seen against commercially available ATCC strains (Fig. 2). In sharp contrast, the oligoarginine peptides R_10_ and R_20_ were not effective against the bacteria even at 512 µg/mL. The presence of 10% serum did not alter the MIC values of both pEt_10 and pEt_20 against these MDR clinical isolates. A similar phenomenon was also observed in guanidinium-functionalized non-degradable polymethacrylates^32^. In the presence of 40% serum, MIC increased by 1–7 times (1 time for MRSA and *A*. *baumannii*, 1–3 times for *K. pneumoniae*, and 7 times for *E*. *coli*). The increases in MIC for MRSA and ATCC *S*. *aureus* (1 time) were lower than those for small molecular synthetic foldamers against ATCC *S*. *aureus* (31–63 times increase in MIC in the presence of 40% serum), which showed high in vivo efficacy in a *S*. *aureus*-caused mouse thigh infection model^41^, indicating that pEt_10 and pEt_20 had less protein interaction than the foldamers.

### In vitro killing efficiency and kinetics

The antimicrobial activity of the polymers was further investigated by colony forming unit (CFU) assay against a representative strain of clinically isolated MDR bacteria, *A*. *baumannii* 10073, *E*. *coli* 56809, *K. pneumoniae* 8637, and MRSA 25312, in comparison with antibiotics imipenem (control for the Gram-negative bacteria) and vancomycin (control for MRSA). The *A*. *baumannii* and *K. pneumoniae* strains were resistant to imipenem, whereas the *E*. *coli* and MRSA strains were susceptible to imipenem and vancomycin, respectively (Supplementary Table 3). Like imipenem, at 1× MIC, 2× MIC, or 4× MIC, pEt_10 and pEt_20 demonstrated ∼100% killing efficiency against *A*. *baumannii* (Supplementary Figure 3). In the case of *E*. *coli* and *K. pneumoniae*, they achieved more than 99.9% efficiency at 2 × MIC and 1 × MIC, respectively (Supplementary Figure 3). Killing efficiency of vancomycin against MRSA was less than 99% (2-log reduction in bacterial counts) even at 4 × MIC, while pEt_10 and pEt_20 eliminated more than 99.9% MRSA (3-log reduction in bacterial counts) at 2 × MIC and 1 × MIC, respectively. These results clearly demonstrated bactericidal activity of the polymers against both Gram-positive and Gram-negative MDR bacteria. Although imipenem and vancomycin were shown to be bactericidal (more than 3-log reduction in bacterial counts) at 1 × MIC and 2-log reduction in bacterial counts at 2 × MIC, respectively, their efficacies were only observed after 18 h of treatment (Supplementary Figure 3). They were unable to eradicate 99.9% bacteria after 1 h (*A*. *baumannii*), 2 h (*K. pneumoniae*), or 3 h (MRSA, *E*. *coli*) of treatment even at 4 × MIC (Fig. 3, Supplementary Figure 4, and Supplementary Figure 5). In sharp contrast, pEt_10 and pEt_20 eliminated these MDR bacteria effectively even at shorter treatment durations (Fig. 3, Supplementary Figure 4, and Supplementary Figure 5). For example, pEt_20 eradicated ~99.9% *A*. *baumannii* at 1 × MIC within 10 min (Fig. 3b). An increase in polymer concentration resulted in faster elimination of bacteria. In addition, pEt_20 with a longer polymer chain eradicated bacteria more efficiently especially at higher concentrations. Such promising in vitro results with these polymers certainly warrant further in vivo studies for the treatment of MDR infections.

### Antimicrobial mechanism

**Fig. 4:**
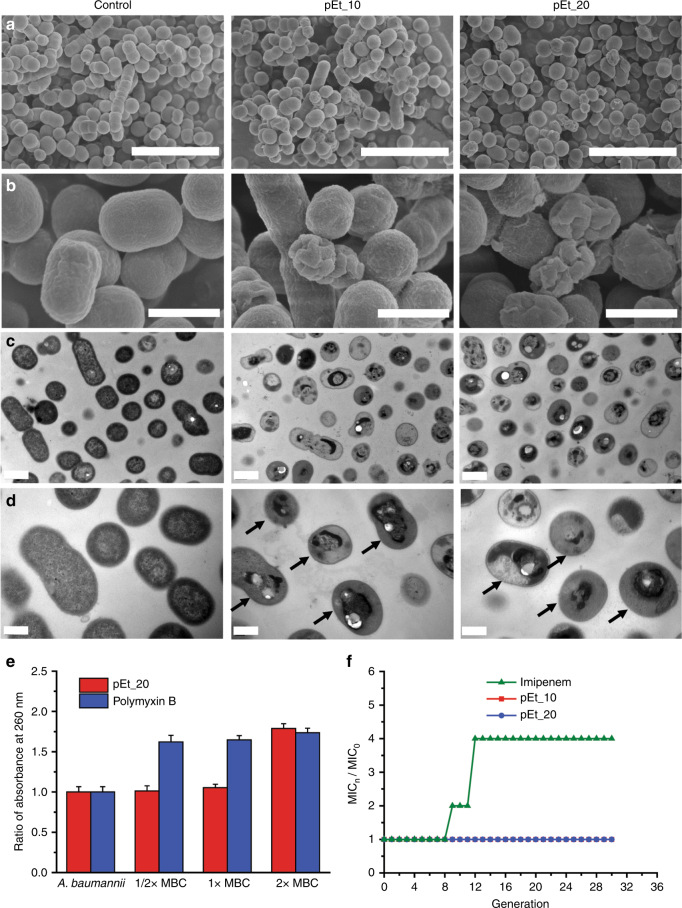
Analyses of mechanisms of the antimicrobial polymers. Scanning electron microscopic (FE-SEM) (**a**, **b**) and transmission electron microscopic (TEM) (**c**, **d**) images of *A*. *baumannii* 10073 with and without polymer treatment. Treatment conditions: 4 × MIC, 2 h. Scale bar: SEM—5 µm in **a** and 1 µm in **b**; TEM—1 µm in **c** and 0.5 µm in **d**. **e** Ratio of absorbance at 260 nm of supernatant in *A*. *baumannii* 10073 suspension treated with pEt_20 or polymyxin B for 2 h at different concentrations as specified (bacterial density: 2.08 × 10^9^ CFU/mL). Ratios above 1.0 reflect release of cytoplasmic materials of bacteria. MBC for pEt_20 and polymyxin B: 32 and 256 µg/mL, respectively. **f** Drug resistance development profiles of *A*. *baumannii* 10073 after exposed to the polymers and the clinically used antibiotic imipenem at sub-MIC concentrations. At the effective dose (MBC), polymer treatment did not cause significant release of cytoplasmic materials while polymyxin B disrupted cell membrane, leading to significant leakage. This is consistent with SEM observation, where the majority of the cells did not see membrane lysis although a few cells had distorted membrane. Precipitation of cytoplasmic materials was seen in most cells with an intact membrane under TEM. Taken together, these observations suggested that antimicrobial mechanism is mainly based on membrane translocation followed by precipitation of cytoplasmic materials through interactions between the polymer and proteins/genes in the cytoplasm. This unique antibacterial mechanism prevented drug resistance development. **e** Error bars represent s.d. for *n* = 3. **f** The data are representative of three replicates

To shed light on the mode of action of the guanidinium-functionalized polycarbonate, a number of techniques were employed to understand polymer-bacterial membrane interaction and cause of efficient cell death with high selectivity. Octanol–water partition study was first performed using the fluorescent dye dansyl-labeled pEt_20 and corresponding polycarbonate containing quaternary ammonium (Supplementary Figure 6). Both polymers partitioned almost exclusively into the aqueous phosphate-buffered saline (PBS) layer as a consequence of the charged nature of the polymers. However, upon the addition of a model surrogate for a membrane-bound fatty acid salt (sodium laurate) into the octanol layer, pEt_20 was seen to partition significantly into the octanol layer with just 0.5 equivalents of sodium laurate. As the concentration of the fatty acid salt increased, pEt_20 was virtually partitioned only within the octanol layer. In contrast, the quaternary ammonium polymer stayed in the aqueous layer even at 2.0 equivalents of the fatty acid salt added. This finding showed that pEt_20 effectively translocated into the membrane-mimic lipophilic layer. Next, the integrity of bacterial membrane was studied after treatment with pEt_20 or the membrane-lytic polymyxin B by testing leakage of cytoplasmic materials (e.g., proteins and genes) with absorbance at 260 nm. No significant leakage of cytoplasmic materials was detected from *A*. *baumannii* cells after 2-h pEt_20 treatment at MBC (minimum bactericidal concentration that leads to 99.9% bacteria killing in 2 h) and below although leakage was seen at a higher concentration (2 × MBC) (Fig. 4). This indicated that pEt_20 killed the bacteria without lysing membrane at the effective dose. In contrast, significant membrane leakage was observed with polymyxin B treatment even at 0.5 × MBC.

The morphological changes in *A*. *baumannii* were further observed under scanning electron microscopy (SEM) before and after polymer treatment. No membrane lysis was seen in the majority of the cells after 2-h treatment with either pEt_10 or pEt_20 at 4 × MIC, and only a few cells had distorted membrane (Fig. 4a, b). Although prolonging the treatment time from 2 to 6 h led to more cells with disrupted membrane, a larger population of cells had an intact membrane (Supplementary Figure 7). Similar phenomena were also observed under transmission electron microscopy (TEM) after 2-h polymer treatment at 4 × MIC and 16 × MIC (Fig. 4c, d and Supplementary Figure 7). Particularly, the TEM micrographs of polymer-treated bacteria revealed precipitation of the bacterial cytoplasm with an intact membrane in a larger population of the cells at both concentrations (see arrows in Fig. 4c, d and Supplementary Figure 7), which was likely caused by polymer interactions with cytoplasmic materials (e.g., proteins and genes) upon membrane translocation. Similarly, non-biodegradable broad-spectrum antimicrobial polyhexamethylene biguanide was recently reported to enter bacterial cells, bind DNA and condense chromosome, leading to cell death^42^. This unique antimicrobial mechanism provides the polymers with higher potency and greater selectivity than quaternary ammonium-containing polycarbonates that function based on the membrane-disruption mechanism (Fig. 2)^12, 19–22^. Moreover, another key consideration with this novel mechanism over membrane lysis is the minimization of possibility of septicemia from the rapid release of cytosolic content. This distinctive mechanism warrants further investigation and will be the topic of future studies as it distinguishes this new class of polymers from antimicrobial peptides that have been widely studied.

### Prevention of resistance development

To investigate if repeated use of the polymers with the unique antimicrobial mechanism develops resistance, *A*. *baumannii* 10073 cells were treated with pEt_10 or pEt_20 for 30 passages at sub-MIC concentrations and MIC was determined in each passage of the bacteria. As shown in Fig. 4e, repeated use of these polymers did not cause resistance development in *A*. *baumannii* even after 30 passages, while multiple treatments with imipenem at sublethal doses developed resistance after 8 passages as a result of reduced antimicrobial effect. A similar phenomenon was also observed previously for the antibiotics gentamicin in *E*. *coli*^43^ and vancomycin in MRSA^25^, and for gentamicin in *K. pneumoniae* as shown in Supplementary Figure 8.

### RNA-seq analysis of bacterial resistance

**Fig. 5:**
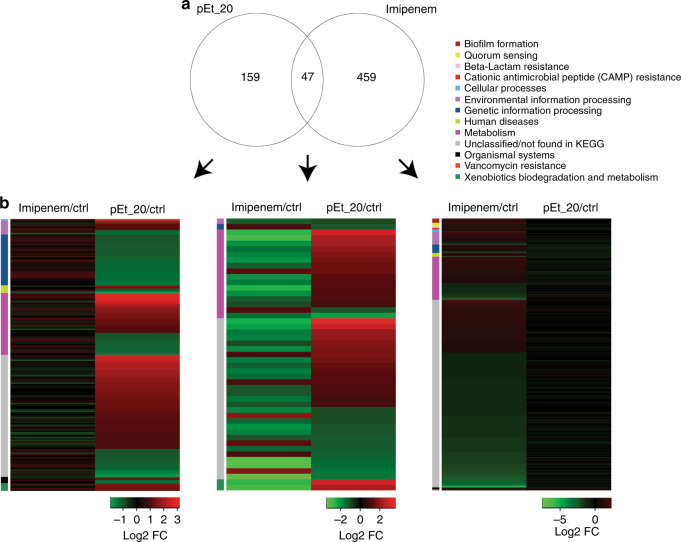
RNA-seq analysis of bacterial resistance. MDR *A*. *baumannii* 10073 was treated with imipenem or pEt_20 for 30 passages at 0.5× MIC that was measured at each passage (treatment duration of each passage: 18 h). **a** A Venn diagram depicting the overlap of differentially regulated genes either upon pEt_20 or imipenem treatment, relative to untreated controls. The numbers inside the Venn diagram (from left to right) represent the number of differentially regulated genes: found only in pEt_20 treatment (left), common to both groups (venn diagram intersection), only in imipenem treatment (right), respectively. **b** The three independent heat maps depict genes from the corresponding groups in **a** (as indicated by the respective black arrows). The heat map on the left shows expression data for genes that are only significant in pEt_20 treatment but were shown in conjunction with the expression data from imipenem treatment (not differentially regulated, i.e., Log_2_-fold change in the range between −1 and +1). The heat map in the center depicts significant genes that are shared by both treatment groups. The heat map on the right shows genes that are only significant in imipenem treatment along with Log_2_-fold change from pEt_20 treatment (not differentially regulated but shown for comparison). The row color side bars on all three heat maps represent the KEGG categories that the genes represent

In order to systematically understand bacterial response to different xenobiotic stress, i.e., antibiotic or polymer, bacterial RNA-seq was performed on MDR *A*. *baumannii* 10073 cultures after 30 passages. An MDR strain AB030 was used to recapitulate known imipenem response genes as a proof of concept and to compare it to pEt_20 treatment. A total of 506 genes were differentially regulated [Log_2_-fold change 1; 5% false discovery rate (FDR)] upon imipenem treatment relative to the untreated control (Supplementary Data 1 and Fig. 5). KEGG pathway analyses of the significant genes implicated pathways that are involved in beta-lactam, vancomycin, and cationic antimicrobial peptide resistances. Importantly, the predominant efflux pump-mediated carbapenem resistance genes from the resistance nodulation (RND) family, i.e., *adeA* and *adeB*, were both upregulated upon imipenem treatment^44, 45^. In addition, *ftsI* (penicillin binding protein 3) was also shown to be differentially regulated, possibly conferring carbapenem resistance^46^. We were also able to recapitulate some of the known categories implicated in imipenem resistance including quorum sensing and biofilm formation^44, 47^. However, in the case of pEt_20, there were fewer differentially regulated genes that were significant (*n* = 206; Supplementary Data 2, Fig. 5, Log_2_-fold change 1; 5% FDR) and no apparent transcriptional response in any known resistance pathways (KEGG database) were observed relative to pEt_20 treatment.

A systematic comparison of imipenem and pEt_20 response genes resulted in 47 common genes (Fig. 5a). Interestingly, a majority (~78%) of these common genes show opposite patterns for pEt_20 and imipenem treatments (Fig. 5b). Overall, the above data suggest that imipenem and polymer response genes have mutually exclusive functions, while the polymer has no observed propensity for resistance development. This merits in effective treatment possibilities for polymer-based antimicrobial agents to combat resistance.

### Evaluation of in vivo toxicity and immunogenicity

**Fig. 6:**
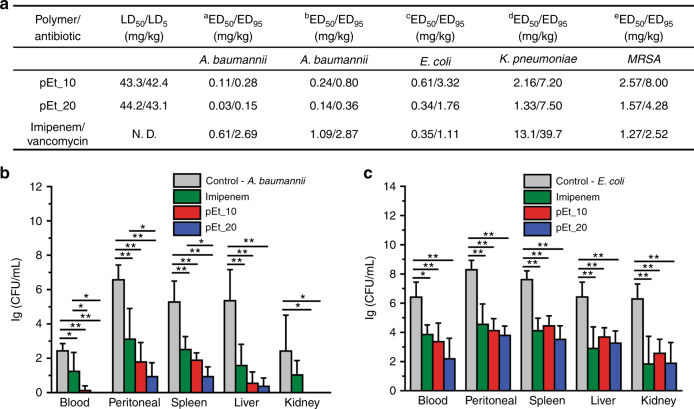
In vivo antibacterial efficacy in various infectious mouse models. **a** ED_50_ and ED_100_ of polymers and the imipenem or vancomycin control (a: MDR *A*. *baumannii* 10073-caused peritonitis mouse model, intraperitoneal (i.p.) injection at 1 h and 6 h post infection; b: MDR *A*. *baumannii* 10073-caused peritonitis mouse model, i.p. injection at 3 h and 8 h post infection; c: MDR *E*. *coli* 56809-caused peritonitis mouse model, i.p. injection at 1 h and 6 h post infection; d: MDR *K*. *pneumoniae* 8637-caused peritonitis mouse model, i.p. injection at 1 h and 6 h post infection; e: MDR *MRSA* 25312-induced peritonitis mouse model, i.p. injection at 1 h and 6 h post infection). Colony forming units (CFUs) of *A*. *baumannii* 10073 (**b**) and *E*. *coli* 56809 (**c**) in blood, peritoneal cavity, spleen, liver, and kidney at 24 h post infection. Both polymers were effective against systemic MDR Gram-negative bacterial infections with lower ED_50_/ED_95_ values than imipenem (especially *A*. *baumannii* 10073- and *K*. *pneumoniae* 8637-caused infections), and they also worked against Gram-positive *MRSA* infection with comparable ED_50_/ED_95_ values as compared to vancomycin. The polymers removed bacteria in the blood, peritoneal cavity, and organs more effectively than imipenem especially in the *A*. *baumannii* 10073 infection. Means ± s.d., *n* = 5. One-way ANOVA (Tukey’s post hoc); *p < 0.05; **p < 0.01

To evaluate these polymers for in vivo application to treat drug-resistant infections, LD_50_/LD_5_ (single lethal dose resulting in 50% and 5% mice mortality, respectively) values of pEt_10 and pEt_20, were first determined. No significant differences in toxicity were observed with both polymers as evidenced by similar LD_50_ and LD_5_ values (Fig. 6a). In addition, immunogenicity of the polymers was evaluated in mouse peripheral blood mononuclear cells (PBMCs) by testing secretion of the pro-inflammatory cytokines IFN-γ and TNF-α by PBMCs after polymer treatment. The secretion of the cytokines may result in undesirable non-specific immunogenic response in vivo. The treatment with the bacterial lipopolysaccharides (LPS) stimulated a high level of IFN-γ and TNF-α secretion, while pEt_10 or pEt_20 treatment showed negligible immunogenic response as compared to the negative control without any treatment (Supplementary Figure 9).

### In vivo biodistribution and pharmacokinetics

From the biodistribution study of a NIR dye (AF750)-labeled pEt_20 (Supplementary Figure 10), the polymer was found in mouse liver, spleen, lungs, and kidneys after i.p. or i.v. injection, indicating that the polymer penetrated tissues and got into the blood stream even after i.p. injection. The pharmacokinetics study of the dye-labeled polymer demonstrated its mean plasma elimination half-life of ∼17 min (Supplementary Tables 4 and 5), which is comparable to (if not longer than) that of the clinically used antibiotics meropenem (6 min), imipenem (12 min), and doripenem (12 min)^48^. Taken together with rapid killing kinetics (Fig. 3b, c, Supplementary Figure 4, and Supplementary Figure 5), the polymers have potential utility in vivo.

### In vivo antimicrobial activity

The in vivo treatment efficacy of the polymers was then investigated in *A*. *baumannii* 10073-, *E*. *coli* 56809-, *K. pneumoniae* 8637-, and *MRSA* 25312-induced peritonitis mouse models. The minimum lethal doses of *A*. *baumannii*, *E*. *coli*, *K. pneumoniae*, and *MRSA* sufficient to cause 100% mortality within 48 h through intraperitoneal (i.p.) injection were pre-determined: 2.7 × 10^8^, 8.8 × 10^6^, 2.6 × 10^8^, and 5.2 × 10^8^ CFU/mL (0.5 mL), respectively. Mice were inoculated with the bacteria at their respective lethal doses by i.p. injection, and subsequently treated with two i.p. injections of pEt_10, pEt_20, imipenem (control for the Gram-negative bacteria), or vancomycin (control for *MRSA*) at 1 and 6 h or 3 and 8 h post infection. Both polymers demonstrated high treatment efficacy across all four infection models with low ED_50_/ED_95_ (effective dose resulting in 50 and 95% survival of infected mice) values, which were well below their LD_5_ values especially in *A*. *baumannii* infection (Fig. 6a), showing a wide therapeutic window (e.g., therapeutic index—ED_50_/LD_50_: 1473 for *A*. *baumannii* infection). Delay in the treatment by 2 h slightly increased ED_50_/ED_95_ values. In imipenem-susceptible *E*. *coli* and vancomycin-susceptible *MRSA* infections, the polymers had similar ED_50_/ED_95_ as compared to the antibiotics. However, in imipenem-resistant *A*. *baumannii* and *K. pneumoniae* infections, the polymers displayed higher efficacy with lower ED_50_/ED_95_ than imipenem. In addition, pEt_20 achieved better treatment efficacy than pEt_10 (lower ED_50_/ED_95_ for pEt_20). This may be in part attributable to pEt_20’s superior bactericidal kinetics (Fig. 3b, c, Supplementary Figure 4, and Supplementary Figure 5).

In vivo elimination efficiency of bacteria was subsequently assessed in *A*. *baumannii* 10073- and *E*. *coli* 56809-induced peritonitis mouse models. The polymers and imipenem effectively removed bacteria from the blood, peritoneal fluid, and organs with >99.0–99.9% efficiency at their respective ED_95_ doses as compared to the control without any treatment at 24 h post infection (Fig. 6b, c). Particularly, in the *A*. *baumannii* infection, pEt_20 exhibited higher efficacy than imipenem (Fig. 6b) primarily because pEt_20 had faster killing kinetics (Fig. 3b). Effective removal of bacteria is key to ensuring survival against bacterial sepsis. This limits the production and consequent circulation of deleterious bacterial endo- and exo-toxins otherwise responsible for septic shock and multi-organ dysfunction syndrome.

**Fig. 7:**
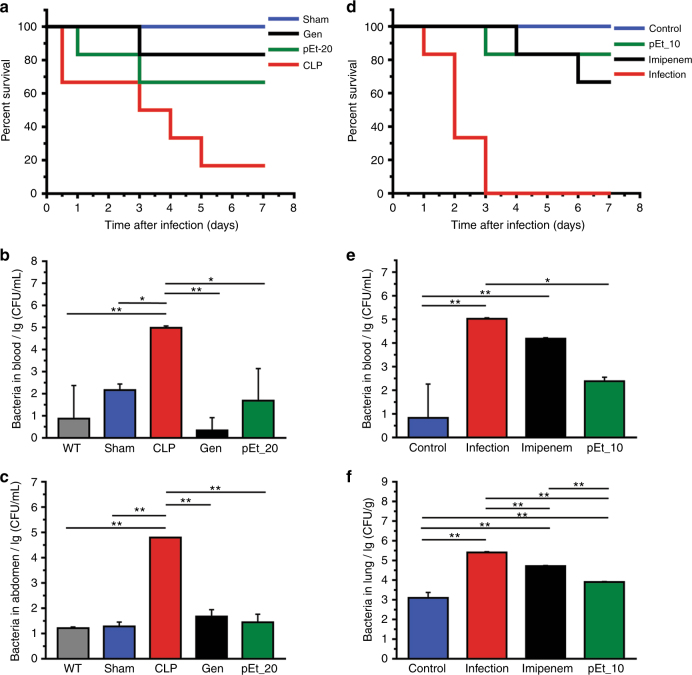
In vivo efficacy in CLP and *P*. *aeruginosa* lung infection models. **a**–**c** Cecal ligation and puncture (CLP) model; **d**–**f** *P*. *aeruginosa* (PA14)-caused lung infection model. **a** Survival of mice in the sham group that went through surgery but without CLP, in the CLP group without treatment, in the gentamicin-treated CLP group (Gen, 10 mg/kg mouse body weight at 1 h and 6 h post infection), and in the pEt_20-treated group (pEt_20, 25 mg/kg mouse body weight at 1 h and 6 h post infection; each dose lower than LD_5_ value) (*n* = 6, *p* = 0.0112 (<0.05), Log-rank test). **b** Microbial counts (CFUs) in the blood and peritoneal fluid (**c**) at 24 h after CLP. WT: control group without surgery or treatment. Means ± s.d., *n* = 3. **d** Survival of mice with *P*. *aeruginosa* (PA14)-caused lung infection without and with pEt_10 or imipenem treatment (8 mg/kg mouse body weight at 1, 6, and 25 h post infection) (*n* = 6, *p* = 0.0214 (<0.05), Log-rank test). **e** Bacterial counts (CFUs) in the blood and the lung tissues (**f**). Means ± s.d., *n* = 3. One-way ANOVA (Tukey’s post hoc); *p < 0.05; **p < 0.01. Control group: without infection or treatment

The treatment efficacy of polymer was further evaluated in a cecal ligation and puncture (CLP)-induced polymicrobial peritonitis mouse model, the most commonly used sepsis model with comparable features of peritonitis in humans, where a systemic infection was induced by release of fecel material into peritoneal cavity^49^. As there are both Gram-positive and Gram-negative bacteria present in fecal material, the broad-spectrum antibiotic gentamicin was used as control. With a single day treatment, pEt_20 achieved comparable in vivo efficacy as compared to gentamicin in terms of mouse survival (Fig. 7a) and bacterial removal efficiency (Fig. 7b, c).

To demonstrate in vivo efficacy in treating a distal infection, the polymer was used to treat a clinical strain of *P*. *aeruginosa* (PA14)-caused lung infection in a mouse model by intravenous injection (MIC of pEt_10 against PA14: 25 µg/mL; 8 mg/kg of mouse body weight per injection, 3 i.v. injections at 1, 6, and 25 h post infection). A single day treatment with pEt_10 saved five out of six infected mice, while imipenem treatment saved four infected mice (Fig. 7d). In addition, the polymer was more effective in removing the bacteria from the blood and the lung tissues (Fig. 7e, f).

More importantly, mice treated with the polymers at 8 mg/kg mouse body weight, 0.2 mL per injection, twice daily for 3 consecutive days (48 mg/kg in total), which is close to the highest dose used in this study (50 mg/kg), did not induce nephro- or hepato-toxicity, nor any electrolyte disturbances (Supplementary Table 6). There was also negligible hemolysis as evidenced by normal blood potassium levels. This may be explained by complete degradation of the polymer after 3 days (Supplementary Figure 11), and negligible toxicity of the degradation products (Supplementary Figure 12). These results suggest negligible polymer toxicity especially when administered at their effective dosages.

In this study, we have demonstrated high in vivo efficacy of the guanidinium-functionalized polycarbonates with broad-spectrum activity including MDR *A*. *baumannii-*, *K*. *pneumonia-*, *E*. *coli-*, *MRSA-*, *P*. *aeruginosa-*, and CLP-induced polymicrobial systemic infections, and *P*. *aeruginosa* lung infection. The multiple treatments of bacteria with the polymers do not develop drug resistance, owing to a distinctive new mechanism, as evidenced in an in vitro evolution model and genetic sequencing. Polymer treatment at the effective doses does not cause acute systemic toxicity in vivo. It is envisaged that these polymers bear great potential as antimicrobial agents in the prevention and treatment of multidrug-resistant systemic infections.

## Methods

### Materials

*N*-(3,5-trifluoromethyl) phenyl-*N*′-cyclohexylthiourea (TU) was prepared according to our previous protocol^23, 24^. TU was dissolved in dry tetrahydrofuran (THF), stirred with CaH_2_, filtered, and freed of solvent in vacuo. Prior to use, 1,8-diazabicyclo [5,4,0]undec-7-ene (DBU) was stirred over CaH_2_ and vacuum distilled before being transferred to a glove box. All other chemical reagents were purchased from Sigma-Aldrich and used as received unless specified. Ultra pure (HPLC grade) water was obtained from J.T. Baker (USA). Phosphate-buffered saline (PBS) at 10× concentration was purchased from first BASE (Singapore) and diluted to the intended concentrations before use. Cation-adjusted Mueller–Hinton broth (MHB) powder was bought from BD Diagnostics (Singapore) and used to prepare the microbial broths according to the manufacturer’s instructions. *Staphylococcus aureus* (ATCC No. 6538), *Escherichia coli* (ATCC No. 25922), *Pseudomonas aeruginosa* (ATCC No. 9027), and yeast *Candida albicans* (ATCC No. 10231) were obtained from ATCC (USA) and reconstituted according to the suggested protocols. Clinically isolated MDR bacteria that were extracted from blood or phlegm samples of the patients hospitalized in The First Affiliated Hospital of Medical College, Zhejiang University (Hangzhou, China) were kindly provided by Dr. Kaijin Xu. All isolates were identified by routine laboratory methods and stored in 20% (v/v) glycerol at −80 °C prior to use. Drug susceptibility of the bacteria was evaluated according to Clinical Laboratory Standards Institute (CLSI) (Supplementary Table 2).

### Synthesis of Boc-protected guanylated alcohol precursors

For the guanylation of amino alcohols, a detailed protocol for the synthesis using ethanolamine as the starting reagent is described as a representative example (Figure 1a). To a solution mixture of ethanolamine (1.37 mL, 22.8 mmol, 2.0 equiv) and *N*,*N*-diisopropylethylamine (DIPEA) (6.0 mL, 34.4 mmol, 3.0 equiv) was added 1,3-bis(*tert*-butoxycarbonyl)-2-methyl-2-thiopseudourea (3.3 g, 11.6 mmol, 1.0 equiv) in 20 mL of dry CH_2_Cl_2_, and the mixture was left to stir overnight at room temperature. Upon reaction completion, a constant stream of nitrogen gas was bubbled through the reaction mixture for ~1 h so as to aid in purging of the gaseous by-product, MeSH. After the removal of residual solvent in vacuo, the crude product was purified by flash column chromatography using silica gel and a hexane-ethyl acetate solvent system as the eluent (gradient elution up to 50% vol. ethyl acetate) to yield the Boc-protected guanylated alcohol (HO-Et-BocGua) as a white solid (3.2 g, 10.4 mmol, 90% yield). ^1^H-NMR (400 MHz, CDCl_3_, 22 °C): *δ* 11.45 (s, 1H, N*H*), 8.72 (s, 1H, N*H*), 3.77 (dd, *J* = 5.3, 3.9 Hz, 2H, –C*H*_2_–), 3.57 (dd, *J* = 9.2, 5.5 Hz, 2H, –C*H*_2_–), 1.49 (d, *J* = 8.2 Hz, 18H, Boc –C*H*_3_).

*HO-Pr-BocGua*: Yield: 87%; ^1^H-NMR (400 MHz, CDCl_3_, 22 °C): *δ* 11.44 (s, 1H, N*H*), 8.47 (s, 1H, N*H*), 3.57 (dt, *J* = 12.1, 5.8 Hz, 4H, –C*H*_2_–), 1.72–1.66 (m, 2H, –C*H*_2_–), 1.48 (d, *J* = 9.9 Hz, 18H, Boc –C*H*_3_).

*HO-But-BocGua*: Yield: 90%; ^1^H-NMR (400 MHz, CDCl_3_, 22 °C): *δ* 11.48 (s, 1H, N*H*), 8.39 (s, 1H, N*H*), 3.69 (t, *J* = 6.1 Hz, 2H, –C*H*_2_–), 3.49–3.41 (m, 2H, –C*H*_2_–), 1.71–1.59 (m, 4H, –C*H*_2_–), 1.49 (d, *J* = 2.3 Hz, 18H, Boc –C*H*_3_).

*HO-Pen-BocGua*: Yield: 89%; ^1^H-NMR (400 MHz, CDCl_3_, 22 °C): *δ* 11.49 (s, 1H, N*H*), 8.31 (s, 1H, N*H*), 3.64 (t, *J* = 6.5 Hz, 2H, –C*H*_2_–), 3.42 (td, *J* = 7.2, 5.4 Hz, 2H, –C*H*_2_–), 1.64–1.56 (m, 4H, –C*H*_2_–), 1.49 (d, *J* = 3.6 Hz, 18H, Boc –C*H*_3_), 1.43 (ddd, *J* = 12.4, 5.7, 3.0 Hz, 2H, –C*H*_2_–).

*HO-Cy-BocGua*: Yield: 82%; ^1^H-NMR (400 MHz, CDCl_3_, 22 °C): *δ* 11.53 (s, 1H, N*H*), 8.24 (s, 1H, N*H*), 4.08–3.95 (m, 1H, –C*H*), 3.70–3.59 (m, 1H, –C*H*), 2.12–2.04 (m, 2H, –C*H*_2_–), 2.00–1.91 (m, 2H, –C*H*_2_–), 1.49 (d, *J* = 7.7 Hz, 18H, Boc –C*H*_3_), 1.45–1.38 (m, 2H, –C*H*_2_–), 1.32–1.22 (m, 2H, –C*H*_2_–).

*HO-Ph-BocGua*: Yield: 72%; ^1^H-NMR (400 MHz, CDCl_3_, 22 °C): *δ* 11.62 (s, 1H, N*H*), 9.95 (s, 1H, N*H*), 7.08–7.02 (m, 2H, Ph –C*H*), 6.63–6.53 (m, 2H, Ph –C*H*), 1.49 (d, *J* = 34.8 Hz, 18H, Boc –C*H*_3_).

*HO-Bn-BocGua*: Yield: 85%; ^1^H-NMR (400 MHz, CDCl_3_, 22 °C): *δ* 11.53 (s, 1H, N*H*), 8.53 (s, 1H, N*H*), 7.19–7.09 (m, 2H, Ph –C*H*), 6.84–6.73 (m, 2H, Ph –C*H*), 4.52 (d, *J* = 5.1 Hz, 2H, –C*H*_2_–), 1.49 (d, *J* = 12.4 Hz, 18H, Boc –C*H*_3_).

### Synthesis of MTC-OX-BocGua monomers

MTC-OX-BocGua was synthesized with reference to the protocol reported in the previous work^33^ (Figure 1a). Briefly, in a dry three-neck round bottom flask equipped with a stir bar, MTC-OH (3.08 g, 19.3 mmol) was dissolved in dry THF (50 mL) with a 3–4 drops of dimethylformamide (DMF). A solution of oxalyl chloride (2.45 mL, 28.5 mmol) in THF (50 mL) was subsequently added from a dropping funnel. Under an inert atmosphere, the solution was stirred for 1 h, after which volatiles were removed under vacuum, yielding an off-white solid (i.e., 5-chlorocarboxy-5-methyl-1,3-dioxan-2-one intermediate). The solid was heated to 60 °C for a brief 2–3 min to remove any residual solvent, and then re-dissolved in dry CH_2_Cl_2_ (50 mL) and cooled down to 0 °C via an ice bath. A mixture of the relevant Boc-protected guanylated alcohol (e.g., HO-Et-BocGua, 5.4 g, 17.8 mmol) and pyridine (1.55 mL, 19.3 mmol) dissolved in dry CH_2_Cl_2_ (50 mL) was then added dropwise over a duration of 30 min, and allowed to stir at 0 °C for an additional 30 min before leaving it at ambient temperature for further stirring overnight. After removal of solvent, the crude product was subjected to purification by flash column chromatography using silica gel and a hexane-ethyl acetate solvent system as the eluent (gradient elution up to 80% vol. ethyl acetate) to yield MTC-OEt-BocGua as a white solid (78% yield). ^1^H-NMR (400 MHz, CDCl_3_, 22 °C): *δ* 11.48 (s, 1H, N*H*), 8.64 (s, 1H, N*H*), 4.71 (d, *J* = 10.9 Hz, 2H, C*H*_*a*_H_b_), 4.33 (t, *J* = 5.2 Hz, 2H, –OC*H*_2_–), 4.22 (d, *J* = 10.9 Hz, 2H, CH_a_*H*_*b*_), 3.77 (d, *J* = 5.1 Hz, 2H, –C*H*_2_N*–*), 1.49 (d, *J* = 1.8 Hz, 18H, Boc –C*H*_3_), 1.39 (d, *J* = 3.4 Hz, 3H, –C*H*_3_).

*MTC-OPr-BocGua*: Yield: 75%; ^1^H-NMR (400 MHz, CDCl_3_, 22 °C): *δ* 11.50 (s, 1H, N*H*), 8.40 (s, 1H, N*H*), 4.72 (d, *J* = 10.9 Hz, 2H, C*H*_*a*_H_b_), 4.26 (t, *J* = 6.1 Hz, 2H, –OC*H*_2_–), 4.21 (d, *J* = 10.9 Hz, 2H, CH_a_*H*_*b*_), 3.53 (dd, *J* = 12.5, 6.7 Hz, 2H, –C*H*_2_N*–*), 1.99–1.91 (m, 2H, –C*H*_2_–), 1.49 (d, *J* = 1.7 Hz, 18H, Boc –C*H*_3_), 1.37 (s, 3H, –C*H*_3_).

*MTC-OBut-BocGua*: Yield: 75%; ^1^H-NMR (400 MHz, CDCl_3_, 22 °C): *δ* 11.44 (s, 1H, N*H*), 8.30 (t, *J* = 4.6 Hz, 1H, N*H*), 4.64 (d, *J* = 10.8 Hz, 2H, C*H*_*a*_H_b_), 4.20–4.14 (m, 4H, CH_a_*H*_*b*_ and –OC*H*_2_–), 3.40 (dd, *J* = 12.4, 6.9 Hz, 2H, –C*H*_2_N*–*), 1.68 (dd, *J* = 5.3, 3.0 Hz, 2H, –C*H*_2_–), 1.63–1.57 (m, 2H, –C*H*_2_–), 1.44 (s, 18H, Boc –C*H*_3_), 1.28 (s, 3H, –C*H*_3_).

*MTC-OPen-BocGua*: Yield: 79%; ^1^H-NMR (400 MHz, CDCl_3_, 22 °C): *δ* 11.50 (s, 1H, N*H*), 8.33 (s, 1H, N*H*), 4.68 (d, *J* = 10.8 Hz, 2H, C*H*_*a*_H_b_), 4.20 (m, *J* = 11.4, 4.5 Hz, 4H, CH_a_*H*_*b*_ and –OC*H*_2_–), 3.43 (dd, *J* = 12.5, 6.8 Hz, 2H, –C*H*_2_N*–*), 1.70 (dd, *J* = 14.8, 7.0 Hz, 2H, –C*H*_2_–), 1.63–1.58 (m, 2H, –C*H*_2_–), 1.50 (d, *J* = 3.6 Hz, 18H, Boc –C*H*_3_), 1.46–1.38 (m, 2H, –C*H*_2_–), 1.34 (s, 3H, –C*H*_3_).

*MTC-OCy-BocGua*: Yield: 60%; ^1^H-NMR (400 MHz, CDCl_3_, 22 °C): *δ* 11.50 (s, 1H, N*H*), 8.31 (s, 1H, N*H*), 4.88–4.77 (m, 1H, –C*H*), 4.67 (d, *J* = 10.8 Hz, 2H, C*H*_*a*_H_b_), 4.18 (d, *J* = 10.8 Hz, 2H, CH_a_*H*_*b*_), 4.14–4.01 (m, 1H, –C*H*), 2.09 (dd, *J* = 13.2, 3.3 Hz, 2H, –C*H*_2_–), 2.01–1.94 (m, 2H, –C*H*_2_–), 1.62–1.56 (m, 2H, –C*H*_2_–), 1.49 (d, *J* = 6.0 Hz, 18H, Boc –C*H*_3_), 1.42–1.32 (m, 2H, –C*H*_2_–), 1.31 (s, 3H, –C*H*_3_).

*MTC-OPh-BocGua*: Yield: 75%; ^1^H-NMR (400 MHz, CDCl_3_, 22 °C): *δ* 11.62 (s, 1H, N*H*), 10.40 (s, 1H, N*H*), 7.65 (d, *J* = 9.0 Hz, 2H, Ph –C*H*), 7.05 (d, *J* = 9.0 Hz, 2H, Ph –C*H*), 4.84 (d, *J* = 11.0 Hz, 2H, C*H*_*a*_H_b_), 4.31 (d, *J* = 10.9 Hz, 2H, CH_a_*H*_*b*_), 1.52 (d, *J* = 14.3 Hz, 21H, Boc –C*H*_3_ and –C*H*_3_).

*MTC-OBn-BocGua*: Yield: 78%; ^1^H-NMR (400 MHz, CDCl_3_, 22 °C): *δ* 11.53 (s, 1H, N*H*), 8.67 (s, 1H, N*H*), 7.35 (dd, *J* = 6.6, 4.7 Hz, 2H, Ph –C*H*), 7.10–7.04 (m, 2H, Ph –C*H*), 4.84 (d, *J* = 11.0 Hz, 2H, C*H*_*a*_H_b_), 4.66 (d, *J* = 5.2 Hz, 2H, –C*H*_2_–), 4.32 (d, *J* = 10.9 Hz, 2H, CH_a_*H*_*b*_), 1.53–1.47 (m, 21H, Boc –C*H*_3_ and –C*H*_3_).

### Synthesis of P(MTC-OX-BocGua) polymers

The detailed procedures for the ring-opening polymerization (ROP) of MTC-OEt-BocGua with 4-methyl benzyl alcohol as initiator are given as a representative example (Figure 1b). Using a glove box, the 4-methyl benzyl alcohol (6.1 mg, 0.05 mmol) was added to a reaction vial containing TU (18.5 mg, 0.05 mmol) and DBU (7.47 μL, 0.05 mmol) dissolved in dry DCM (1 mL) and left to stir for about 10 min. The mixture was subsequently charged with MTC-OEt-BocGua (445 mg, 1.0 mmol) and left to stir at room temperature for an additional 30 min. The reaction was stopped by quenching the catalyst using an excess of benzoic acid (10 mg, 0.08 mmol). The crude polymer was isolated and purified via preparative size-exclusion chromatography using THF as the eluent. Upon removal of the solvent in vacuo, a transparent white solid was obtained as the product, P(MTC-OEt-BocGua)_**_**_20 (79% yield). ^1^H-NMR (400 MHz, CDCl_3_, 22 °C): *δ* 11.47 (s, 22H, N*H*), 8.61 (bs, 22H, N*H*), 4.41–4.20 (m, 129H, C*H*_*a*_H_b,_ CH_a_*H*_*b*_ and –OC*H*_2_–), 3.79–3.65 (m, –C*H*_2_N*–*, overlapped with residual THF peak), 2.34 (s, 3H, initiator –C*H*_3_), 1.47 (d, *J* = 14.3, 427H, Boc –C*H*_3_), 1.24 (bs, 69H, –C*H*_3_).

*P(MTC-OPr-BocGua)_20*: Yield: 74%; ^1^H-NMR (400 MHz, CDCl_3_, 22 °C): *δ* 11.49 (s, 18H, N*H*), 8.43 (bs, 18H, N*H*), 5.10 (s, 2H, initiator –C*H*_2_–), 4.36–4.25 (m, 69H, C*H*_*a*_H_b_ and CH_a_*H*_*b*_), 4.23–4.17 (m, 39H, –OC*H*_2_–), 3.56–3.46 (m, 37H, –C*H*_2_N*–*), 2.34 (s, 3H, initiator –C*H*_3_), 1.93 (m, 38H, –C*H*_2_–), 1.46 (d, *J* = 23.0 Hz, 386H, Boc –C*H*_3_), 1.24 (bs, 69H, *-*C*H*_3_).

*P(MTC-OBut-BocGua)_20*: Yield: 72%; ^1^H-NMR (400 MHz, CDCl_3_, 22 °C): *δ* 11.50 (s, 18H, N*H*), 8.36 (bs, 18H, N*H*), 5.09 (s, 2H, initiator –C*H*_2_–), 4.29 (m, 66H, C*H*_*a*_H_b_ and CH_a_*H*_*b*_), 4.14 (m, 35H, –OC*H*_2_–), 3.45 (m, 36H, –C*H*_2_N*–*), 2.34 (s, 3H, initiator –C*H*_3_), 1.72–1.63 (m, 71H, –C*H*_2_–), 1.53–1.41 (d, 363H, Boc –C*H*_3_), 1.28–1.18 (bs, 57H, –C*H*_3_).

*P(MTC-OPen-BocGua)_20*: Yield: 70%; ^1^H-NMR (400 MHz, CDCl_3_, 22 °C): *δ* 11.53 (s, 19H. N*H*), 8.41 (bs, 19H, N*H*), 5.12 (s, 2H, initiator –C*H*_2_–), 4.44–4.23 (m, 80H, C*H*_*a*_H_b_ and CH_a_*H*_*b*_), 4.14 (m, 43H, –C*H*_2_–), 3.46 (m, 42H, –C*H*_2_–), 2.36 (s, 3H, initiator –C*H*_3_), 1.66 (m, 101H, –C*H*_2_–), 1.49 (d, *J* = 22.2, 4.7 Hz, 410H, Boc –C*H*_3_), 1.48–1.34 (m, 70H, –C*H*_2_–), 1.26 (bs, 60H, –C*H*_3_).

*P(MTC-OCy-BocGua)_20*: Yield: 71%; ^1^H-NMR (400 MHz, CDCl_3_, 22 °C): *δ* 11.53 (s, 19H, N*H*), 8.35 (bs, 18H, N*H*), 5.12 (s, 2H, initiator –C*H*_2_–), 4.80 (m, 19H, –C*H*), 4.37–4.22 (m, 69H, C*H*_*a*_H_b_ and CH_a_*H*_*b*_), 4.10 (s, 19H, –C*H*_2_–), 2.37 (s, 3H, initiator –C*H*_3_), 2.10 (m, 41H, –C*H*_2_–), 1.96 (m, 40H, –C*H*_2_–), 1.64 (m, 40H, –C*H*_2_–), 1.49 (d, *J* = 13.7 Hz, 416H, Boc -C*H*_3_), 1.38 (m, 43H, –C*H*_2_–), 1.23 (bs, 64H, –C*H*_3_).

*P(MTC-OPh-BocGua)_20*: Yield: 70%; ^1^H-NMR (400 MHz, CDCl_3_, 22 °C): *δ* 11.64 (s, 16H, N*H*), 10.34 (bs, 16H, N*H*), 7.54 (m, 35H, Ph –CH), 7.04–6.97 (m, 36H, Ph –C*H*), 5.12 (s, 2H, initiator –C*H*_2_–), 4.67–4.16 (m, 88H, C*H*_*a*_H_b_ and CH_a_*H*_*b*_), 2.34 (s, 3H, initiator –C*H*_3_), 1.56–1.42 (m, 383H, Boc –C*H*_3_ and –C*H*_3_).

*P(MTC-OBn-BocGua)_20*: Yield: 70%; ^1^H-NMR (400 MHz, CDCl_3_, 22 °C): *δ* 11.54 (s, 17H, N*H*), 8.61 (bs, 17H, N*H*), 7.30 (m, 35H, Ph –C*H*), 7.11–6.98 (m, 47H, Ph –C*H*), 5.12 (s, 2H, initiator –C*H*_2_–), 4.61 (m, 39H, –C*H*_2_–), 4.48 (m, 76H, C*H*_*a*_H_b_ and CH_a_*H*_*b*_), 2.35 (s, 3H, initiator –C*H*_3_), 1.55–1.41 (m, 464H, Boc –C*H*_3_ and –C*H*_3_).

### Synthesis of deprotected P(MTC-OX-BocGua) polymers (P(MTC-OX-Gua))

For the post-polymerization removal of Boc groups, an acid-mediated deprotection strategy was adopted (Figure 1b). Briefly, P(MTC-OEt-BocGua)_20 (150 mg) was dissolved in CH_2_Cl_2_ (9 mL) and trifluoroacetic acid (1 mL). The reaction mixture was sealed and stirred at room temperature for 14–18 h. After the removal of solvent in vacuo, slightly yellow waxy solid was obtained as the deprotected guaninidium-functionalized polymer in quantitative yields. The polymer was subsequently dissolved in water and lyophilized to yield a white transparent solid, pEt_20. Complete deprotection was ascertained by ^1^H-NMR analysis. Yield: 87%; ^1^H-NMR (400 MHz, CD_3_OD, 22 °C): *δ* 4.33 (s, 83H, C*H*_*a*_H_b_ and CH_a_*H*_*b*_), 4.25 (m, 43H, –OC*H*_2_–), 3.56–3.48 (m, 45H, –C*H*_2_N–), 1.24 (bs, *J* = 30.2 Hz, 69H, –C*H*_3_).

*pPr_20*: Yield: 80%; ^1^H-NMR (400 MHz, CD_3_OD, 22 °C): *δ* 4.32 (s, 70H, C*H*_*a*_H_b_ and CH_a_*H*_*b*_), 4.23 (m, 39H, –OC*H*_2_–), 3.28 (m, –C*H*_2_N*–*, overlapped with residual H_2_O peak), 2.01–1.88 (m, 38H, –C*H*_2_–), 1.24 (bs, 61H, –C*H*_3_).

*pBut_20*: Yield: 81%; ^1^H-NMR (400 MHz, CD_3_OD, 22 °C): *δ* 4.30 (m, 61H, C*H*_*a*_H_b_ and CH_a_*H*_*b*_), 4.18 (m, 36H, –OC*H*_2_–), 3.22 (m, 36H, –C*H*_2_N*–*), 1.77–1.64 (m, 69H, –C*H*_2_–), 1.23 (bs, 53H, –C*H*_3_).

*pPen_20*: Yield: 85%; ^1^H-NMR (400 MHz, CD_3_OD, 22 °C): *δ* 4.31 (m, 78H, C*H*_*a*_H_b_ and CH_a_*H*_*b*_), 4.16 (m, 43H, –OC*H*_2_–), 3.20 (m, 42H, –C*H*_2_N–), 1.76–1.60 (m, 86H, –C*H*_2_–), 1.52–1.40 (m, 44H, –C*H*_2_–), 1.23 (bs, 65H, –C*H*_3_).

*pCy_2*0: Yield: 82%; ^1^H-NMR (400 MHz, CD_3_OD, 22 °C): *δ* 4.77 (m, 19H, –C*H*), 4.37–4.19 (m, 70H, C*H*_*a*_H_b_ and CH_a_*H*_*b*_), 3.45 (m, 20H, –C*H*), 2.00 (m, 77H, –C*H*_2_*–*), 1.60–1.40 (m, 80H, –C*H*_2_*–*), 1.21 (bs, 58 H, -C*H*_3_).

*pPh_20*: Yield: 70%; ^1^H-NMR (400 MHz, CD_3_OD, 22 °C): *δ* 7.38–7.04 (m, 71H, Ph –C*H*), 4.59–4.34 (m, 72H, C*H*_*a*_H_b_ and CH_a_*H*_*b*_), 1.46–1.32 (m, 55H, –C*H*_3_).

*pBn_20*: Yield: 84%; ^1^H-NMR (400 MHz, CD_3_OD, 22 °C): *δ* 7.28–7.39 (m, 39H, Ph –C*H*), 7.16–7.03 (m, 40H, Ph –C*H*), 4.60–4.29 (m, 115H, C*H*_*a*_H_b,_ CH_a_*H*_*b*_ and –C*H*_2_–), 1.47–1.30 (m, 60H, –C*H*_3_).

### Octanol–water bilayer partitioning

Dansylated guanidinium (pEt_20)^21^ and ammonium-functionalized polycarbonates were synthesized using a modified dansyl alcohol initiator (Supplementary Figure 6). A stock aqueous solution (in PBS) of the respective polymers was prepared to obtain a final concentration of 50 µM. To a 2-mL Eppendorf tube was added an aqueous solution of the polymer (0.5 mL) and octanol (0.5 mL). A series of sodium laurate (various concentrations of 0.5, 1.0, and 2.0 equiv per cationic group) in octanol solutions was prepared. The bilayer mixture was then vortexed for 30 s, centrifuged (2000 rpm for 1 min) and subsequently photographed under illumination from a UV lamp (wavelength: 365 nm).

### Degradation study of pEt_20

The degradation study of pEt_20 was conducted in PBS (pH 7.4) at 37 °C to mimic the physiological environment. PBS buffer was prepared in D_2_O, which allowed for ^1^H NMR analysis. The samples were taken out at 24, 48, and 72 h for ^1^H NMR analysis.

### Membrane integrity study

The minimum bactericidal concentration (MBC that leads to 99.9% killing after 2-h treatment) of pEt_20 and polymyxin B against *A*. *baumannii* was determined prior to the membrane integrity study. *A*. *baumannii* 10073 were suspended in PBS to a concentration of 2 × 10^9^ CFU/mL. The polymer pEt_20 or polymyxin B was added to the bacteria suspension to a concentration of 0.5 × MBC, 1 × MBC, and 2 × MBC. The untreated bacteria suspension was employed as control. The samples were incubated at 37 °C for 2 h, and were then filtered with a 0.22 μm filter to harvest the supernatant. The supernatant was subsequently measured for its absorbance using the Thermo Scientific NanoDrop 2000 spectrophotometer based on UV absorption at 260 nm. Each assay was performed in triplicates, and the data were normalized against the absorbance of supernatant of the untreated cells in PBS. The experiment was independently repeated three times.

### Drug resistance study and gene sequencing

Drug resistance was induced by repeatedly treating *A*. *baumannii* 10073 with imipenem or pEt_20 at sublethal doses^50^. The MIC of imipenem and pEt_20 against the bacteria at each passage was measured using the broth microdilution method described in Supplementary Methods. *A*. *baumannii* 10073 exposed to sub-MIC concentrations (1/2 of MIC at that particular passage) were allowed to regrow and reach a logarithmic growth phase before being used for MIC measurement of the subsequent passage. Development of drug resistance in *A*. *baumannii* was evaluated over 30 passages by recording changes in the MIC normalized to that of the first passage.

After treatment with pEt_20 or imipenem for 30 passages, the RNA samples of the bacteria at 30 passages and the first passage were extracted, sequenced, and analyzed. Briefly, total RNA was isolated from bacteria using the Trizol (Invitrogen) according to the manufacture’s protocol. The purity of RNA samples was assessed using the ND-1000 Nanodrop. RNA integrity was evaluated using the Agilent 2200 TapeStation (Agilent Technologies, USA) and each sample has the RIN^e^ above 7.0. Then, rRNAs were removed from the total RNA using Epicentre Ribo-Zero rRNA Removal Kit (Illumina, USA) and fragmented to approximately 200 bp. The purified RNAs were subsequently subjected to first-strand and second-strand cDNA synthesis, followed by adapter ligation and enrichment with a low cycle according to the instructions from TruSeq® RNA LT/HT Sample Prep Kit (Illumina, USA). The purified library products were evaluated using the Agilent 2200 TapeStation and Qubit®2.0 (Life Technologies, USA) and then diluted to 10 pM for cluster generation in situ on the HiSeq2500 pair-end flow cell, followed by sequencing (2 × 100 bp) on HiSeq2500.

### RNA-seq data analyses

Paired-end fastq files were mapped using BWA-MEM^51^ to the curated reference MDR strain AB030 [NZ_CP009257.1]. Mapped reads were counted using HTSeq^52^. EdgeR Bioconductor package^53^ was used to assess differential gene expression. All samples in each comparison (imipenem treatment versus control or pEt_20 treatment versus control) were filtered for lowly expressed/low coverage genes, i.e., rows with counts per million values (CPM) less than 1 were removed and the library size was adjusted accordingly before normalization. Differential gene expression was assessed after modeling for variability in the data using moderated tagwise dispersion. *P* values are adjusted for multiple hypothesis testing using Benjamini–Hochberg method and genes with adjusted *p* values <0.05 were considered significant. Significant genes with at least a Log_2_-fold change of 1 are presented in Supplementary Data 1 (imipenem/control) and Supplementary Data 2 (pEt_20/control), respectively.

### In vitro immunogenicity test

Peripheral blood mononuclear cells (PBMCs) were isolated by a standard Ficoll-Hypaque density centrifugation technique (Ficoll-paque Plus, GE Healthcare, Cat #10255485). Briefly, blood of 6–8 weeks old C57BL/6 mice was collected in 1.5 mg/mL EDTA-K2 solution in the presence of the anti-coagulant heparin. The samples were mixed with RPMI1640 medium (1:1). Having added Ficoll-Paque (1:1 ratio), we centrifuged the solution at 500 × *g* for 30 min and carefully aspirated mononuclear cell layer. Cells were washed twice by RPMI1640 and were re-suspended in medium at a density of 4 × 10^6^ cells/mL and cultured in 24-well plates (2 × 10^6^ cells per well) in 5% CO_2_ incubator at 37 °C for 4 h. Then, cells were gently washed for three times by RPMI1640 medium to remove non-adherent cells. PMBCs were numerated and recultured at 3 × 10 ^5^/well in 96-well plate and treated with pEt_10 and pEt_20 (100 μg/mL) as well as LPS (100 ng/mL). After 2 days of treatment, cell supernatants were collected to measure IFN-γ and TNF-α by ELISA (Invitrogen, Cat #4337757 and Cat #4341109).

### Animals

ICR mice (female, 7 weeks old) and C57BL/6J mice (6–8 weeks old) were used in the in vivo studies. Immunosuppression was induced by intraperitoneal injection of 200 mg cyclophosphamide per kg of body weight 4 days before the injection of bacteria. The animal study protocols were approved by the Institutional Animal Care and Use Committee at the University of North Dakota.

### Evaluation of LD_50_ and LD_5_

The ICR mice were randomly divided into five groups (six per group). Each of the mice was injected intraperitoneally (i.p.) with pEt_10 or pEt_20 at different doses (i.e., 42.0, 43.0, 44.0, 45.0, and 46.0 mg/kg, 0.2 mL/20 g). LD_50_ and LD_5_ values were estimated from the survival rate of treated mice over 7 days using the BLISS method^54^.

### Evaluation of ED_50_ and ED_95_

The ED_50_ and ED_95_ values of pEt_10 and pEt_20 were evaluated in MDR *A*. *baumannii* 10073-, *E*. *coli* 56809-, *K*. *pneumoniae* 8637-, and MRSA 25312-induced peritonitis mouse models (ICR mice). Overnight cultures of *A*. *baumannii* 10073, *E*. *coli* 56809, *K*. *pneumoniae* 8637, and MRSA 25312 were suspended in PBS. The cyclophosphamide-pretreated mice were injected intraperitoneally (i.p.) with bacterial suspension at various doses (*A*. *baumannii* 10073: 8.0 × 10^7^, 1.6 × 10^8^, 2.4 × 10^8^, 3.2 × 10^8^ and 4.0 × 10^8^ CFU/mL; *E*. *coli* 56809: 2.0 × 10^6^, 4.0 × 10^6^, 6.0 × 10^6^, 8.0 × 10^6^, and 1.0 × 10^7^ CFU/mL; *K*. *pneumoniae* 8637: 6.7 × 10^7^, 1.0 × 10^8^, 1.5 × 10^8^, 2.3 × 10^8^ and 3.5 × 10^8^ CFU/mL; MRSA 25312: 1.0 × 10^8^, 2.0 × 10^8^, 4.0 × 10^8^ and 6.0 × 10^8^ and 8.0 × 10^8^ CFU/mL, 0.5 mL via i.p. injection; Six to eight mice per group). The minimum lethal dose, which was enough to cause 100% mortality, was determined based on the survival rate of infected mice at 48 h post infection using the BLISS method^54^.

To evaluate ED_50_ and ED_95_ of the polymers, the immunosuppressed mice were infected with *A*. *baumannii* 10073, *E*. *coli* 56809, *K*. *pneumoniae* 8637, or MRSA 25312 by injecting the corresponding bacterial suspension at the minimum lethal dose to mice intraperitoneally (*A*. *baumannii* 10073: 2.7 × 10^8^ CFU/mL; *E*. *coli* 56809: 8.8 × 10^6^ CFU/mL; *K*. *pneumoniae* 8637: 2.6 × 10^8^ CFU/mL; MRSA 25312: 5.2 × 10^8^ CFU/mL; 0.5 mL per injection). The mice were divided into four groups (six to eight mice per group), and injected with PBS, pEt_10, and pEt_20 and imipenem i.p. at 1 and 6 h or 3 and 8 h post infection at various doses (*A*. *baumannii* 10073 infection: 0.02, 0.05, 0.1, 0.2, and 0.5 mg/kg for the polymers; 0.1, 0.2, 0.5, 1.0, and 2.0 mg/kg for imipenem. *E*. *coli* 56809 infection: 0.1, 0.2, 0.5, 1.0, and 2.0 mg/kg for the polymers and imipenem. *K*. *pneumoniae* 8637 infection: 0.5, 1.0, 2.0, 4.0, and 8.0 mg/kg for the polymers; 2.0, 4.0, 8.0, 16.0, and 32.0 mg/kg for imipenem. MRSA 25312: 0.5, 1.0, 2.0, 4.0, and 8.0 mg/kg for the polymers and vancomycin. 0.2 mL/20 g per injection). The BLISS method was employed to estimate ED_50_ and ED_95_ based on the survival rate of the infected mice over 2 days^54^.

### Bacteria count in the blood, abdominal cavity, and organs after polymer treatment

The immunosuppressed ICR mice were injected i.p. with 0.5 mL of *A*. *baumannii* 10073 or *E*. *coli* 56809 suspension at 2.0 × 10^8^ and 8.0 × 10^6^ CFU/mL, respectively. The mice were then divided into four groups, and injected with PBS, pEt_10, pEt_20, and imipenem at doses that saved 95% mice (ED_95_) (*A*. *baumannii* 10073 infection: 0.28, 0.15, and 2.7 mg/kg, respectively; *E*. *coli* 56809 infection: 3.3, 1.8, and 1.1 mg/kg, respectively) at 1 and 6 h post infection. At 24 h post infection, five mice in each group were killed to obtain blood, peritoneal fluid, and organ samples. For taking peritoneal fluid sample, 3.0 mL of PBS was injected into the peritoneal cavity, and the abdomen area was then gently massaged. Peritoneal fluid (2.0 mL per mouse) was recovered from the peritoneum of each mouse after the abdomen was opened. Blood and peritoneal fluid samples were diluted and plated on MH agar plates. At the same time, liver, spleen, and kidneys were removed and homogenized in 2.0 mL of PBS. The homogenate was diluted and plated on MH agar plates. After overnight incubation at 37 °C, the number of bacterial colonies was counted. For the organs, the data are presented as lg (CFU/mL of homogenate).

### CLP-induced polymicrobial peritonitis mouse model

C57BL/6J mice (male and female, 6–8 weeks, 18–22 g, Jackson Laboratory) were grouped randomly (WT-without surgery or any treatment, Sham-with surgery but without CLP, CLP, CLP-pEt_20, CLP-gentamicin, 6 mice in each group). Mice were fed with normal diet for 12 h before surgery. Then mice were anesthetized with intramuscular (i.m.) injection of ketamine (40 mg/kg of body weight), and the abdominal area was disinfected. The cecum was exposed, ligatured at its external third, and punctured with 27-gauge needle. The abdominal musculature and abdominal skin were closed by applying simple suture. NaCl solution (0.9%, 1 mL) was injected i.p. to supplement the lost moisture. After surgery, the mice were allowed to drink freely. In the Sham group, the cecum was only exposed but not punctured, and was then returned to the abdominal cavity. At 1 h post-CLP (all mice were able to move normally), mice were injected i.p. with pEt_20 and gentamicin solution at 25 and 10 mg/kg, respectively. Another dose was given at 6 h post-CLP. The mice were observed for survival using Kaplan–Meier curves [*n* = 6, *p* = 0.0112 (<0.05), Log-rank test].

In another experiment (three mice in each group), mice were killed at 24 h post-CLP. Blood and peritoneal fluid were collected for analysis of bacterial load.

### Lung infection model

C57BL/6J mice (male and female, 6–8 weeks, 18–22 g, Jackson Laboratory) were grouped randomly (control without infection or any treatment, infection without any treatment, pEt_20 treated and imipenem treated, 6 mice in each group). Lung infection was established by intranasal instillation of *P*. *aeruginosa* PA14 strain [a clinically isolated sample, kindly provided by George A O’Toole (Dartmouth Medical School); 1 × 10^7^ CFU]. The infected mice were treated with pEt_10 and imipenem at 1, 6, and 25 h post infection (8 mg/kg of mouse body weight per injection for pEt_10 and 15 mg/kg of mouse body weight per injection for imipenem). The mice were monitored for survival using Kaplan–Meier curves [*n* = 6*, p* = 0.0214 (<0.05), Log-rank test].

In another experiment (three mice in each group), mice were killed at 72 h post infection. Blood and the lung were collected for analysis of bacterial burden.

### Biodistribution of pEt_20

The experiments were conducted in accordance with the approved protocol from the IACUC at the Biological Resource Centre of Singapore. Balb/c mice, with an average weight of 20 g, were used for this study. The mice were divided into two groups and administrated with 1.3 mg/kg AF750-conjugated polymer (synthesis in Supplementary Information) (in 200 µL of sterile PBS) by i.v. or i.p. injection. The mice were killed at 4, 24 and 48 h post administration and organs including the brain, heart, liver, spleen, lungs, and kidneys were excised and imaged using IVIS (Caliper Life Science, USA). The near-infrared fluorescence was imaged using the ICG filter pairs and exposure time was set to 3 s.

### Pharmacokinetics of pEt_20

The experiments were conducted in accordance with the approved protocol from the Institutional Animal Care and Use Committee (IACUC) at the Biological Resource Centre of Singapore. Balb/c mice (*n* = 5) with an average weight of 20 g was injected with the AF750-conjugated pEt_20 solution (1.3 mg/kg in 200 µL of sterile PBS) as a single bolus into the tail vein. Blood was collected prior to administration of pEt_20 and at 2, 10, 15, 30, 60, 120, 240, and 480 min thereafter, and plasma was harvested by centrifugation at 1000 × *g*. Plasma samples were stored at −80 °C until analysis. The concentrations of AF750-conjugated pEt_20 in the samples were determined by measuring the fluorescence intensities at Ex/Em 749 nm/775 nm.

### Statistical analysis

Analyses for difference between the treatment and control arms were performed using one-way analysis of variance (ANOVA) and post hoc Tukey’s test. *P* values of <0.05 were considered significant. Statistical calculations were performed using SPSS software.

### Data availability

Data supporting the findings of this study are available within the article (and its Supplementary information files).

## Electronic supplementary material

Supplementary Information

Description of Additional Supplementary Files

Supplementary Data 1

Supplementary Data 2
