# Supplementary material for: A macromolecular approach to eradicate multidrug resistant bacterial infections while mitigating drug resistance onset
Source: Nat Commun. 2018 Mar 2;9:917. doi: 10.1038/s41467-018-03325-6 (PMC5834525; doi:10.1038/s41467-018-03325-6)
Supplement: Supplementary file 1 — Supplementary Information [file 41467_2018_3325_MOESM1_ESM.pdf]

Supplementary Information

A macromolecular approach to eradicate multidrug resistant bacterial infections while mitigating drug resistance onset

By Chin et al.

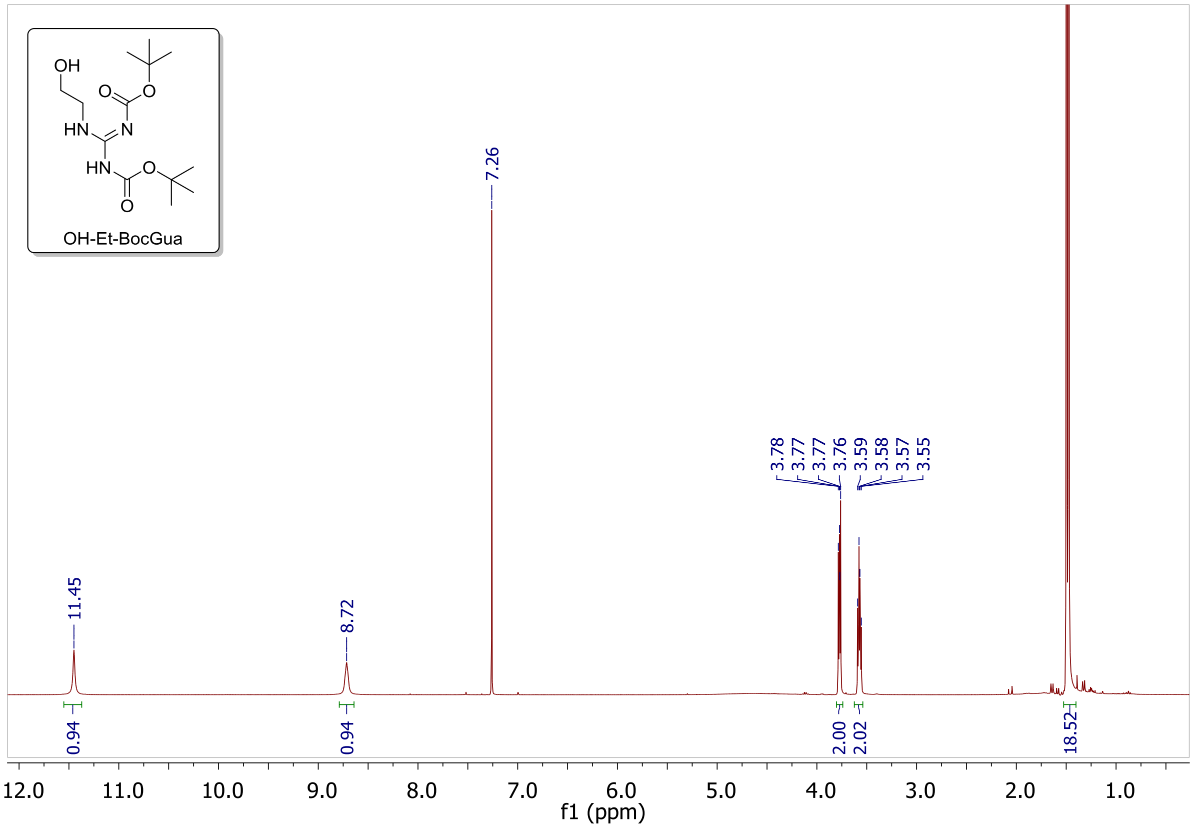
OH-Et-BocGua
11.45
8.72
7.26
3.78
3.77
3.77
3.76
3.59
3.58
3.57
3.55
0.94
0.94
2.00
2.02
18.52
12.0 11.0 10.0 9.0 8.0 7.0 6.0 5.0 4.0 3.0 2.0 1.0
f1 (ppm)

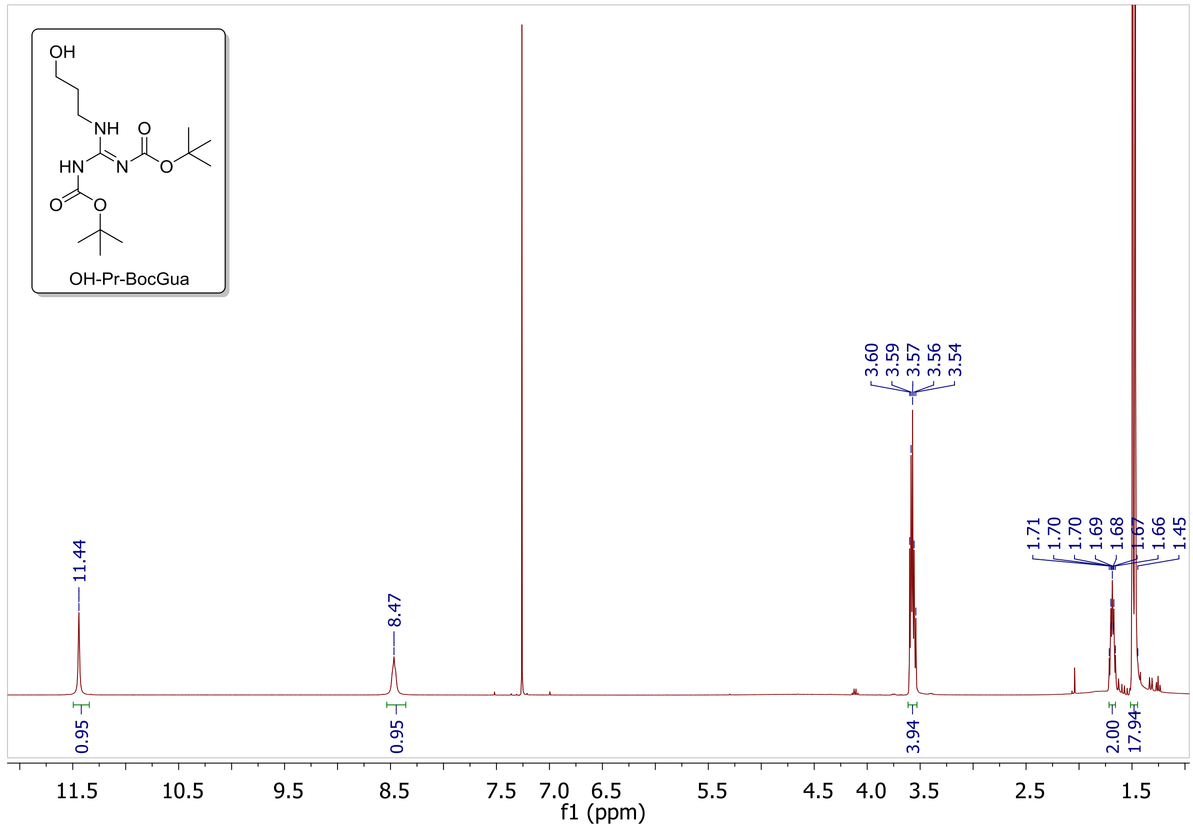
OH-Pr-BocGua
11.44
8.47
3.60
3.59
3.57
3.56
3.54
1.71
1.70
1.70
1.69
1.68
1.67
1.66
1.45
0.95
0.95
3.94
2.00
17.94
11.5 10.5 9.5 8.5 7.5 7.0 6.5 5.5 4.5 4.0 3.5 2.5 1.5
f1 (ppm)

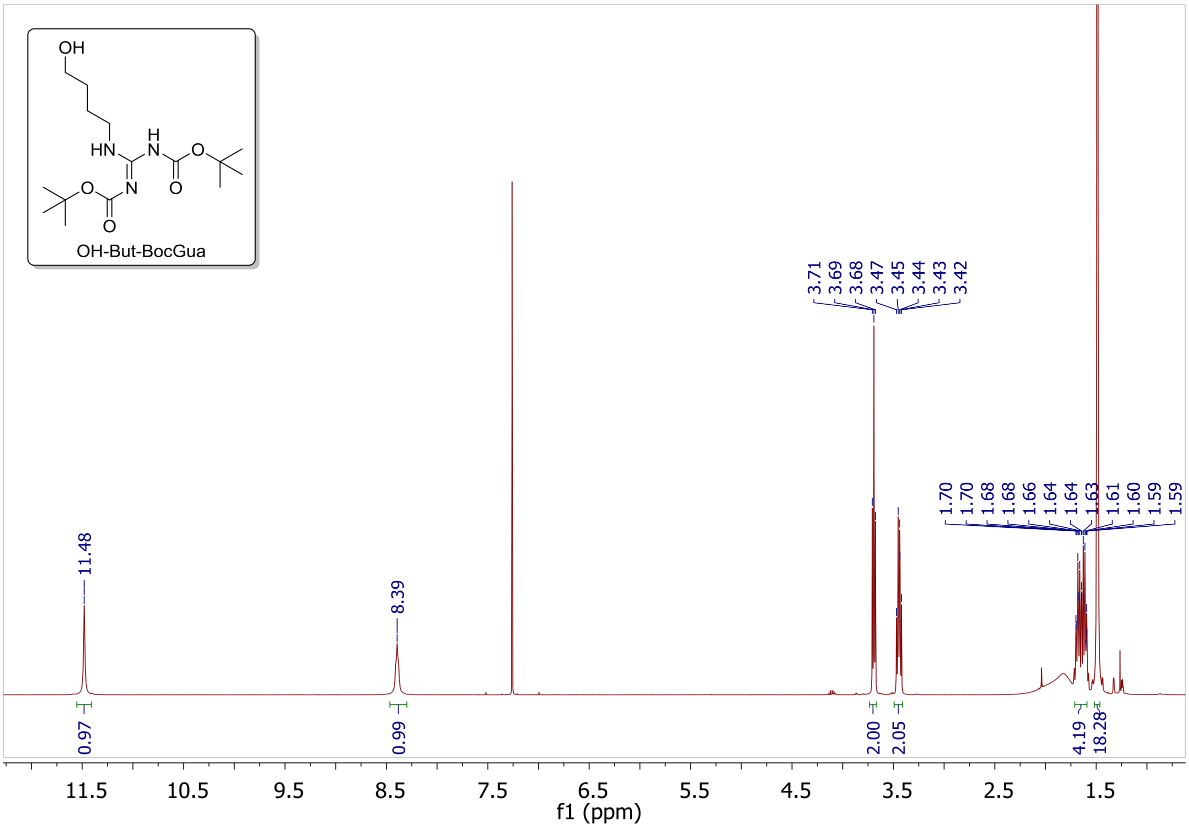
OH-But-BocGua
11.48
8.39
3.71
3.69
3.68
3.47
3.45
3.44
3.43
3.42
1.70
1.70
1.68
1.68
1.66
1.64
1.64
1.63
1.61
1.60
1.59
1.59
0.97
0.99
2.00
2.05
4.19
18.28
f1 (ppm)

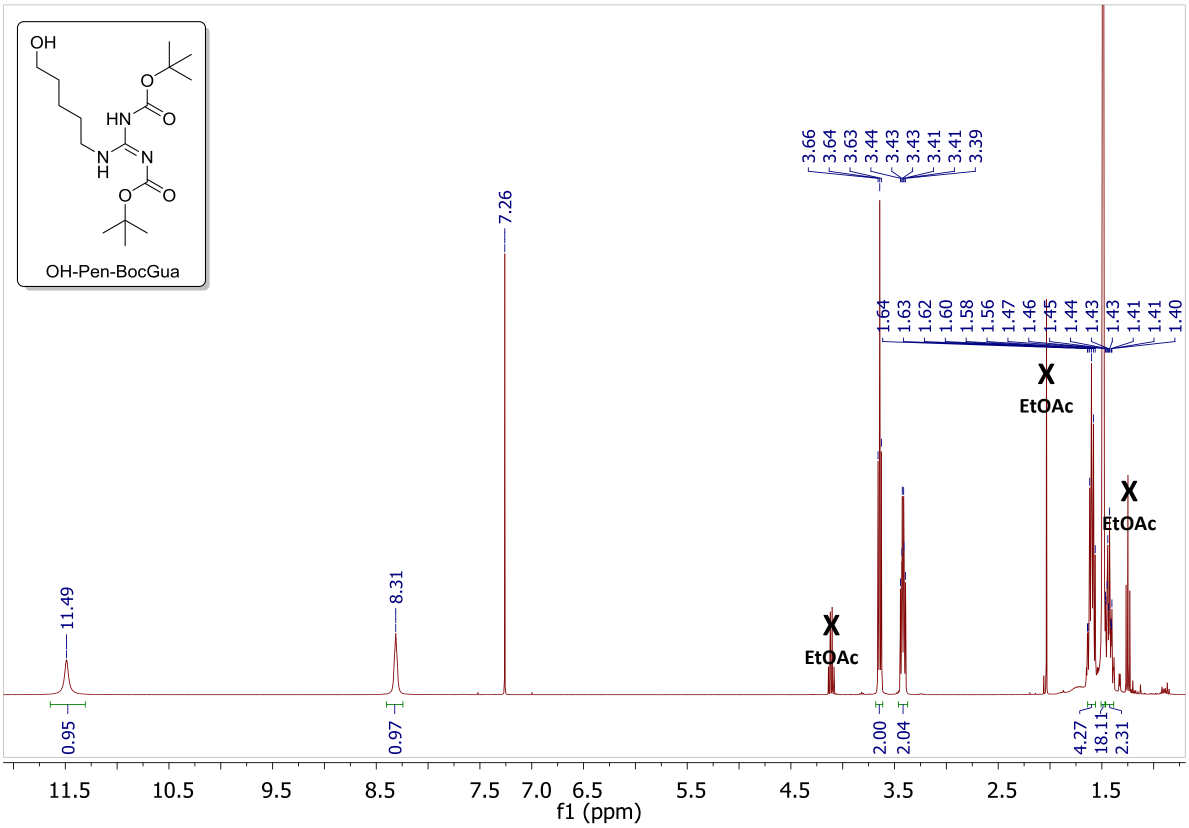
OH-Pen-BocGua
11.49
8.31
7.26
3.66
3.64
3.63
3.44
3.43
3.43
3.41
3.41
3.39
1.64
1.63
1.62
1.60
1.58
1.56
1.47
1.46
1.45
1.44
1.43
1.43
1.41
1.41
1.40
EtOAc
EtOAc
EtOAc
0.95
0.97
2.00
2.04
4.27
18.11
2.31
f1 (ppm)

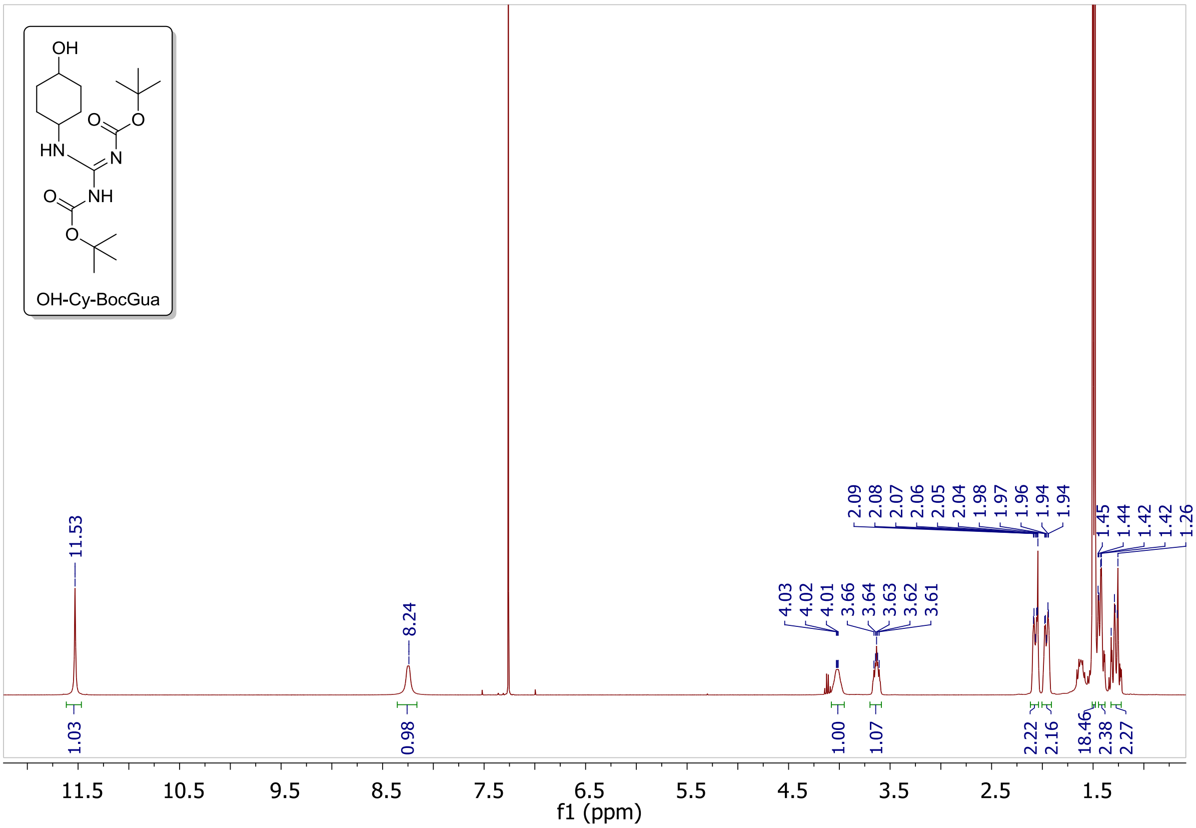
OH
HN
O
O
N
O
NH
O
OH-Cy-BocGua
11.53
8.24
4.03
4.02
4.01
3.66
3.64
3.63
3.62
3.61
2.09
2.08
2.07
2.06
2.05
2.04
1.98
1.97
1.96
1.94
1.94
1.45
1.44
1.42
1.42
1.26
1.03
0.98
1.00
1.07
2.22
2.16
18.46
2.38
2.27
11.5
10.5
9.5
8.5
7.5
6.5
5.5
4.5
3.5
2.5
1.5
f1 (ppm)

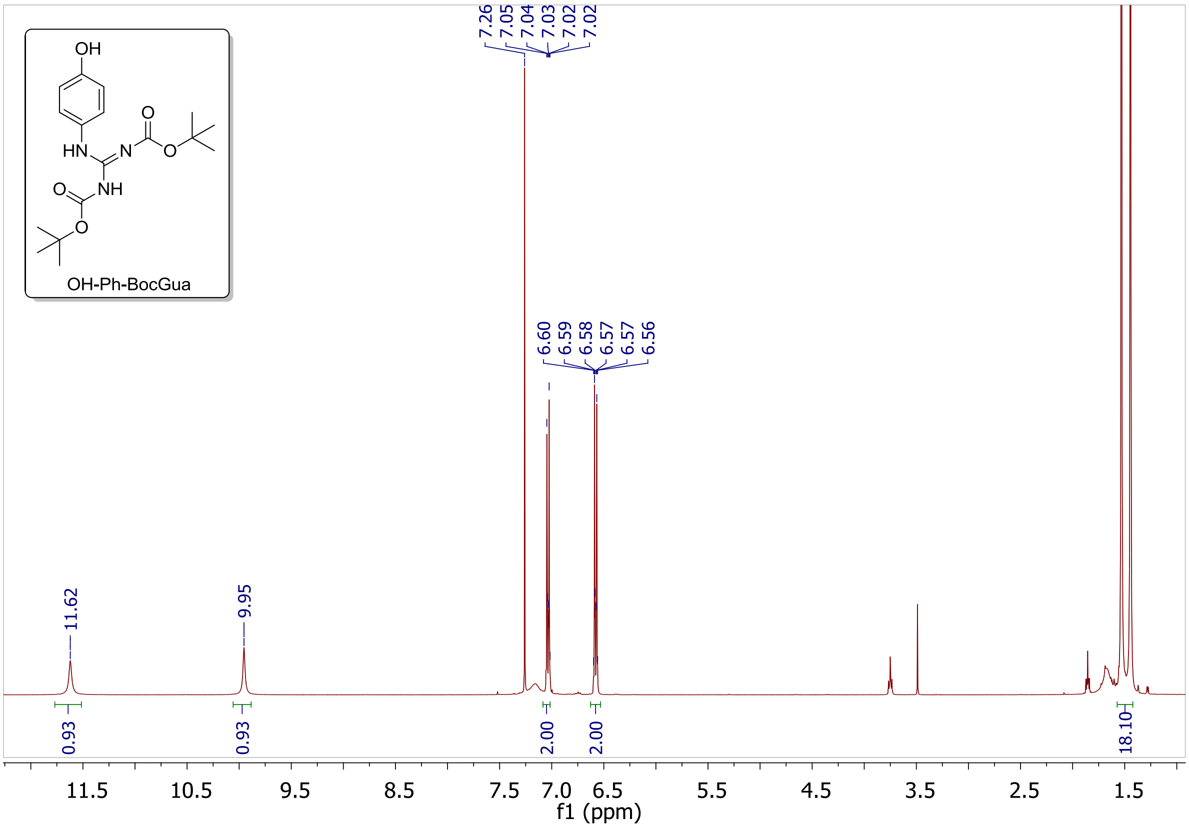
OH-Ph-BocGua
7.26
7.05
7.04
7.03
7.02
7.02
6.60
6.59
6.58
6.57
6.57
6.56
11.62
9.95
0.93
0.93
2.00
2.00
18.10
11.5 10.5 9.5 8.5 7.5 7.0 6.5 5.5 4.5 3.5 2.5 1.5
f1 (ppm)

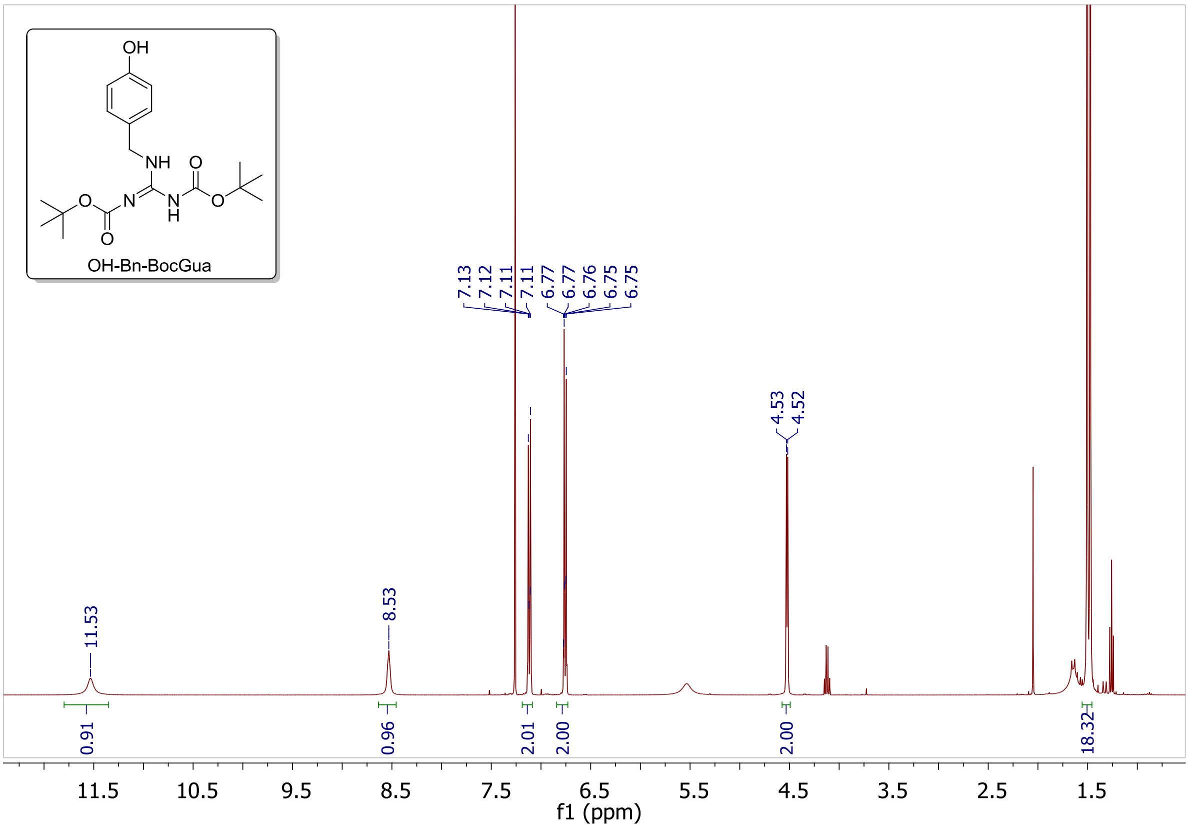
OH-Bn-BocGua
7.13
7.12
7.11
7.11
6.77
6.77
6.76
6.75
6.75
4.53
4.52
11.53
8.53
0.91
0.96
2.01
2.00
2.00
18.32
11.5 10.5 9.5 8.5 7.5 6.5 5.5 4.5 3.5 2.5 1.5
f1 (ppm)

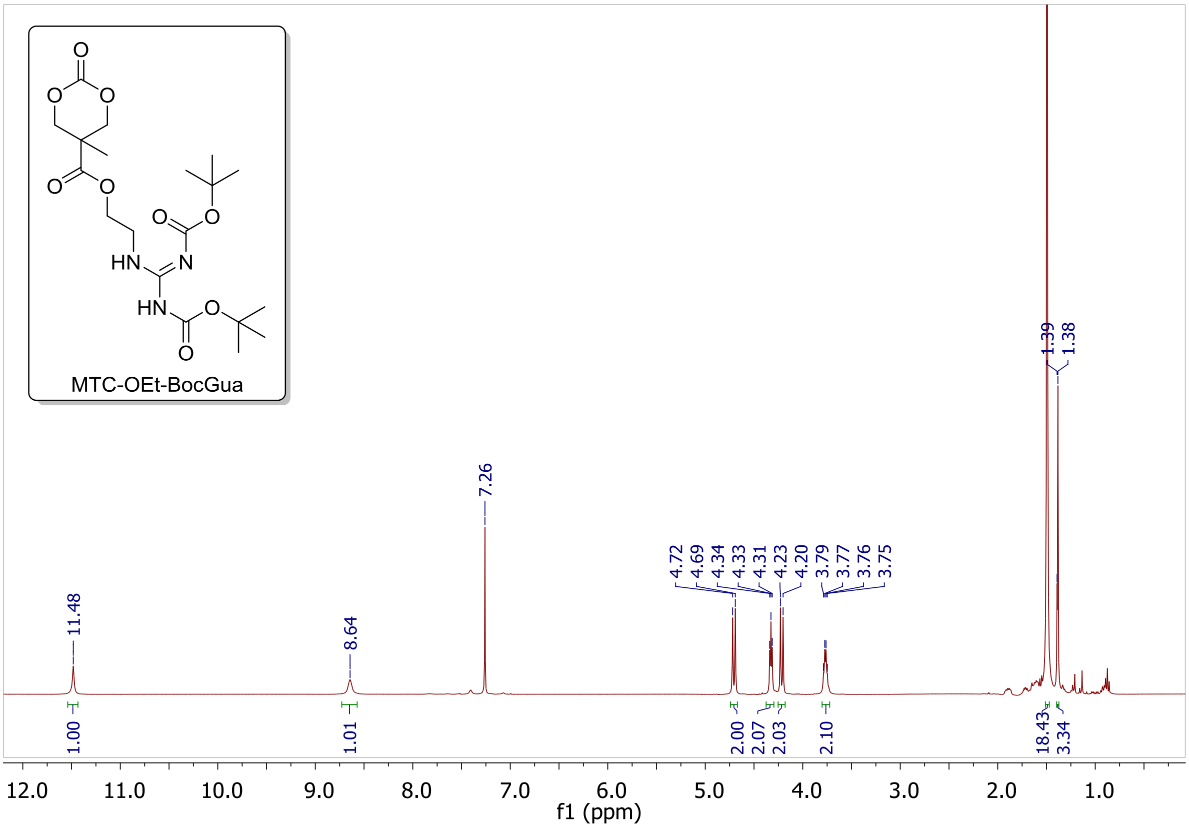
MTC-OEt-BocGua
11.48
8.64
7.26
4.72
4.69
4.34
4.33
4.31
4.23
4.20
3.79
3.77
3.76
3.75
1.39
1.38
1.00
1.01
2.00
2.07
2.03
2.10
18.43
3.34
f1 (ppm)

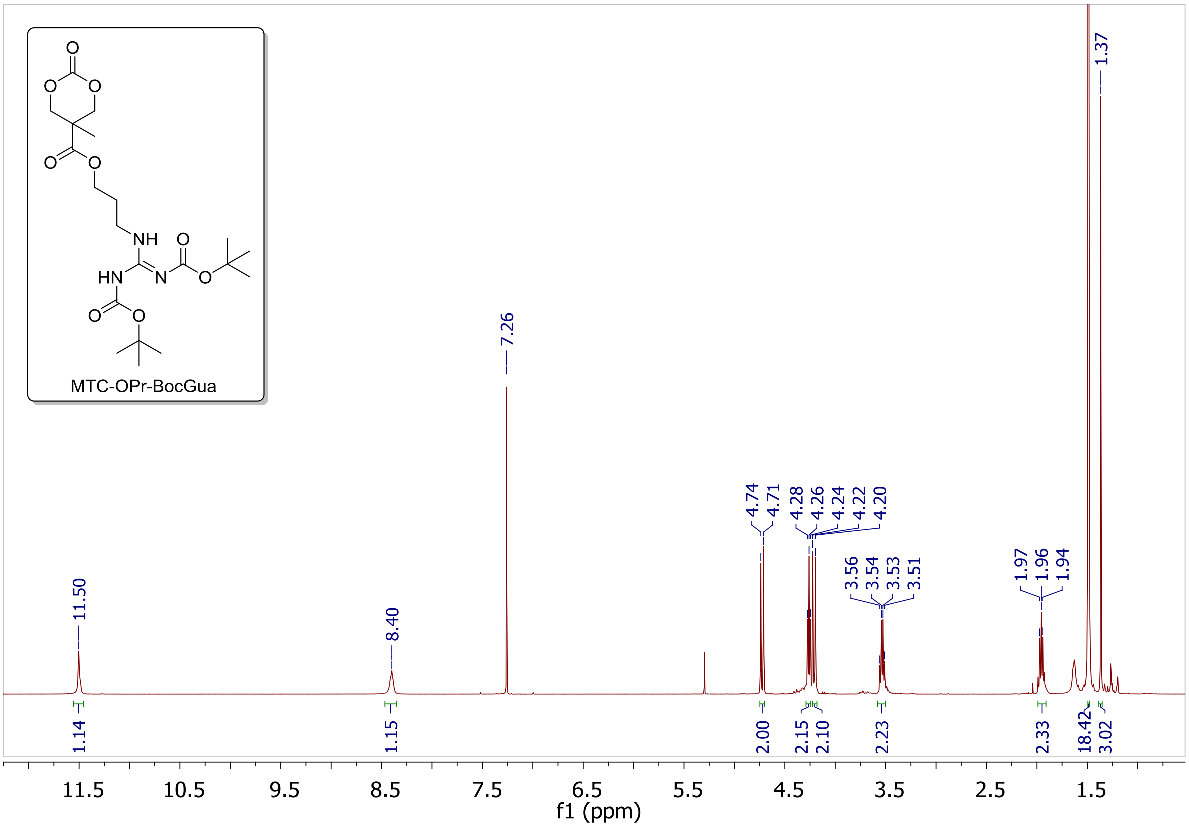
MTC-OPr-BocGua
11.50
8.40
7.26
4.74
4.71
4.28
4.26
4.24
4.22
4.20
3.56
3.54
3.53
3.51
1.97
1.96
1.94
1.37
1.14
1.15
2.00
2.15
2.10
2.23
2.33
18.42
3.02
f1 (ppm)

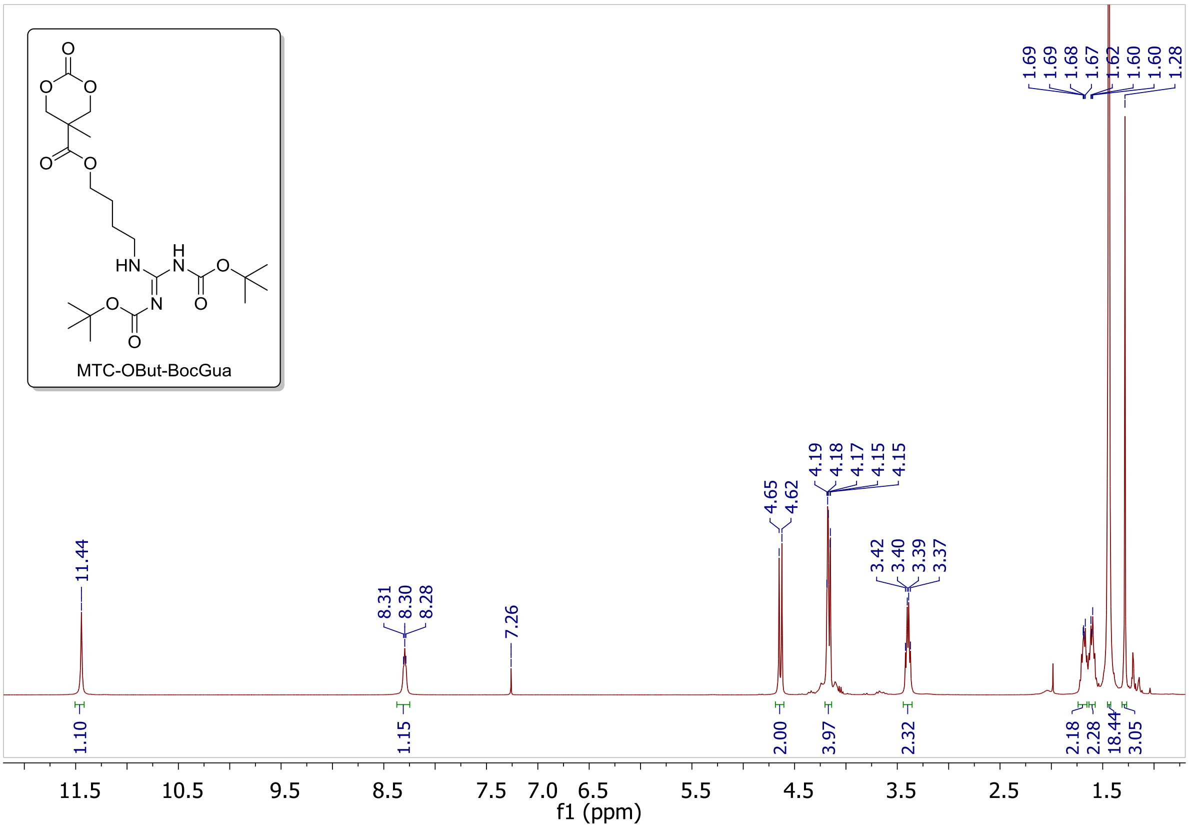
MTC-OBut-BocGua
f1 (ppm)
11.5
10.5
9.5
8.5
7.5
7.0
6.5
5.5
4.5
3.5
2.5
1.5

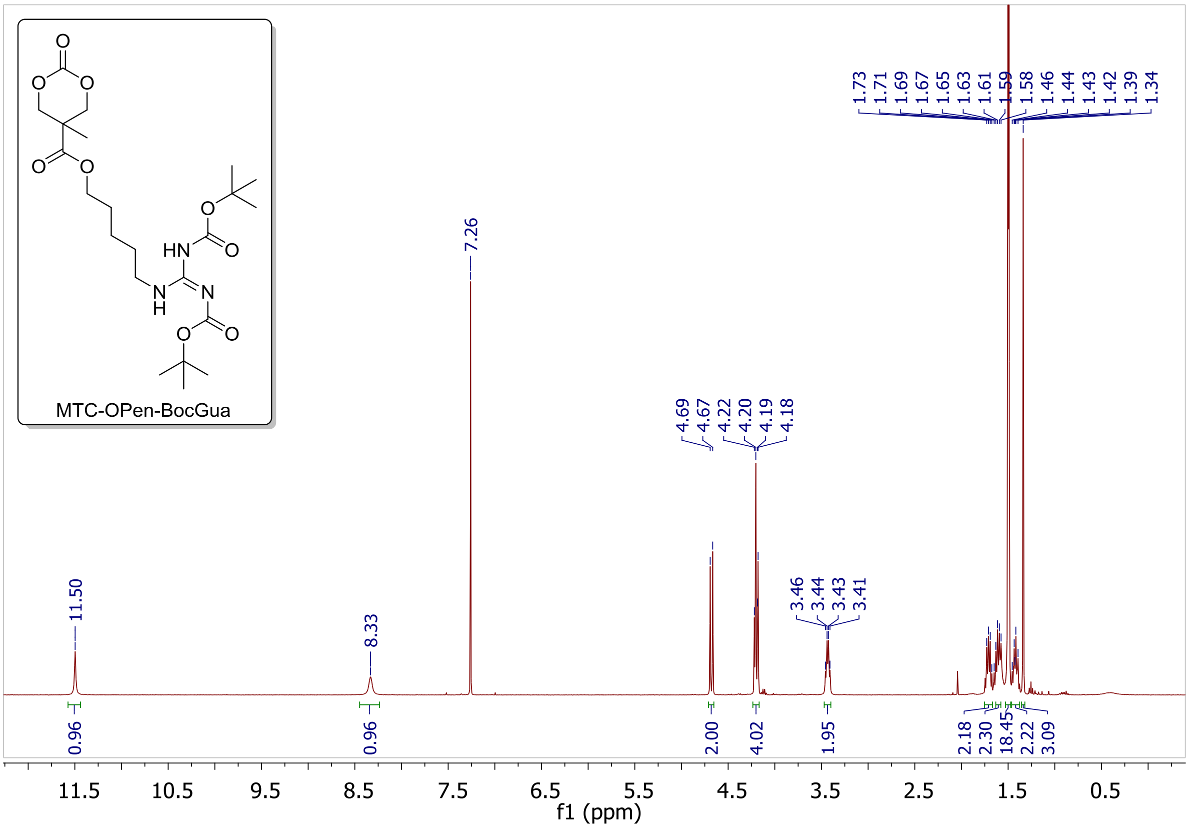
MTC-OPen-BocGua
f1 (ppm)
11.5
10.5
9.5
8.5
7.5
6.5
5.5
4.5
3.5
2.5
1.5
0.5

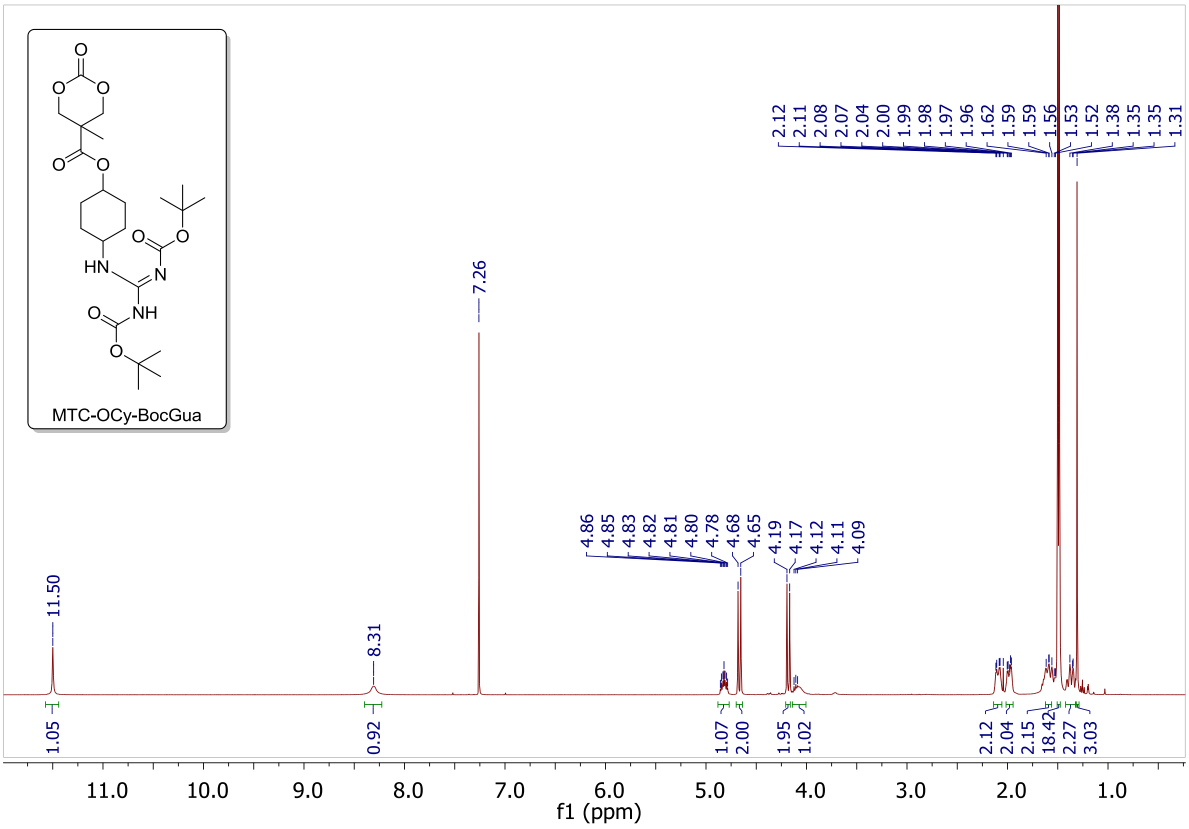

MTC-OCy-BocGua
11.50
8.31
7.26
4.86
4.85
4.83
4.82
4.81
4.80
4.78
4.68
4.65
4.19
4.17
4.12
4.11
4.09
2.12
2.11
2.08
2.07
2.04
2.00
1.99
1.98
1.97
1.96
1.62
1.59
1.56
1.53
1.52
1.38
1.35
1.31
1.05
0.92
1.07
2.00
1.95
1.02
2.12
2.04
2.15
18.42
2.27
3.03
11.0
10.0
9.0
8.0
7.0
6.0
5.0
4.0
3.0
2.0
1.0
f1 (ppm)

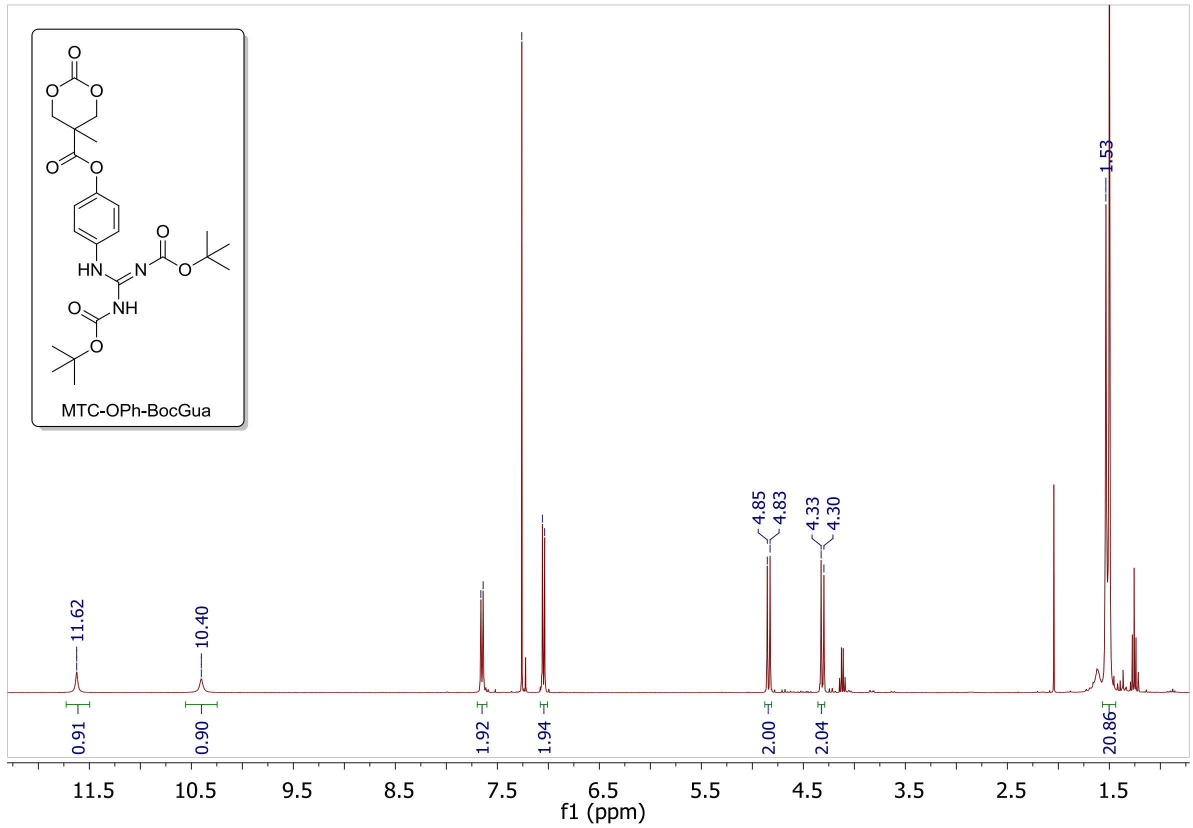
MTC-OPh-BocGua
11.62
10.40
4.85
4.83
4.33
4.30
1.53
0.91
0.90
1.92
1.94
2.00
2.04
20.86
11.5 10.5 9.5 8.5 7.5 6.5 5.5 4.5 3.5 2.5 1.5
f1 (ppm)

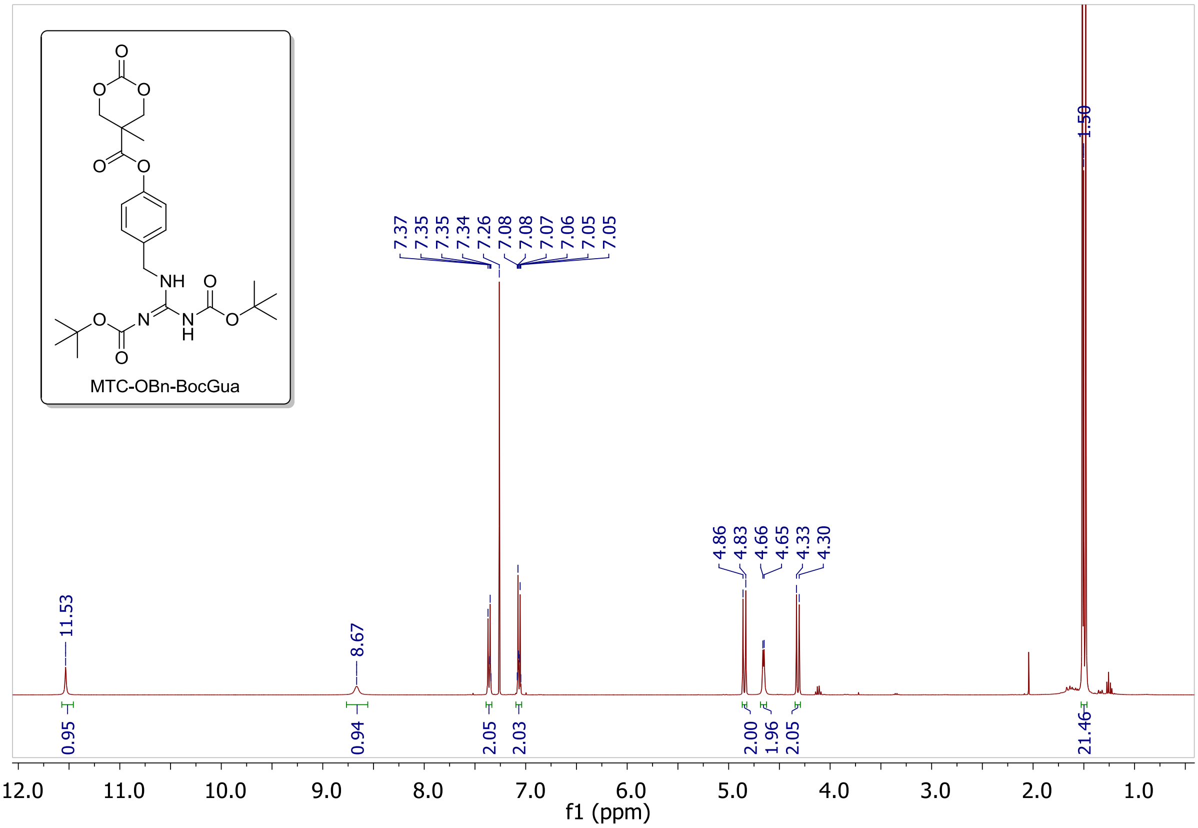
MTC-OBn-BocGua
7.37
7.35
7.35
7.34
7.26
7.08
7.08
7.07
7.06
7.05
7.05
11.53
8.67
4.86
4.83
4.66
4.65
4.33
4.30
1.50
0.95
0.94
2.05
2.03
2.00
1.96
2.05
21.46
12.0 11.0 10.0 9.0 8.0 7.0 6.0 5.0 4.0 3.0 2.0 1.0
f1 (ppm)

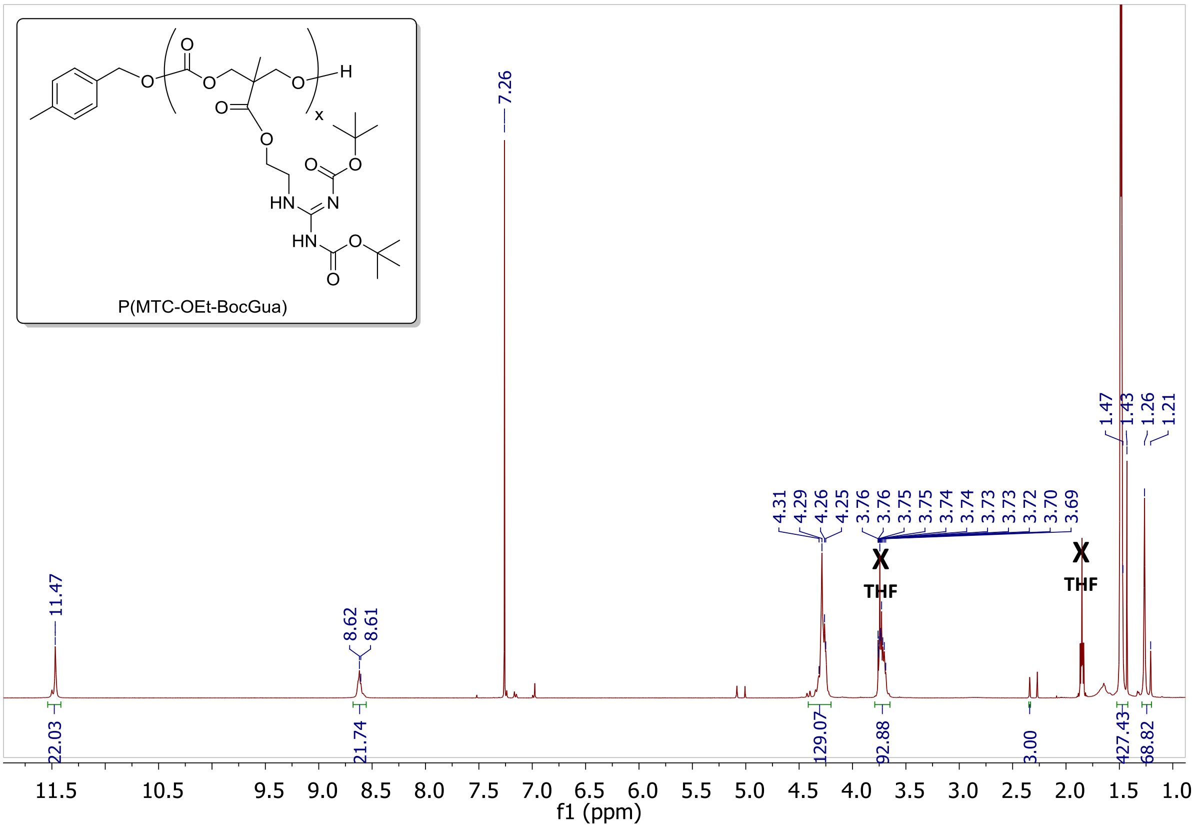
P(MTC-OEt-BocGua)
THF
f1 (ppm)

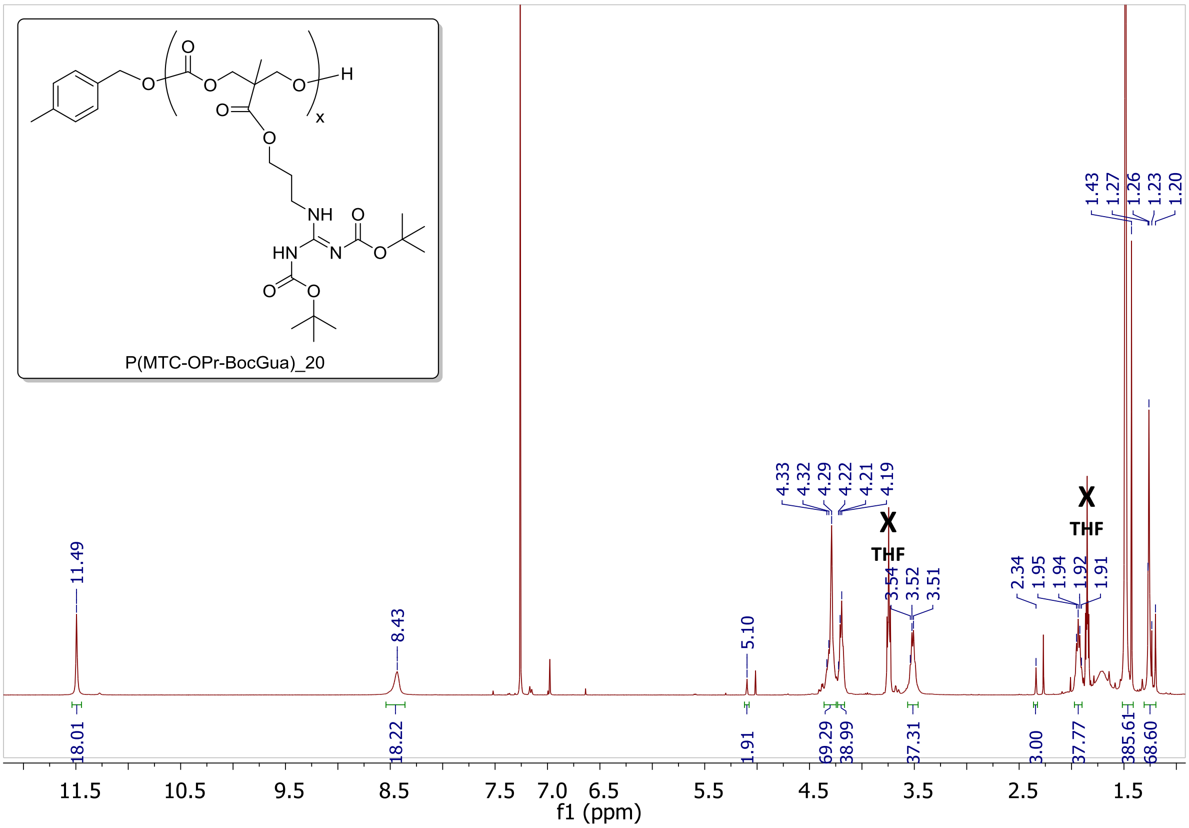
P(MTC-OPr-BocGua)_20
THF
f1 (ppm)

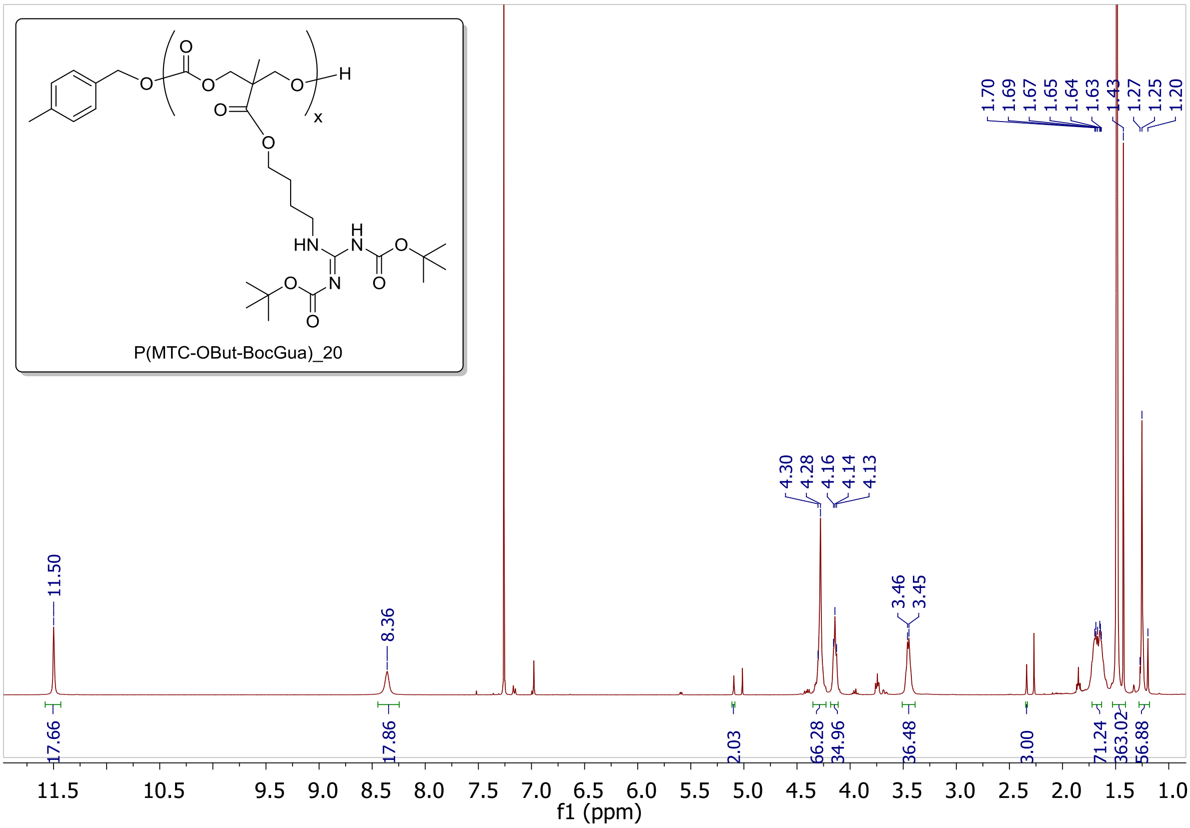
P(MTC-OBut-BocGua)_20
x
H
HN
N
O
1.70 1.69 1.67 1.65 1.64 1.63 1.43 1.27 1.25 1.20
4.30 4.28 4.16 4.14 4.13
3.46 3.45
11.50
8.36
17.66 17.86 2.03 66.28 34.96 36.48 3.00 71.24 363.02 56.88
11.5 10.5 9.5 9.0 8.5 8.0 7.5 7.0 6.5 6.0 5.5 5.0 4.5 4.0 3.5 3.0 2.5 2.0 1.5 1.0
f1 (ppm)

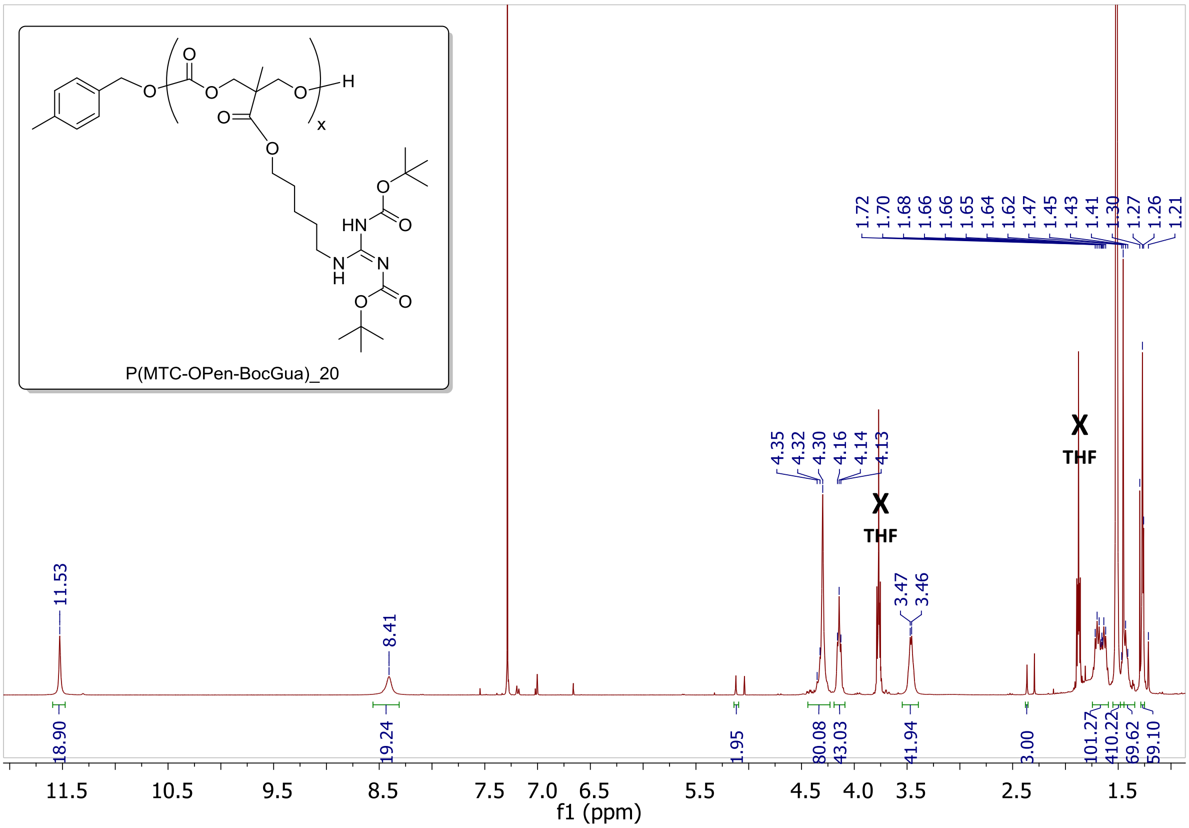
P(MTC-OPen-BocGua)_20
x
H
HN
N
O
1.72 1.70 1.68 1.66 1.66 1.65 1.64 1.62 1.47 1.45 1.43 1.41 1.30 1.27 1.26 1.21
4.35 4.32 4.30 4.16 4.14 4.13
X
THF
X
THF
3.47 3.46
11.53
8.41
18.90 19.24 1.95 80.08 43.03 41.94 3.00 101.27 410.22 69.62 59.10
11.5 10.5 9.5 8.5 7.5 7.0 6.5 5.5 4.5 4.0 3.5 2.5 1.5
f1 (ppm)

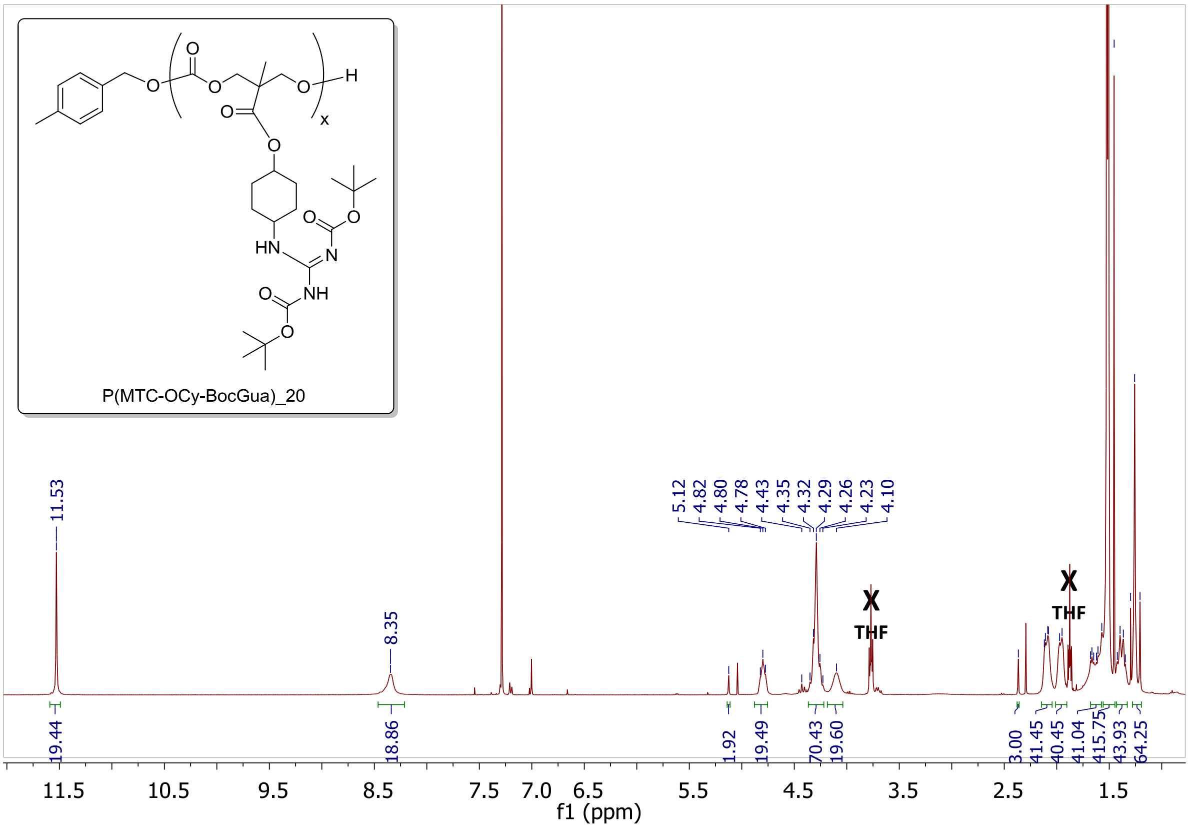
P(MTC-OCy-BocGua)_20
11.53
8.35
5.12 4.82 4.80 4.78 4.43 4.35 4.32 4.29 4.26 4.23 4.10
THF
THF
19.44
18.86
1.92
19.49
70.43
19.60
3.00
41.45
40.45
41.04
415.75
43.93
64.25
f1 (ppm)

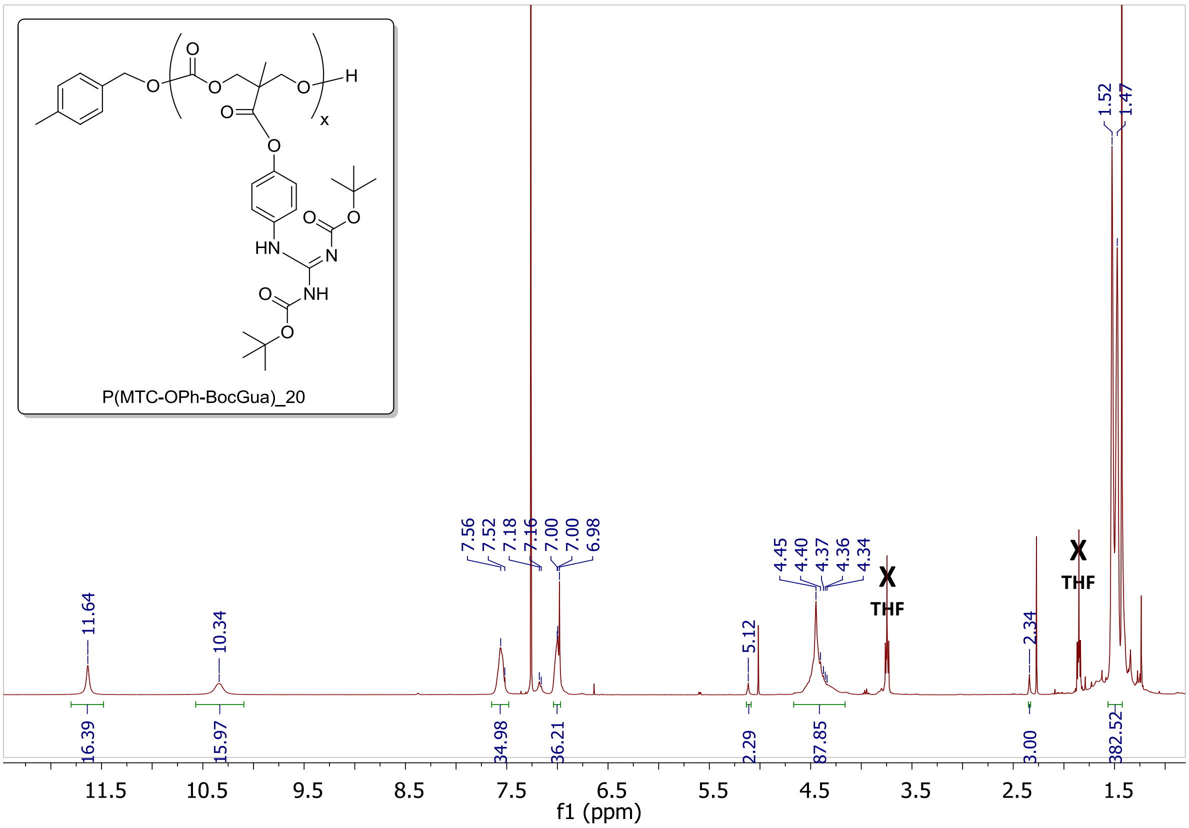
P(MTC-OPh-BocGua)_20
11.64
10.34
7.56
7.52
7.18
7.16
7.00
7.00
6.98
5.12
4.45
4.40
4.37
4.36
4.34
X
THF
2.34
1.52
1.47
16.39
15.97
34.98
36.21
2.29
87.85
3.00
382.52
11.5
10.5
9.5
8.5
7.5
6.5
5.5
4.5
3.5
2.5
1.5
f1 (ppm)

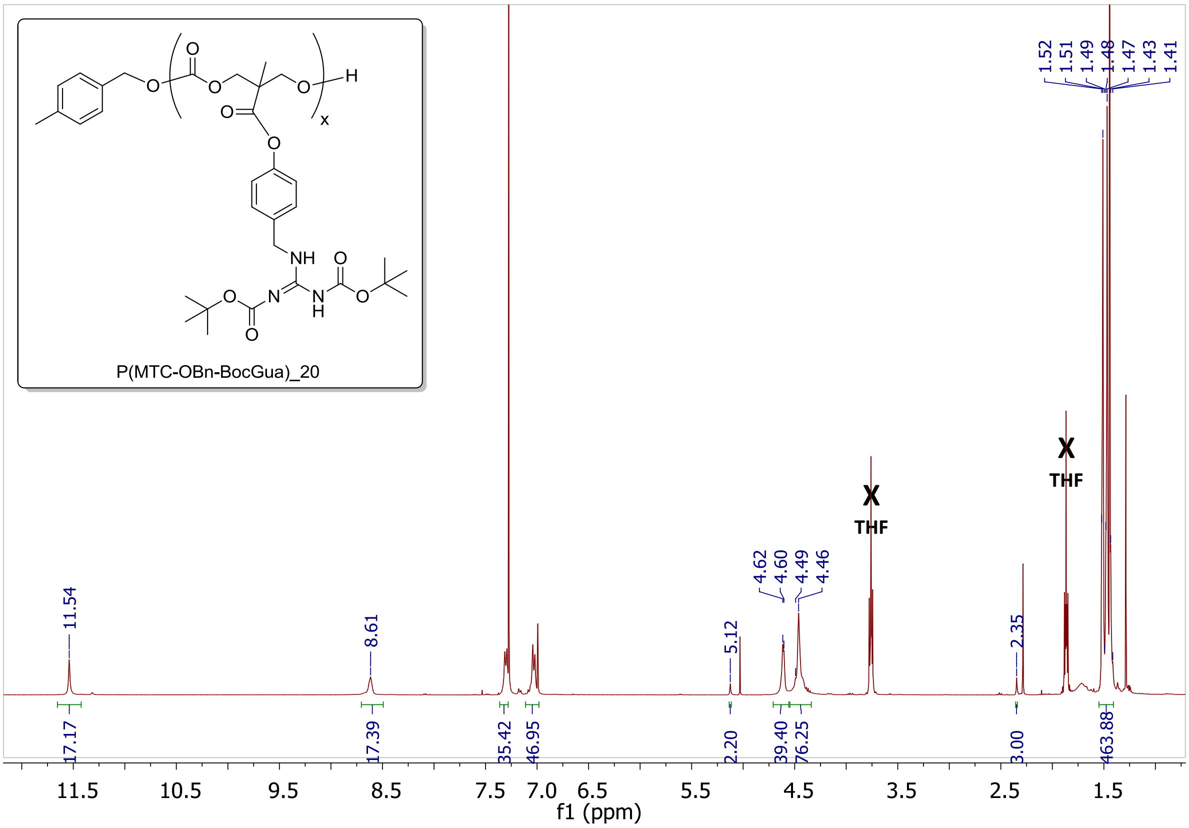
P(MTC-OBn-BocGua)_20
11.54
8.61
5.12
4.62
4.60
4.49
4.46
X
THF
2.35
1.52
1.51
1.49
1.48
1.47
1.43
1.41
17.17
17.39
35.42
46.95
2.20
39.40
76.25
3.00
463.88
11.5
10.5
9.5
8.5
7.5
7.0
6.5
5.5
4.5
3.5
2.5
1.5
f1 (ppm)

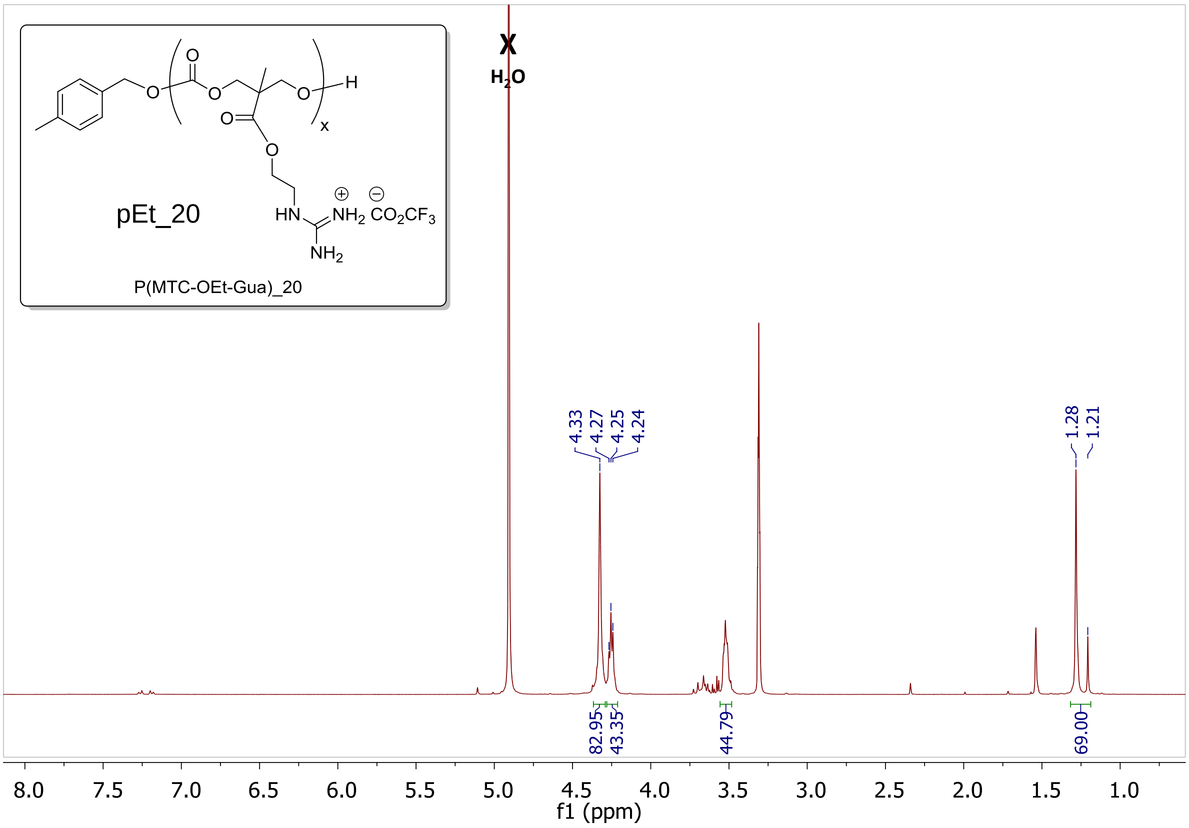
pEt_20
P(MTC-OEt-Gua)_20
X
H_2O
4.33
4.27
4.25
4.24
1.28
1.21
82.95
43.35
44.79
69.00
8.0 7.5 7.0 6.5 6.0 5.5 5.0 4.5 4.0 3.5 3.0 2.5 2.0 1.5 1.0
f1 (ppm)

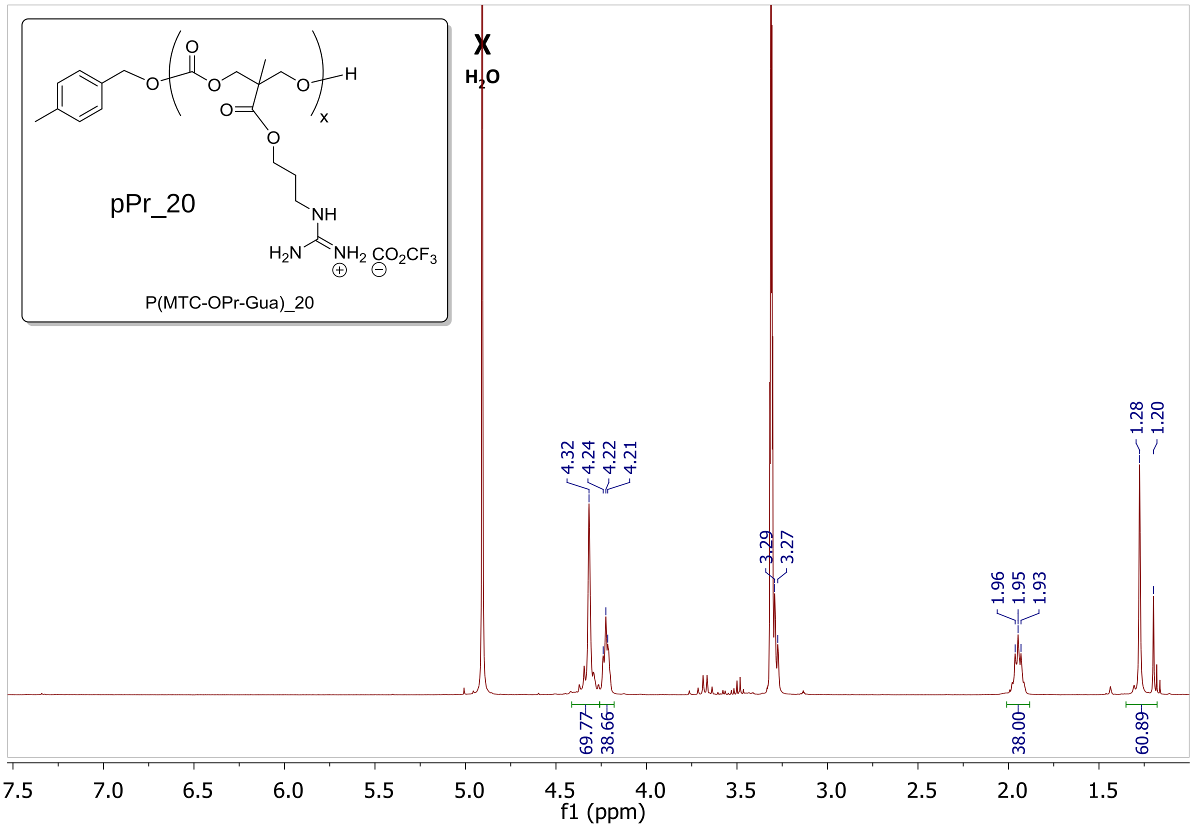
pPr_20
P(MTC-OPr-Gua)_20
X
H2O
f1 (ppm)

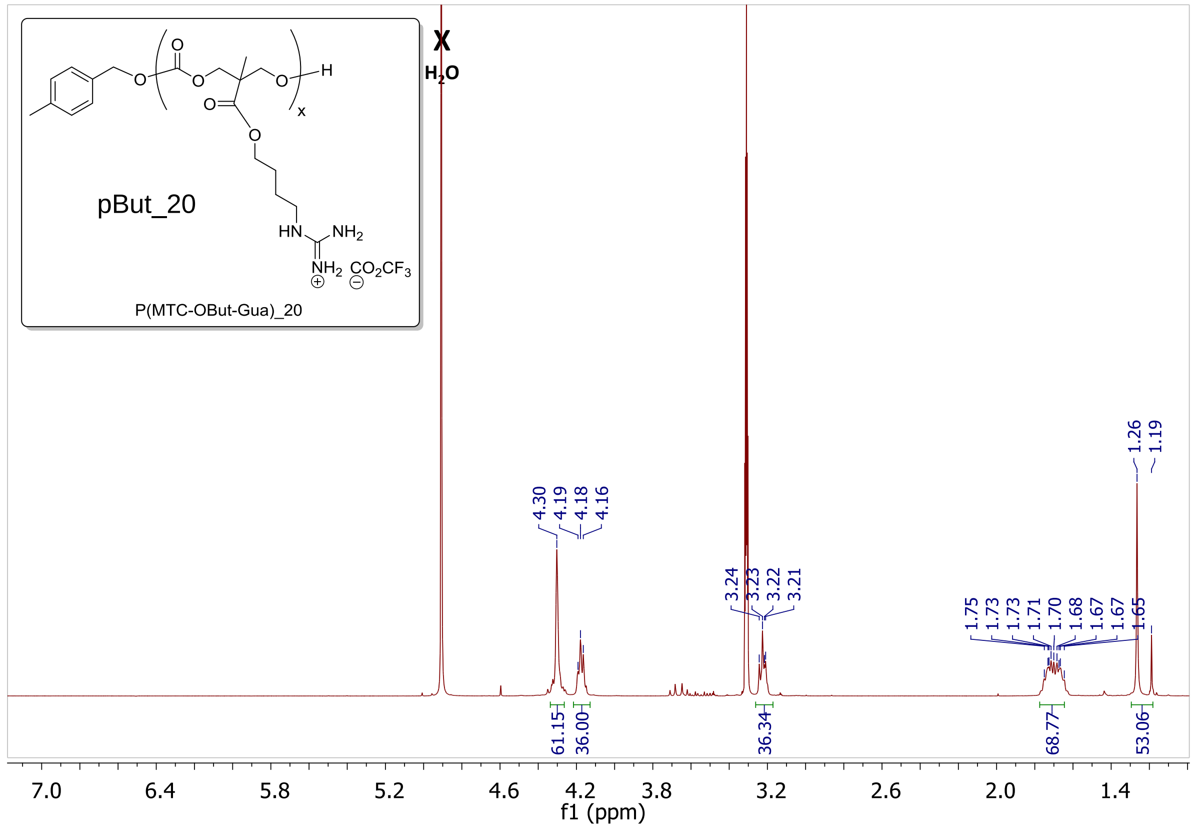
pBut_20
P(MTC-OBut-Gua)_20
X
H2O
f1 (ppm)

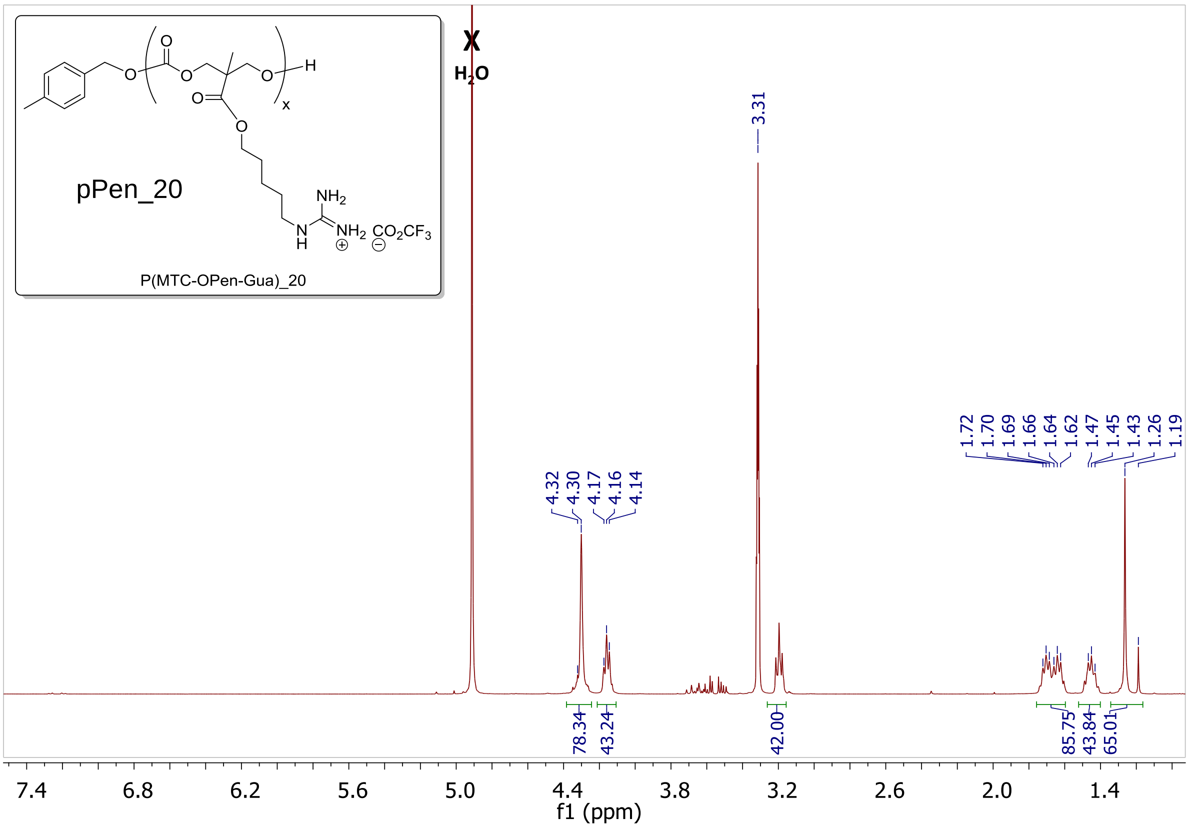

pPen_20
P(MTC-OPen-Gua)_20
X
H2O
3.31
4.32
4.30
4.17
4.16
4.14
1.72
1.70
1.69
1.66
1.64
1.62
1.47
1.45
1.43
1.26
1.19
78.34
43.24
42.00
85.75
43.84
65.01
7.4
6.8
6.2
5.6
5.0
4.4
3.8
3.2
2.6
2.0
1.4
f1 (ppm)

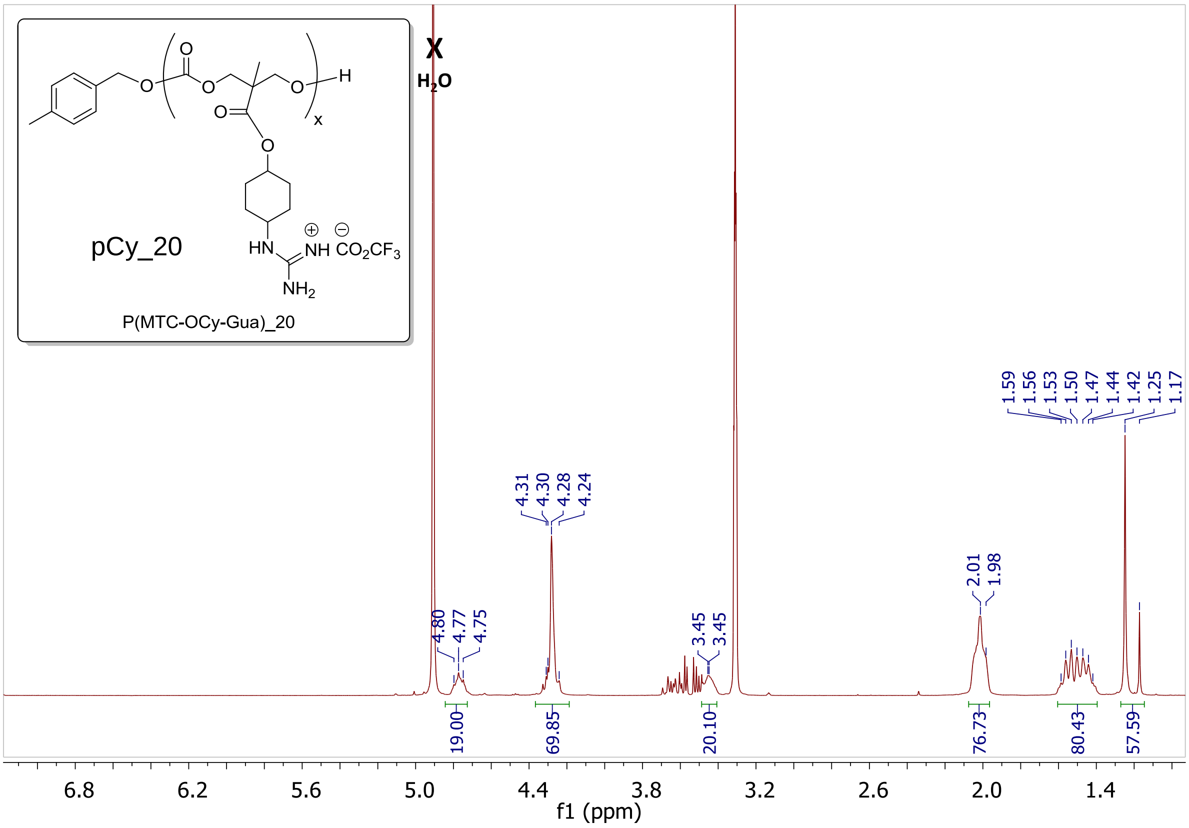

pCy_20
P(MTC-OCy-Gua)_20
X
H2O
4.80
4.77
4.75
4.31
4.30
4.28
4.24
3.45
3.45
2.01
1.98
1.59
1.56
1.53
1.50
1.47
1.44
1.42
1.25
1.17
19.00
69.85
20.10
76.73
80.43
57.59
6.8
6.2
5.6
5.0
4.4
3.8
3.2
2.6
2.0
1.4
f1 (ppm)

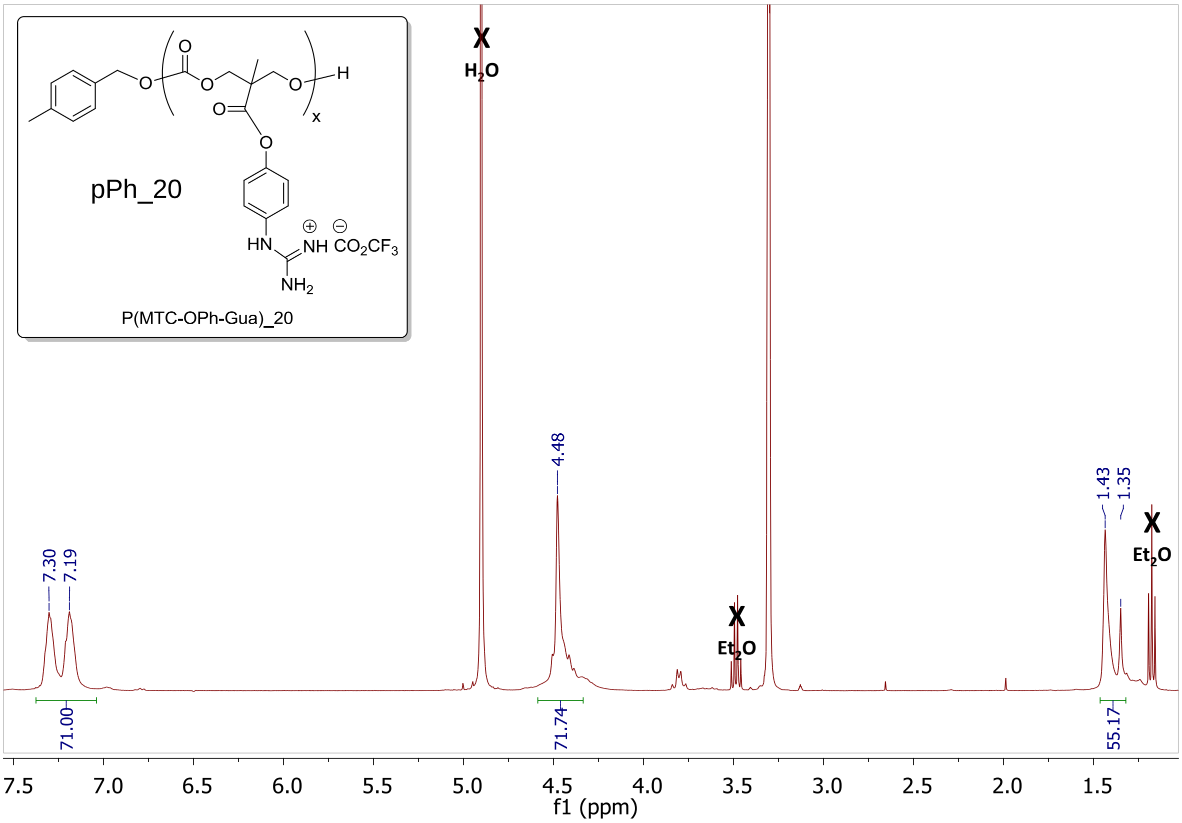

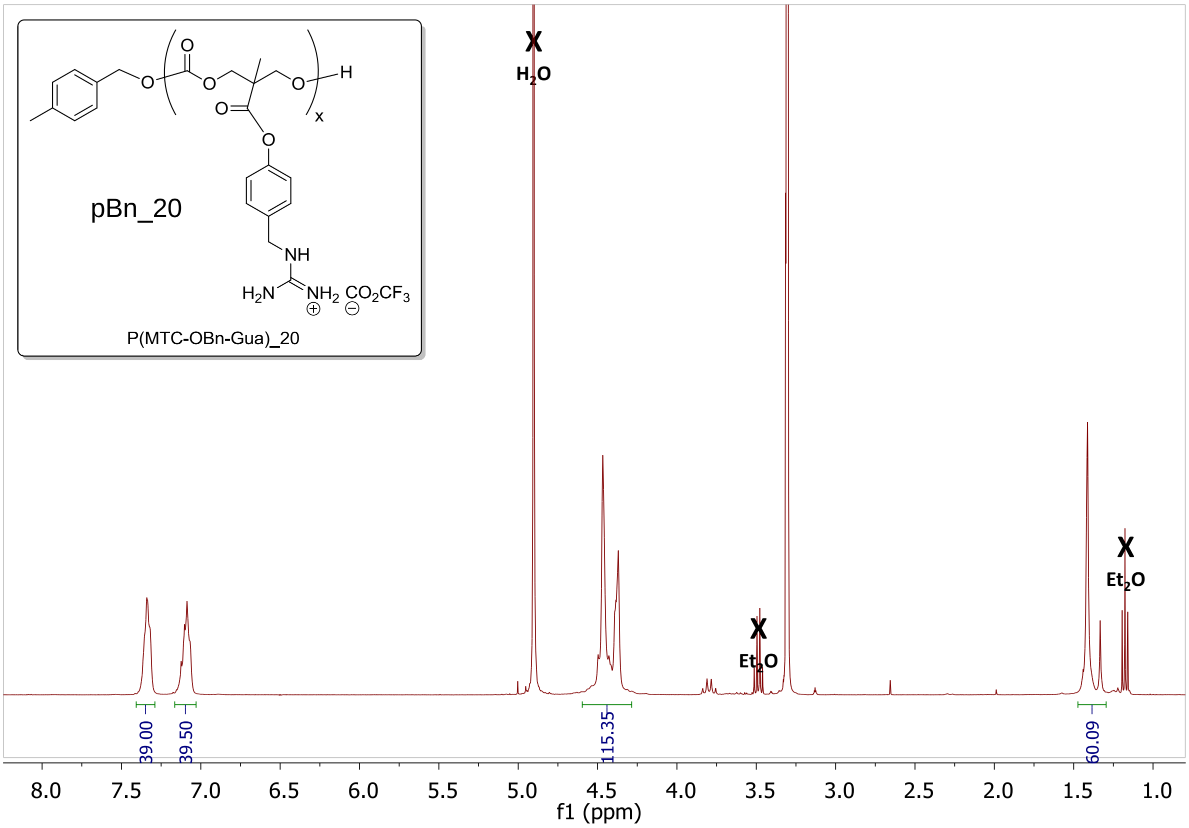

Supplementary Figure 1. ^{1}H NMR spectra of precursors, monomers and polymers.

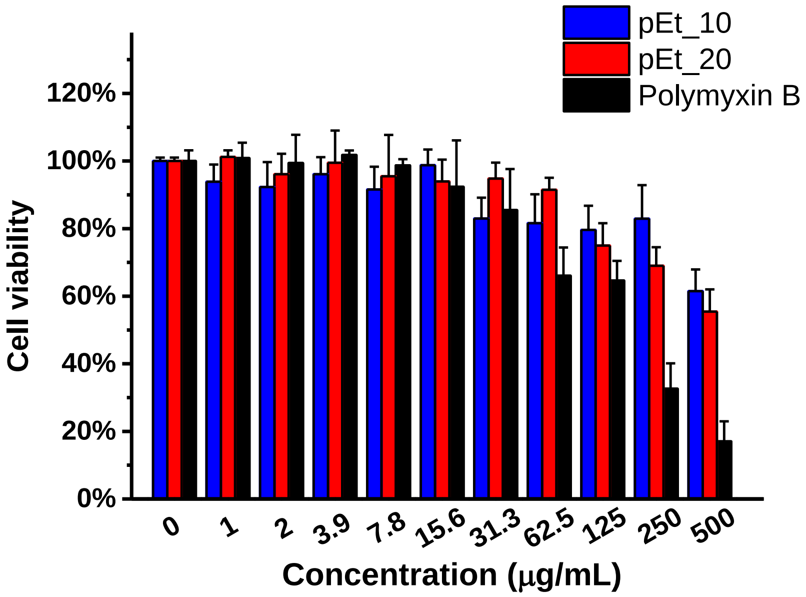

Supplementary Figure 2. Viability of HEK293T cells after 18-h incubation with pEt_10, pEt_20 and polymyxin B at 37°C, pH 7.4. Error bars represent s.d. for n=6.

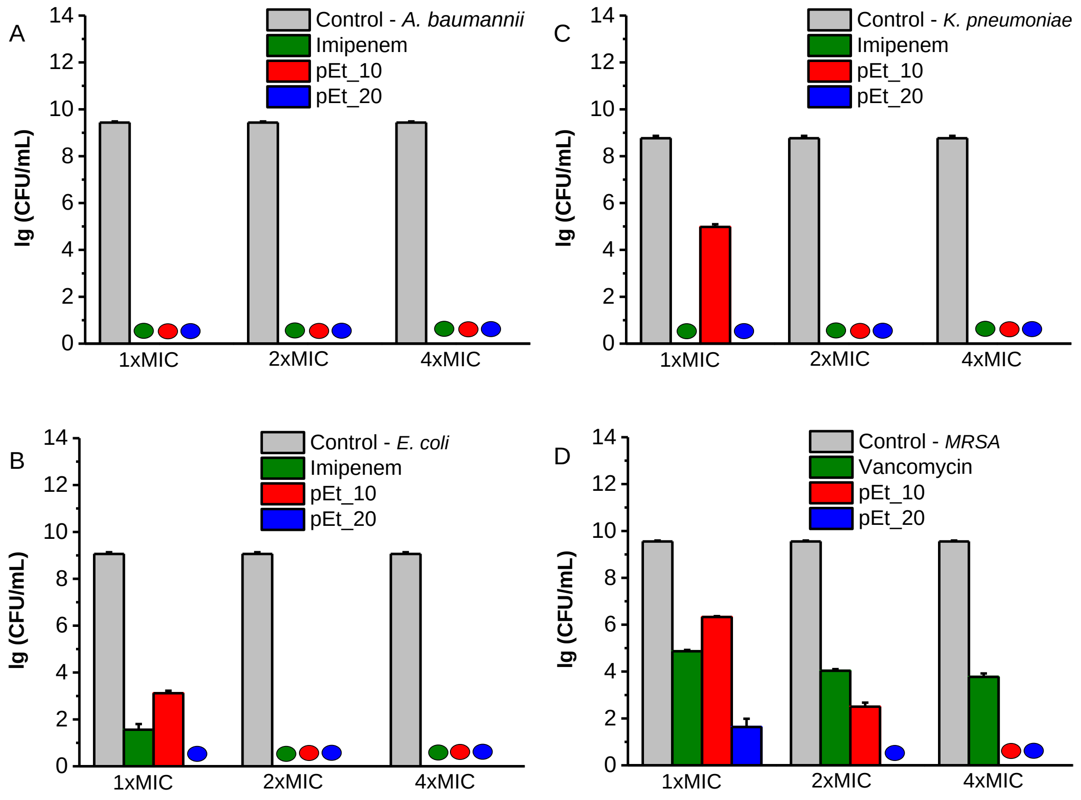

Supplementary Figure 3. Killing efficiency of pEt_10 and pEt_20 against *A. baumannii* 10073 (A), *E. coli* 56809 (B), *K. pneumonia* 8637 (C) and *MRSA* 25312 (D). Error bars represent s.d. for n=3.

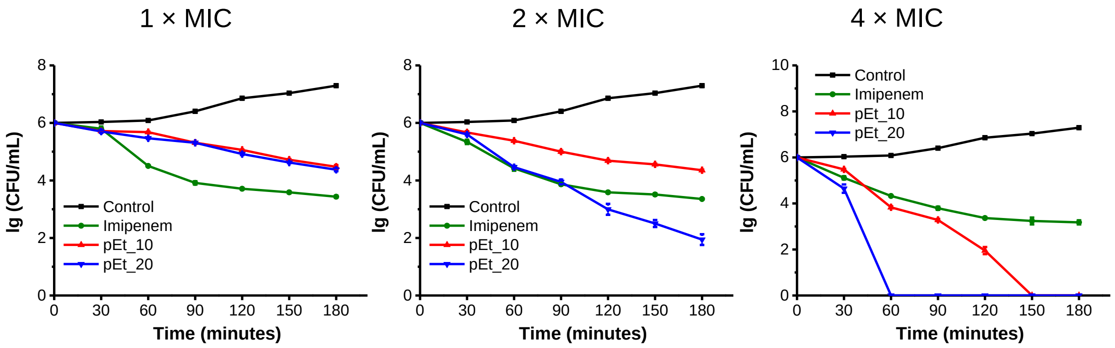

Supplementary Figure 4. Killing kinetics of *K. pneumoniae* 8637 at different concentrations as specified. The antibiotics imipenem did not exert bactericidal activity after 2 h treatment at 4×MIC (<99.9% bacterial removal), while the polymers eradicated the bacteria more rapidly (~99.99% killing efficiency at 2 h and ~100% killing efficiency at 1 h for pEt_10 and pEt_20, respectively). An increased polymer concentration led to faster killing efficiency. Error bars represent s.d. for n=3.

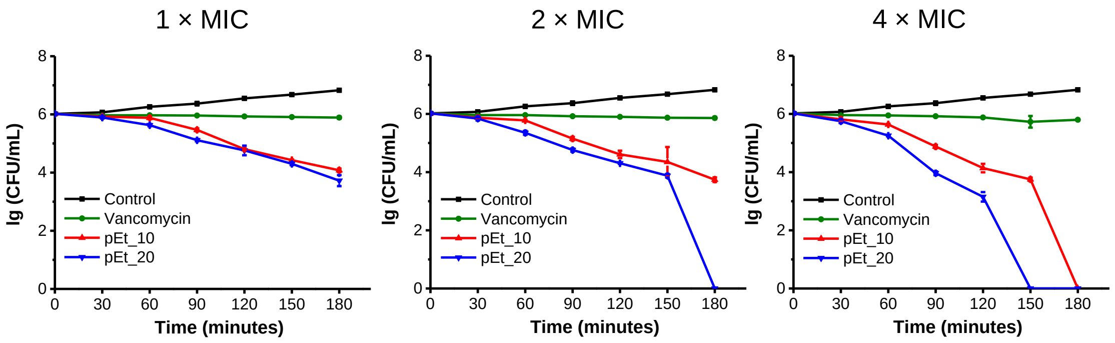

Supplementary Figure 5. Killing kinetics of *MRSA* 25312 at different concentrations as specified. The antibiotics vancomycin did not exert bactericidal activity after 3 h treatment, while the polymers eradicated the bacteria rapidly. An increased polymer concentration led to faster killing efficiency. Error bars represent s.d. for n=3.

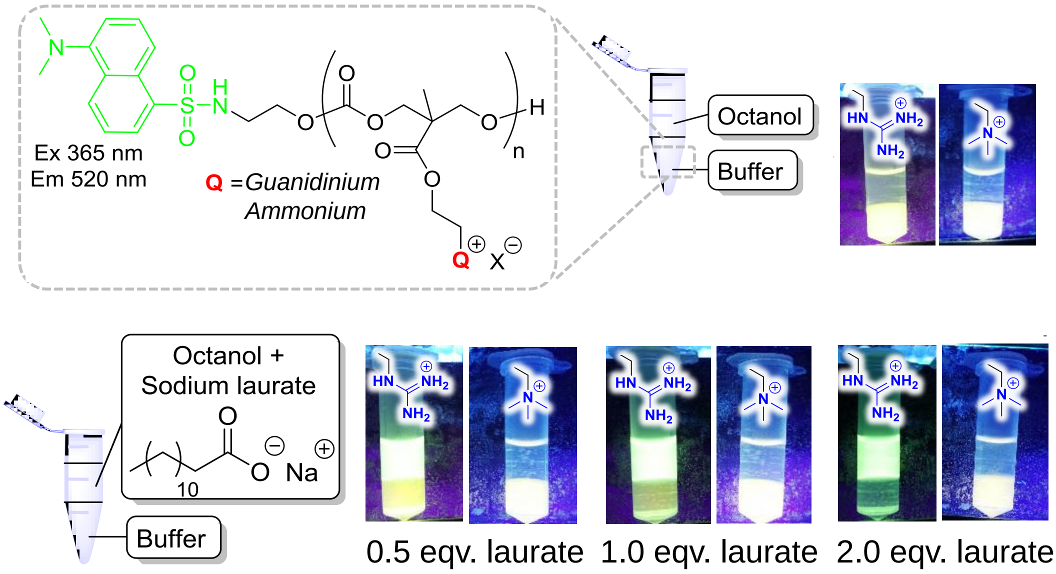

Supplementary Figure 6. Dansyl-functionalized pEt_20 and ammonium-containing polycarbonate were subjected to an octanol-water partition study to investigate the difference in the cationic groups towards binding with a complementary charge typically found on bacterial membrane surface (i.e. sulfate).

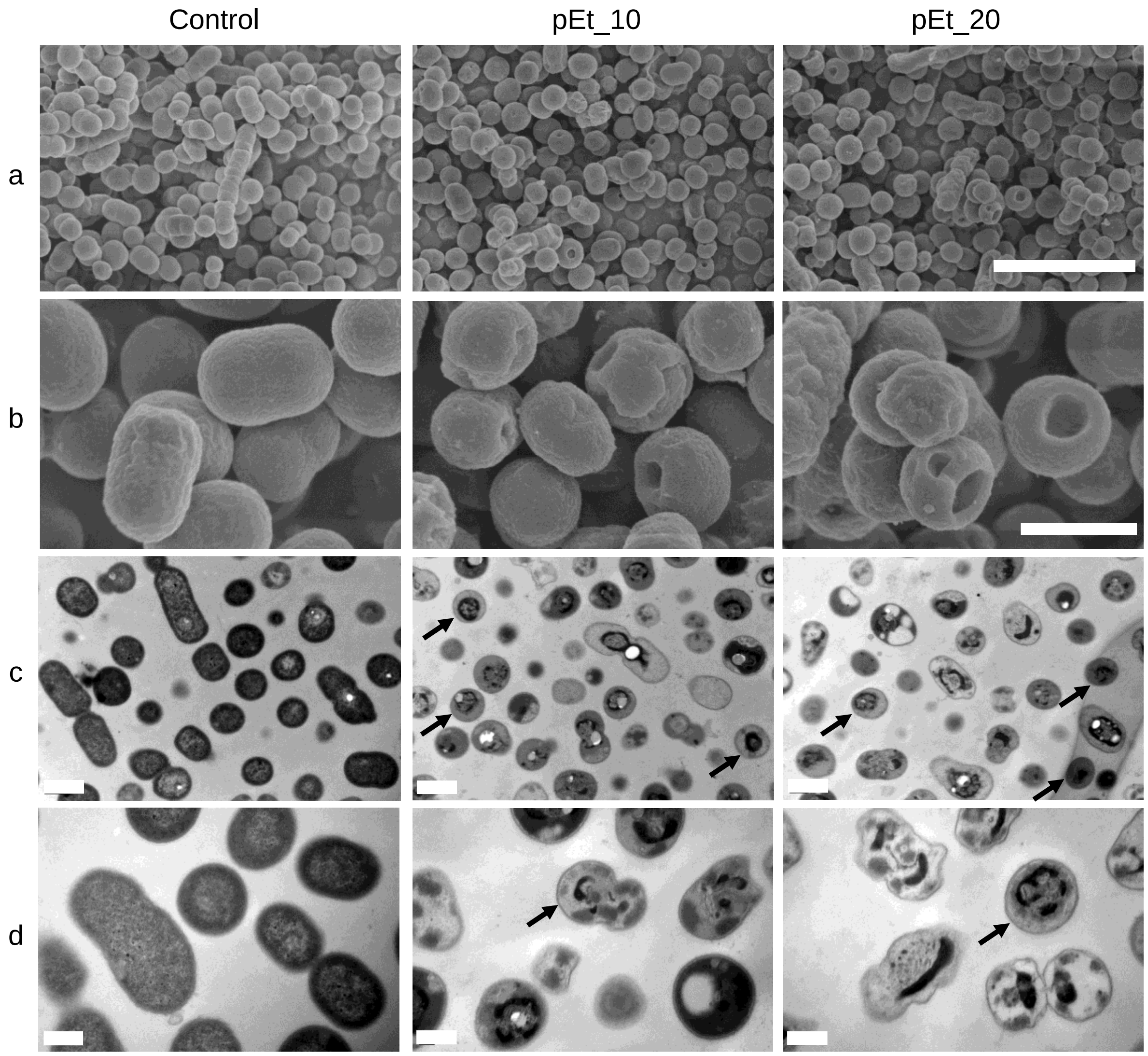

Supplementary Figure 7. Mechanistic studies *via* SEM and TEM. SEM (**a**, **b**) and TEM (**c**, **d**) images at different magnifications of *A. baumannii* before and after treatment with pEt_10 or pEt_20. Treatment conditions: 4×MIC, 6 h for SEM study; 16×MIC, 2 h for TEM study. Scale bar: SEM - 5 μm in **(a)** and 1 μm in **(b)**; TEM - 1 μm in **(c)** and 0.5 μm in **(d)**. Although longer treatment time and higher polymer concentration led to more cells with membrane disruption, a larger population of the cells had an intact membrane. Precipitation of cytoplasmic materials was seen in more cells with an intact membrane under TEM (see arrows).

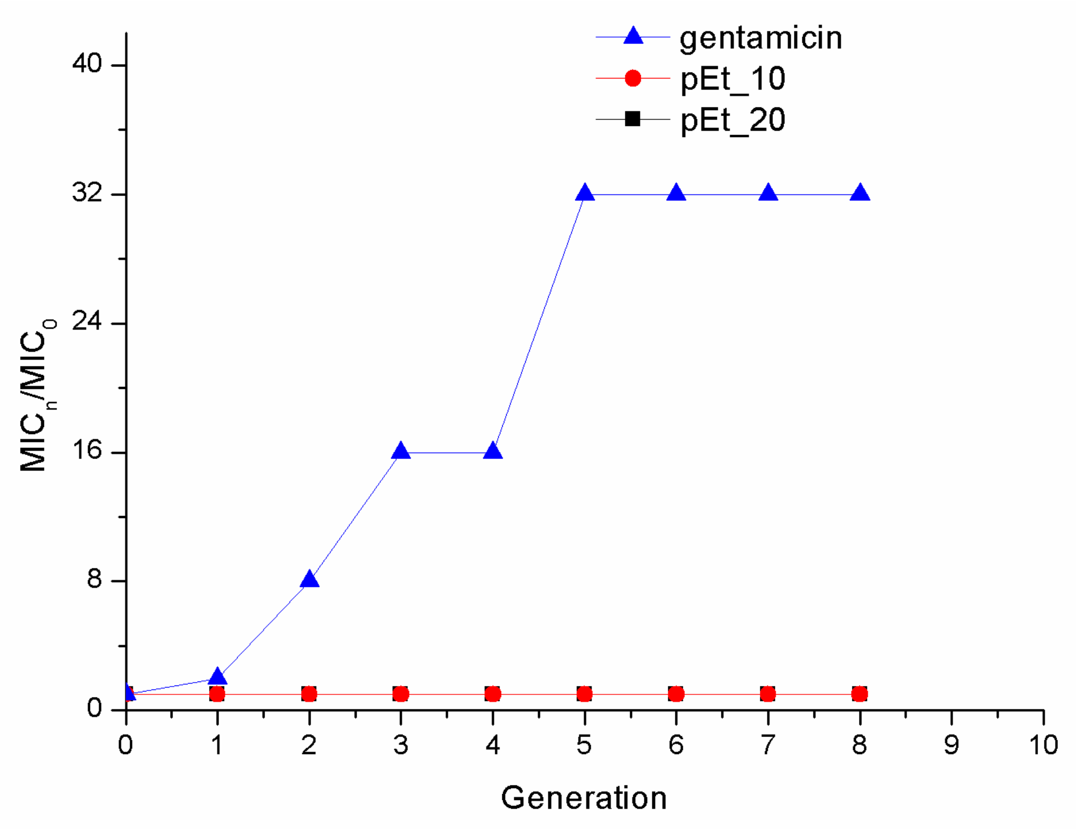

Supplementary Figure 8. Drug resistance development profiles of *Klebsiella pneumoniae* 8916 after exposed to the polymers and the clinically used antibiotic imipenem at sub-MIC concentrations. The data are representative of 3 replicates.

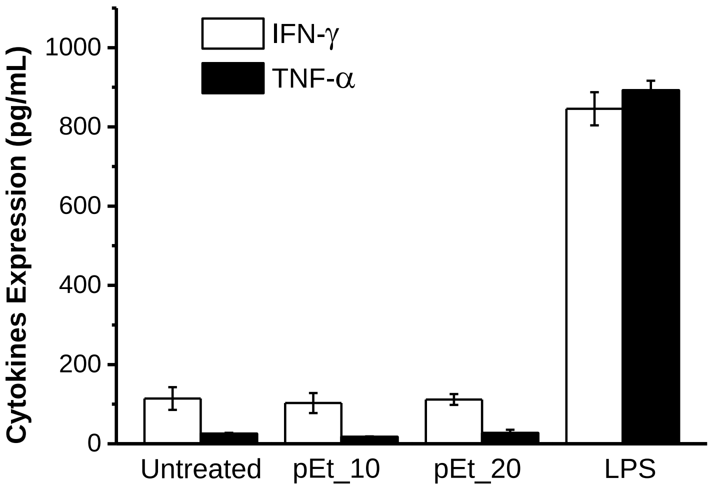

Supplementary Figure 9. Secretion of (□) IFN-γ and (■) IFN-α by mouse peripheral blood mononuclear cells stimulated by pEt_10, pEt_20 and LPS. Error bars represent s.d. for n=3.

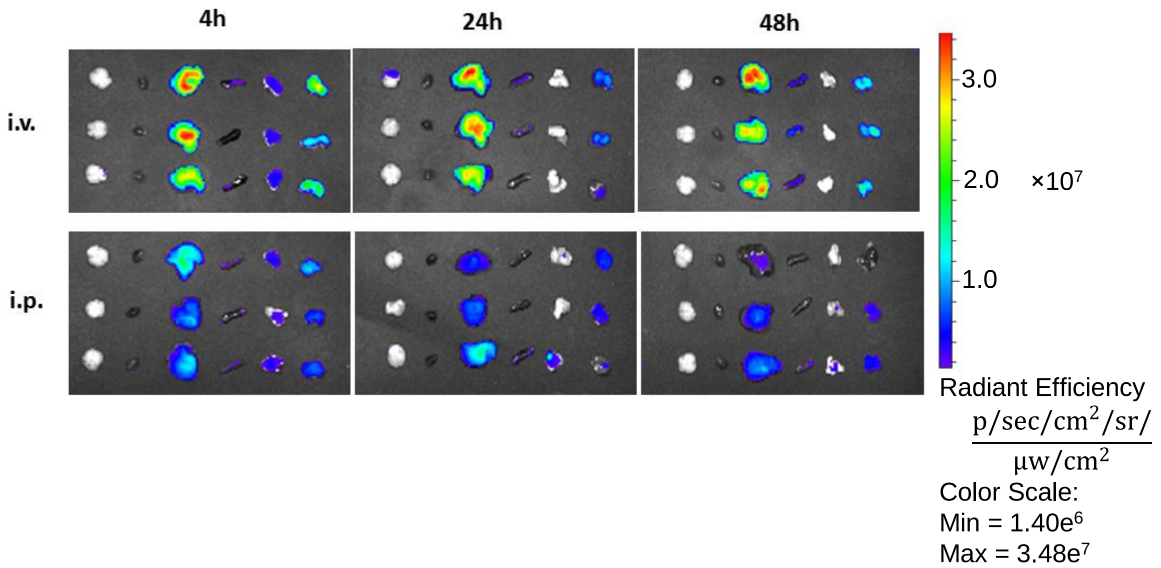

Supplementary Figure 10. Biodistribution of AF750-conjugated polymer delivered using different injection routes. At 4, 24 and 48h, the mice were sacrificed and organs including the brain, heart, liver, spleen, lungs, kidneys were excised and imaged. From left: Brain, heart, liver, spleen, lungs, kidneys. The polymer was found in mouse liver, spleen, lungs and kidneys after *i.p.* or *i.v.* injection, indicating that the polymer penetrated tissues and got into the blood stream even after *i.p.* injection.

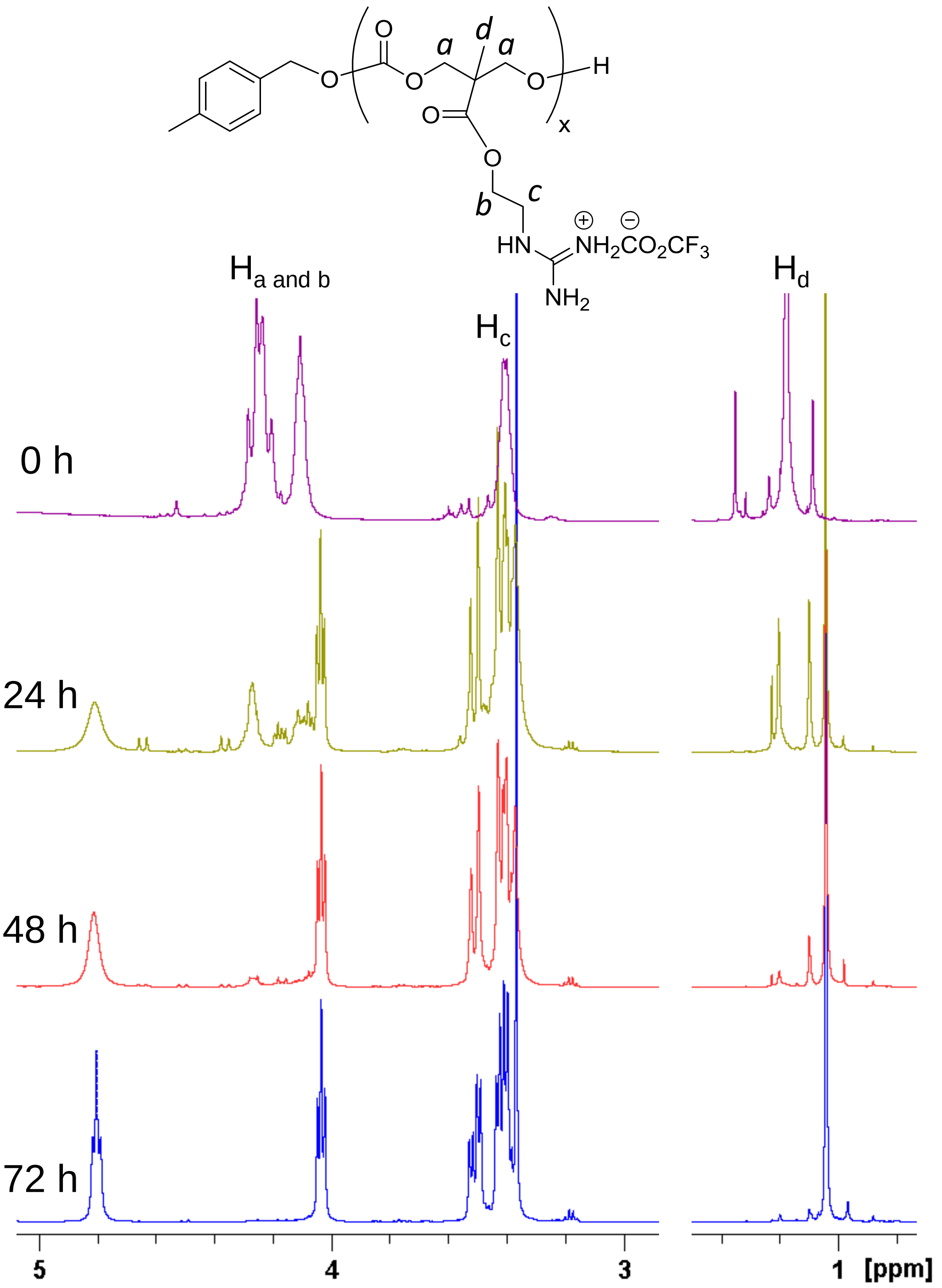

Supplementary Figure 11. ^{1}H NMR spectra of degrading pEt_20. The polymer was incubated at 37 °C in PBS (pH 7.4, a simulated physiological environment) for various periods of time as specified. PBS buffer was prepared in D_2O, which allowed for ^{1}H NMR analysis. The intensity of peaks at 4.4-4.0 ppm, which are attributed to the methylene groups of polycarbonate backbone and the methylene group next to the ester bond on the side chain, reduced significantly after 48 h and disappeared after 72 h. Meanwhile, the peak at 1.18 ppm, which corresponds to the methyl group of polycarbonate backbone, also declined significantly after 48 h, and disappeared after 72 h. These results demonstrate the degradation of the polymer.

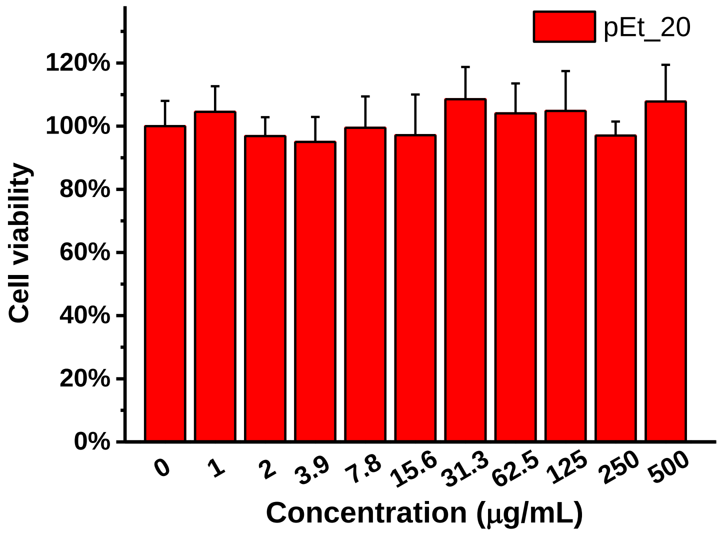

Supplementary Figure 12. Viability of HEK293T cells after 18-h incubation with degradation products of pEt_20 at 37°C, pH 7.4. The polymer pEt_20 was incubated in PBS (pH 7.4) for 3 days to ensure complete degradation (**Supplementary Figure 11**). Error bars represent s.d. for n=6.

Supplementary Table 1. Antimicrobial (MIC, μg mL^{-1}) and hemolytic (HC_{50}, μg mL^{-1}) activities of pEt_Y with varying molecular weights

Polymer	DP	MIC (μg mL^{-1})				G_m (μg mL^{-1})	HC_{50} (μg mL^{-1})	Selectivity (HC_{50}/G_m)
		S. aureus	*E. coli*	*P. aeruginosa*	*C. albicans*			
pEt_5	5	15.6	15.6	62.5	62.5	39.1	>8000[a]	>205
pEt_10	10	7.8	7.8	15.6	31.3	15.6	>8000[a]	>513
pEt_20	20	7.8	15.6	15.6	15.6	13.7	>8000[a]	>584
pEt_40	41	31.3	15.6	15.6	31.3	23.5	>8000[a]	>340

[a] At the highest concentration tested, extent of hemolysis was <5%.

Supplementary Table 2. Drug susceptibility of clinically isolated bacteria (S-Sensitive, I-intermediate, R-Resistant)*

Drugs/bacteria	*A. B.* *10073*	*A. B.* 9956	*E. Coli* 56809	*E. Coli* 58628	*K. P.* 8637	*K. P.* 8916	*MRSA* 25312	*MRSA* 25332
Cefoperazone/sulbactam					R	R		
Ampicillin/sulbactam	R	R			R	R		
Ciprofloxacin	R	R	S	R	R	R	R	R
Cefotetan	R	R			R	R		
Ceftriaxone	R	R	R	R	R	R		
Nitrofurantoin	R	R	S	S	R	R	S	S
Cefepime	R	R	S	S	R	R		
Gentamycin	R	R	R	S	R	I	R	I
Imipenem	R	R	S	S	R	R		
Levofloxacin	R	R	S	R	R	R	R	R
Trimethoprim/sulfamethoxazole (Chemitrim)	R	R	R	S	R	R	S	S
Ceftazidime	R	R			R	R		
Tobramycin (Nebcin)	R	R	I	R	R	S		
Cefazolin	R	R	R	R	R	R		
Amikacin			S	S	S	S		
Piperacillin/tazobactam	R	R	S	S	R	R		
Ampicillin	R	R	R	R	R	R		
Tigecycline			S	S	S	S	S	S
Aztreonam	R	R	S	S	R	R		
Ertapenem			S	S	R	R		
Erythromycin							R	R
Linezolid							S	S
Methicillin							R	R
Oxacilline							R	R
Benzylpenicillin (Penicillin G)							R	R
Rifampicin							S	S
Tetracycline							R	S
Teicoplanin							S	S
Vancomycin							S	S
Clindamycin							R	I
Moxifloxacin							R	R
Quinupristin/dalfopristin							S	S
Meropenem					R			
Cefuroxime					R			
Amoxicillin/clavulanic acid			I	S				
Cefoxitin			S	S				
Polymyxin B	R	R	S	S	R	R		

*According to the CLSI (Clinical Laboratory Standards Institute), drug susceptibility of bacteria is defined as follows:

MRSA: MIC of methicillin, S≤8 μg mL^{-1}, R≥16 μg mL^{-1}.

A. B. (*A. baumannii*): MIC of imipenem, S≤2 μg mL^{-1}, I=4 μg mL^{-1}, R≥8 μg mL^{-1}.

K. P. (*K. pneumonia*) and *E. coli*: MIC of imipenem, S≤1 μg mL^{-1}, I=2 μg mL^{-1}, R≥4 μg mL^{-1}.

K. P., A. B. and *E. coli*: MIC of polymyxin B, S≤2 μg mL^{-1}, R≥4 μg mL^{-1}.

Supplementary Table 3. MIC values of imipenem and vancomycin against the clinically isolated bacteria

Antibiotics	MIC (μg mL^{-1})							
	A. B. (strain no. 16523)	*A. B.* (Strain no. 9956)	*E. coli* (strain no. 56809)	*E. coli* (strain no. 58628)	*K. P.* (strain no. 8637)	*K. P.* (strain no. 8916)	*MRSA* (strain no. 25312)	*MRSA* (strain no. 25332)
Imipenem	16.0	16.0	0.25	0.25	64.0	64.0		
Vancomycin							0.5	0.5

*According to the CLSI (Clinical Laboratory Standards Institute), drug susceptibility of bacteria is defined as follows:
S. aureus: MIC of vancomycin, S≤2 μg mL^{-1}, I=4-8 μg mL^{-1}, R≥16 μg mL^{-1}.

Supplementary Table 4. Mean plasma concentrations of AF750-labelled pEt_20 in mice

Time (mins)	Mean plasma concentrations (n = 5) ng/mL ± SD
Predose	bql
2	23,674 ± 4,157
10	8,458 ± 1,158
15	4,282 ± 736
30	3,196 ± 923
60	1,641 ± 822
120	bql
240	bql
480	bql
bql: below quantitation limit	

Supplementary Table 5. PK indices of pEt_20 in mice

PK parameter	Units	1.3 mg/kg	
		Mean (ng/mL)	SD
C_i	ng/mL	23,674	4,157
$AUC_{o\text{-}inf}$	ng/mL*min	370,318	52,965
$T_{1/2}$	min	16.6	8.6
V_z	mL/kg	512	250
CL	mL/min/kg	22	3
C_i: concentration at T = 2 mins ; $AUC_{0\text{-}inf}$: area under the concentration-time curve at infinity; $T_{1/2}$: half-life of compound ; V_z: volume of distribution; CL: clearance			

Supplementary Table 6. Liver and kidney functions, sodium and potassium ion concentrations in the blood at 72 h after the first dose of polymer treatment in comparison with PBS treatment (n=6)

Treatment	ALT (U/L)	AST (U/L)	Creatinine µmol/L	Urea nitrogen mmol/L	Sodium ion mmol/L	Potassium ion mmol/L
PBS	28.9±3.5	85.0±9.1	19.2±3.6	8.6±1.1	147.5±2.3	4.5±0.3
pEt_10	30.7±3.4	80.0±12.2	20.2±3.1	9.4±1.3	148.2±2.8	4.7±0.6
pEt_20	29.4±4.2	88.5±10.9	17.8±10.9	8.0±1.1	148.5±2.5	4.8±0.5

P>0.05 for the groups treated with the polymers *versus* the group treated with PBS. Dose: 8 mg/kg, twice daily for 3 days by *i.p.* injection. The results indicated that the treatments with both polymers did not cause acute damage to the liver and kidneys and the electrolyte balance was not affected.

Supplementary Methods

^{1}H-NMR spectroscopy. ^{1}H-NMR spectra of the alcohol precursors, monomers and polymers were recorded on a Bruker Advance 400 NMR spectrometer at 400 MHz at ambient temperature. The ^{1}H-NMR measurements were carried out with an acquisition time of 3.2 s, a pulse repetition time of 2.0 s, a 30° pulse width, 5208-Hz spectral width, and 32 K data points. Chemical shifts were referenced accordingly to the respective solvent peaks, i.e. δ = 7.26 ppm for $CDCl_3$, 2.50 ppm for DMSO-d_6 and 3.31 ppm for CD_3OD.

GPC analysis. GPC analysis for the polymers was carried out with a Waters HPLC system equipped with a 2690D separation module with two Styragel HR1 and HR4E (THF) 5 μm columns (300 × 7.8 mm) in tandem series and a Waters 410 differential refractometer detector. The mobile phase used was THF with a flow rate of 1 mL/min. Number-average molecular weights as well as polydispersity indices were calculated from a calibration curve using a series of polystyrene standards with molecular weight ranging from 1,350 to 151,700.

MIC measurements. Microbes were cultured overnight in MHB at 37 °C (room temperature for *C. albicans*) under constant shaking of 100 rpm to reach exponential growth phase. The MICs of the polymers were measured using the broth microdilution method as described previously[1]. Briefly, 100 μL of MHB solution containing the polymer (with a fixed de-ionized (DI) water concentration of 20% v/v) at various concentrations (0-500 or 0-512 μg mL^{-1}) was placed into each well of a 96-well microplate. An equal volume of microbial suspension (3×10^5 or 10^6 CFU ml^{-1}) was added into each well. Prior to mixing, the microbial sample was first inoculated overnight to enter its log growth phase. The concentration of microbial solution was adjusted to give an initial optical density (O.D.) reading of approximately 0.07 at 600 nm wavelength on a microplate reader (TECAN, Switzerland),

which corresponds to the concentration of Mc Farland 1 solution (3×10^8 CFU ml^{-1}). The microbial solution was then further diluted to achieve an initial inoculum of 3×10^5 or 10^6 CFU ml^{-1}. The 96-well plate was kept in an incubator at 37 °C under constant shaking of 100 rpm for 18 h. The MIC was taken as the concentration of the polymer at which no microbial growth was observed with unaided eyes and the microplate reader at the end of 18 h incubation. For *C. albicans,* MIC readings were determined after a longer incubation time of 42 h. The MIC values of the polymers pEt_10 and pEt_20 were also measured in the presence of 2%, 10% and 40% fetal bovine serum (FBS) against ATCC bacterial strains and clinical MDR bacterial isolates, respectively. Broth containing microbial cells with or without FBS was used as the negative control, and each test was carried out in 6 replicates. The experiment was independently repeated at least three times.

Hemolysis assay. The toxicity of the polymers against mammalian erythrocytes was tested using fresh rat red blood cells (rRBCs). Briefly, rRBCs were diluted 25-fold in PBS to achieve 4% v/v of blood content. The polymers were dissolved in PBS at concentrations ranging from 0-4000 μg mL^{-1} by serial dilutions. Equal volumes of polymer solutions (100 μL) were then mixed with the diluted blood suspension (100 μL). The mixtures were then incubated at 37 °C for 1 h to allow for the interactions between rRBC and the polymers to take place. After that, the mixture was subjected to centrifugation (1000 g for 5 min, 4 °C), and 100 μL aliquots of the supernatant were pipetted into a 96-well microplate. The hemoglobin release was measured spectrophotometrically by measuring the absorbance of the samples at 576 nm using the microplate reader (TECAN, Switzerland). Two control groups were employed for this assay: untreated rRBC suspension (negative control), and rRBC suspension treated with 0.1% Triton-X (positive control). Each assay was performed in 4 replicates. The experiment was independently repeated three times. The percentage of

hemolysis was defined as follows: Hemolysis (%) = [(O.D.$_{576nm}$ of the treated sample – O.D.$_{576nm}$ of the negative control)/(O.D.$_{576nm}$ of positive control – O.D.$_{576nm}$ of negative control)] × 100%.

MTT assay. The cytotoxicity of pEt_10, pEt_20 and polymyxin B (Merck) was evaluated by MTT ((3-(4,5-dimethylthiazol-2-yl)-2,5-diphenyl tetrazolium) assay using human embryonic kidney HEK 293T cell line (ATCC). The cells were maintained in Dulbecco's Modified Eagle Medium (DMEM, Gibco) with 10% heat inactivated fetal bovine serum (FBS, Invitrogen) and 1% penicillin/streptomycin (Gibco) in an incubator at 37 ˚C with 5% CO_2. When the cells reached ~80% confluency, the cells were seeded in 96-well plates with cell density of 5×10^4 cells/well in 100 µL of DMEM, and incubated at 37 ˚C with 5% CO_2 for 24 h. The medium was then removed, and DMEM containing pEt_10, pEt_20 or polymyxin B at various concentrations (0-500 µg/mL, 100 µL) were added to the 96-well plates. After 18-h incubation, the medium was removed, and replaced with 100 µL of fresh DMEM and 20 µL of MTT solution at 5 mg/mL in PBS. After 4-h incubation, the MTT-containing DMEM was removed and 150 µL of DMSO were added. The plates were shaken for 5 min to dissolve the purple formazan crystals. The absorbance of the 96-well plates at 570 nm were recorded to calculate the cell viability by using the following formula: Viability (%) = [(O.D.$_{570nm}$ of the treated cells – O.D.$_{570nm}$ of the blank well without cells)/(O.D.$_{570nm}$ of untreated cells – O.D.$_{570nm}$ of blank well without cells)] × 100. Using the same protocol, potential cytotoxicity of the degradation products of pEt_20 was evaluated.

Killing efficiency and killing kinetics. *A. baumannii* 10073, *E. coli* 56809, *K. pneumoniae* 8637 and *MRSA* 25312 were inoculated and prepared according to the same procedure in the MIC measurement described above. The samples were treated with pEt_10 and pEt_20 at

different concentrations (MIC, 2 × MIC and 4 × MIC), and were incubated at 37 °C under constant shaking of 100 rpm. After 18 h of incubation, the bacteria samples were taken out from each well for a series of tenfold dilutions. The diluted bacterial solution (50 μL) was streaked onto an agar plate (MHB Agar from 1st Base). The plate was incubated for 24 h at 37 °C and counted for colony-forming units (CFU). The killing efficiency was consequently determined by the corresponding percentage bacterial cell survival (i.e. CFU of sample / CFU of control). The killing kinetics was investigated by counting CFU of the bacterial samples at predetermined time points after polymer treatment. Each assay was performed in triplicates. The experiment was independently repeated three times.

SEM analysis. The morphology of *A. baumannii* 10073 before and after the treatment with pEt_10 and pEt_20 was observed under a JSM-7400F scanning electron microscope (JEOL, Japan) as described previously[2]. *A. baumannii* 10073 growing in exponential phase were used and the suspension was incubated with the polymers at a concentration of 4×MIC for 6 h. The bacterial suspension without any treatment was used as the negative control. After the treatment, the solution was centrifuged (5000 rpm, 10 min) to remove the supernatant. The bacteria were fixed after overnight incubation at 4 °C with PBS containing 2.5% glutaraldehyde. After washing with PBS three times, the bacterial suspension was subjected to one hour incubation using 1% OsO_4 in PBS. The samples were washed three times with PBS, and dehydrated using a series of graded ethanol solutions (35, 50, 75, 90 and 100%). The samples were mounted on a copper tape, air-dried and sputter coated with platinum for observation under a FE-SEM setup (JEOL JSM-7400F, Japan).

TEM analysis. The morphological changes of *A. baumannii* 10073 before and after the treatment with pEt_10 and pEt_20 were observed under a JEM-1230 transmittance electron

microscope (JEOL, Japan) using an acceleration voltage of 80 kV according to a protocol described previously[3]. Briefly, *A. baumannii* 10073 growing in exponential phase were used and the suspension was incubated with the polymers at a concentration of 16×MIC for 2 h. The bacterial suspension without any treatment was used as the negative control. After the treatment, the solution was centrifuged (5000 rpm, 10 min) to remove the supernatant. The bacteria were fixed after overnight incubation at 4 °C with PBS containing 2.5% glutaraldehyde. After washing with PBS three times, the bacteria were subjected to one hour incubation with 1% OsO_4 in PBS. The samples were washed three times with PBS, and dehydrated using a series of graded ethanol solutions (35, 50, 75, 90 and 100%). The dehydrated samples were transferred to a 1:1 mixture of absolute acetone and Spurr resin for 1 h at room temperature and then to a 1:3 mixture of absolute acetone and resin for 3 h. Finally, the samples were transferred to Spurr resin and incubated overnight. Sections having thickness of 70-90 nm were obtained using a Reichert-Jung Ultracut E Ultra microtome, and post-stained with uranyl acetate and lead citrate for 15 min prior to TEM observations.

Assessment of polymer toxicity towards major organs and electrolyte balance. The ICR mice were randomly divided into three groups: PBS control group, pEt_10 and pEt_20 treated groups (six mice per group). Each mouse received *i.p.* injections of pEt_10 or pEt_20 (each dose: 8 mg/kg, 0.2 mL/20 g) twice daily for 3 consecutive days. The mice were then sacrificed and anesthetized to obtain blood samples from their periorbital plexus at 72 h after the first dose for analysis of liver (alanine transaminase-ALT, aspartate transaminase-AST) and kidney functions (creatinine, ureanitrogen), sodium ion and potassium ion levels.

Conjugation of AF750 (a NIR dye) to pEt_20. Alexa Fluor 750 dye (AF750, Thermo Fisher Scientific Inc., Waltham, U.S.A.) was conjugated to pEt_20 to study pharmacokinetics

and biodistribution. Briefly, P(MTC-OEt-BocGua)_20 (73.3 mg, 0.008mmol), AF750 (15 mg, 0.012mmol) and a catalytic amount of 4-(dimethylamino)pyridine (DMAP, 1.2 mg) were dissolved in 6 mL of dry DMSO, followed by addition of 48 µL of dicyclohexylcarbodiimide (DCC) solution (1.0 M in DCM, 0.048 mmol). After reacted for 2 days, the reaction solution was dialyzed against DMSO for three times and the mixture of MeOH and DCM (volume ratio 1:1) for 6 times sequentially, using a dialysis membrane with molecular weight cut-off (MWCO) of 3.5 kDa, to remove unconjugated dye. The solution in the dialysis bag was concentrated to dryness and the residue was re-dissolved in 20 mL of DCM. 2 mL of trifluoroacetic acid was added to the solution and it was stirred overnight. The solution was dialyzed against the mixture of MeOH and DCM (volume ratio 1:1) for 4 times using a dialysis membrane with MWCO of 2 kDa. The solution in the dialysis bag was then concentrated to dryness and dried *in vacuo*, producing AF750-pEt_20 as a dark blue solid.

To quantify the conjugation degree of AF750 to the polymer, 1 mg of the polymer was dissolved in 1 mL of HPLC grade water and diluted 20 times in order for the light absorbance at 750 nm to fall within the detection limit. The value of absorbance was then compared against a calibration curve of the unconjugated AF750 at 750 nm to determine the amount of dye present per unit mass (per mole) of the AF750-conjugated polymer. The dye to polymer molar ratio was determined to be 0.167:1.

Supplementary References

1. Chin, W., Yang, C., Ng, V. W. L., Huang, Y., Cheng, J., Tong, Y. W., Coady, D. J., Fan, W., Hedrick, J. L. & Yang, Y. Y. Biodegradable broad-spectrum antimicrobial polycarbonates: investigating the role of chemical structure on activity and selectivity. *Macromolecules* **46,** 8797-8807 (2013).

2. Engler, A. C., Tan, J. P. K., Ong, Z. Y., Coady, D. J., Ng, V. W. L., Yang,Y. Y. & Hedrick. J. L. Antimicrobial polycarbonates: investigating the impact of balancing charge and hydrophobicity using a same-centered polymer approach. *Biomacromolecules* **14,** 4331-4339 (2013).

3. Cheng, J., Chin, W., Dong, H., Xu, L., Zhong, G., Huang, Y., Li, L., Xu, K., Wu, M., Hedrick, J. L., Yang, Y. Y. & Fan, W. Biodegradable antimicrobial polycarbonates with *in vivo* efficacy against multidrug-resistant MRSA systemic infection. *Adv. Healthc. Mater.* **4,** 2128-2136 (2015).
