# Supplementary material for: A macromolecular approach to eradicate multidrug resistant bacterial infections while mitigating drug resistance onset
Source: Nat Commun. 2018 Mar 2;9:917. doi: 10.1038/s41467-018-03325-6 (PMC5834525; doi:10.1038/s41467-018-03325-6)
Supplement: Supplementary file 2 — Description of Additional Supplementary Files [file 41467_2018_3325_MOESM2_ESM.docx]

**Description of Additional Supplementary Files**

File Name: Supplementary Data 1

Description: RNASeq on imipenem treated *A. baumannii* cells after 30 passages, relative to untreated control (at the start of the passage)

File Name: Supplementary Data 2

Description: RNASeq on pEt_20 treated *A. baumannii* cells after 30 passages, relative to untreated control (at the start of the passage)
